# Towards a better understanding of idiopathic epilepsy through metabolic fingerprinting of cerebrospinal fluid in dogs

**DOI:** 10.1038/s41598-024-64777-z

**Published:** 2024-06-26

**Authors:** Fien Verdoodt, Sofie F. M. Bhatti, Karla Kragic, Luc Van Ham, Lynn Vanhaecke, Myriam Hesta, Lieselot Y. Hemeryck

**Affiliations:** 1 Equine and Companion Animal Nutrition, Department of Morphology, Imaging, Orthopedics, Rehabilitation and Nutrition, Faculty of Veterinary Medicine, Ghent University, Salisburylaan 133, 9820 Merelbeke, Belgium; 2 Small Animal Department, Faculty of Veterinary Medicine, Ghent University, Salisburylaan 133, 9820 Merelbeke, Belgium; 3 Laboratory of Integrative Metabolomics, Department of Translational Physiology, Infectiology and Public Health, Faculty of Veterinary Medicine, Ghent University, Salisburylaan 133, 9820 Merelbeke, Belgium

**Keywords:** Neuroscience, Neurological disorders

## Abstract

Cerebrospinal fluid metabolomics is a promising research technology in the elucidation of nervous system disorders. Therefore, in this work, a cerebrospinal fluid (CSF) metabolomics method using liquid chromatography coupled to mass spectrometry was optimized and validated to cover a wide range of metabolites. An acceptable coefficient of variance regarding instrumental, within-lab and intra-assay precision was found for 95, 70 and 96 of 102 targeted metabolites, together with 1256, 676 and 976 untargeted compounds, respectively. Moreover, approximately 75% of targeted metabolites and 50% of untargeted compounds displayed good linearity across different dilution ranges. Consequently, metabolic alterations in CSF of dogs with idiopathic epilepsy (IE) were studied by comparing CSF of dogs diagnosed with IE (Tier II) to dogs with non-brain related disease. Targeted metabolome analysis revealed higher levels of cortisol, creatinine, glucose, hippuric acid, mannose, pantothenol, and 2-phenylethylamine (*P* values < 0.05) in CSF of dogs with IE, whereas CSF of dogs with IE showed lower levels of spermidine (*P* value = 0.02). Untargeted CSF metabolic fingerprints discriminated dogs with IE from dogs with non-brain related disease using Orthogonal Partial Least Squares Discriminant Analysis (R^2^(Y) = 0.997, Q^2^(Y) = 0.828).

## Introduction

Metabolomics is a promising research technology with application increasing rapidly in both human and veterinary medicine during the last decades^1^. Metabolomics enables to characterize an individual’s biological phenotype, thereby reflecting the integration of host-specific factors such as disease and the microbiome, and external factors including environmental influences and diet^2^. As such, the metabolome can provide insights in multiple pathophysiological processes like e.g., neuroinflammation^3^ and assist in the elucidation of diagnostic and prognostic biomarkers or therapeutic targets. A multitude of metabolomics studies has been performed in a wide range of biofluids, including faeces, urine, blood and saliva^4–6^.

Cerebrospinal fluid (CSF) is essential in maintaining normal central nervous system (CNS) function by providing nourishment for and waste removal from the CNS tissue^7^. Due to the close relationship with the CNS, CSF possesses unique properties compared to other biofluids as it is the only one localized on the brain side of the blood–brain-barrier, potentially providing useful information on CNS metabolism and function^8^. As such, CSF represents a key matrix for elucidation of the multiple complex pathways involved in CNS diseases. To date however, only a handful metabolomics studies in CSF have been published^9,10^.

Dogs suffer from similar naturally occurring neurological diseases as humans, like idiopathic epilepsy^11^ (IE) and canine cognitive dysfunction (a natural model for Alzheimer’s disease in humans^12^). Moreover, a similar prevalence of epilepsy is reported in dogs, i.e. 0.5–0.82% in first line practices^13^ in comparison to a lifetime prevalence of 0.64% in humans^14^. Indeed, canine epilepsy can be a sentinel for human epilepsy for multiple reasons. Firstly, canine and human epilepsy share clinical traits, such as the occurrence of status epilepticus and behavioral comorbidities^15^. Secondly, the electrophysiological and pharmacological properties of human and canine epilepsy are very similar^11^. Lastly, environmental circumstances for pets are highly comparable to humans, since pets and their owner mostly share the same home^16^. As in humans with epilepsy, management with antiseizure medication is inadequate in one third of dogs suffering from IE^17^, resulting in a need for alternative therapeutic targets. New discoveries in canine CSF therefore have the potential to benefit both veterinary and human medicine.

Specifically in the context of epilepsy, there are few publications on CSF metabolomics in both dogs and humans. In dogs, two studies focused on epilepsy, and identified relevant metabolic alterations in the CSF of dogs with IE compared to healthy controls or compared to dogs with epilepsy resulting from a structural cerebral pathology^18,19^. Hasegawa et al. used gas-chromatography, targeting volatile metabolites. They observed a significant increase of 15 metabolites in the CSF of dogs with IE (n = 16) compared to healthy controls (n = 18) and 14 metabolites in dogs with IE compared to dogs with structural epilepsy (n = 19). Of these metabolites, only glutamic acid showed significant differences among all three groups^18^. Another study exclusively focused on the endocannabinoid system, whereby they found a significant increase in anandamide and decrease in 2-arachidonoylglycerol in the CSF of dogs with IE (n = 40) compared to healthy controls (n = 16)^19^.

However, the above-mentioned CSF metabolomics studies either do not justify the extraction method used^10^, extrapolate methods developed for use in other types of matrices without testing and evaluating them specifically in CSF^18,19^ or target a limited selection of metabolites, for which the method was specifically optimized^19^. The objective of any analytical measurement should be to provide reliable data, which is achieved by firstly optimizing the method, and secondly confirming its fit-for-purposeness^20^. The goal of the current study was to optimize a generic CSF extraction protocol, to cover a wide range of metabolites in both a targeted and untargeted fashion. To this extent, an existing analytical metabolomics method based on ultra-high performance liquid chromatography (UHPLC) coupled to hybrid quadrupole-Orbitrap high resolution mass spectrometry (HRMS), previously established and validated for multi-matrix purposes in faeces, urine, plasma and saliva, was adopted^4^. UHPLC-HRMS is considered the gold standard for metabolomics analysis. This is due to the combination of accurate mass measurements, sub-part-per-millions errors and additional selectivity and sensitivity provided by UHPLC to ensure accurate identification of predefined targeted metabolites and untargeted fingerprinting of all metabolites present^6^. The developed CSF metabolomics method was validated and subsequently applied to a client-owned cohort of dogs with IE (n = 8) and dogs with other, non-brain related diseases (n = 7) to assess relevant metabolic alterations.

## Results

### Optimization of CSF extraction

Extraction of the CSF metabolome was optimized through a sequential strategy of experimental designs (Supplementary Tables S1 and S2). The 2^4^ fractional screening design (FFD) demonstrated that starting volume, centrifugation time and extraction solvent exerted a significant impact (*P *value < 0.05) on 90 (87%), 51 (49%) and 37 (36%) of the 104 evaluated targeted metabolites, respectively. The polyvinylidene (PVDF) filtering step at the end of the extraction protocol significantly impacted the untargeted fingerprint (*P* value = 0.0045) but did only significantly impact 24 (23%) of the evaluated targeted metabolites. The highest number of untargeted metabolic components found with PVDF-filter was 868, compared to 1020 without. However, based on in house expertise, a PVDF-filter was included to reduce potential clogging of the UHPLC-column. In a second phase, the extraction solvent ratio was optimized by varying the ratio of three different solvents using a two-step mixture design (MD). In the first MD, a combination of acetonitrile (ACN), acetone and methanol was assessed. Evaluation of the ternary plot revealed a significant effect on metabolome coverage (*P* value < 0.00001), showing better results with a lower ACN fraction. This MD was repeated, whereby a ratio combining ultrapure water (UPW), acetone and methanol was tested. The most optimal ratio consisted of 30% UPW, 10% acetone and 60% methanol. In a final step, optimal settings for starting volume, centrifugation time and speed were determined based on the results of the response surface modelling (RSM). Only the starting volume significantly impacted both targeted (*P* value = 0.02) and untargeted (*P* value = 0.0007) metabolome coverage. A positive impact was detected towards a higher starting volume, with a plateau for desirability reached at 350 µl. Centrifugation positively impacted outcome when a longer duration (15 min) and lower speed (5000×*g*) were used.

### Validation of CSF analysis method

We pursued validation of our optimized CSF metabolomics methodology based on linearity and precision as performance characteristics, for both targeted profiling and untargeted fingerprinting (Table 1, Supplementary Table S3). Targeted evaluation of the 9-point calibration curve showed good and excellent linearity across different dilution ranges for 80 (78%) and 55 (53%) of 102 metabolites, respectively. Untargeted fingerprints were evaluated in an identical manner, showing good linearity across different dilution ranges for 624 (54%) of metabolic components. Instrumental precision was compliant with the set coefficient of variance (CV) cut-offs indicating good precision (CV < 15%) for 90 (87%) of the targeted metabolites and acceptable precision (CV < 30%) for 1256 (85%) of the untargeted metabolic components, indicating high reproducibility for both the targeted and untargeted approach. Furthermore, repeatability was assessed by means of intra-assay and within-lab precision. Intra-assay precision showed a good CV for 87 (85%) of the targeted metabolites and an acceptable CV for 973 (66%) of the untargeted metabolic components, respectively. Within-lab precision showed a good CV for 60 (58%) of the targeted metabolites and acceptable CV for 676 (46%) untargeted metabolic components, respectively.

**Table 1:** Summary of validation performance characteristics of the CSF metabolomics method. For both the targeted and untargeted analysis, the number of analytes that meets the performance metric is reported in the left column. The percentage in the right column refers to the percentage of analytes that is represented by these total numbers.

	Absolute number		Percentage	
	Targeted	Untargeted	Targeted	Untargeted
Linearity				
	R^2^ > 0.90; R^2^ > 0.99	R^2^ > 0.90	R^2^ > 0.90; R^2^ > 0.99	R^2^ > 0.90
Dilution range: 0.002—1	79; 48	570	77%; 47%	49%
Dilution range: 0.02—1	80; 55	624	78%; 54%	54%
Instrumental precision				
CV < 15%	90	na	87%	na
CV < 20%, CV < 30%	95	1256	92%	85%
Intra-assay precision				
CV < 15%	87	na	85%	na
CV < 20%, CV < 30%	96	976	93%	66%
Within-lab precision				
CV < 15%	60	na	58%	na
CV < 20%, CV < 30%	70	676	68%	46%

### Study in dogs with idiopathic epilepsy

#### Targeted metabolites

**Figure 1:**
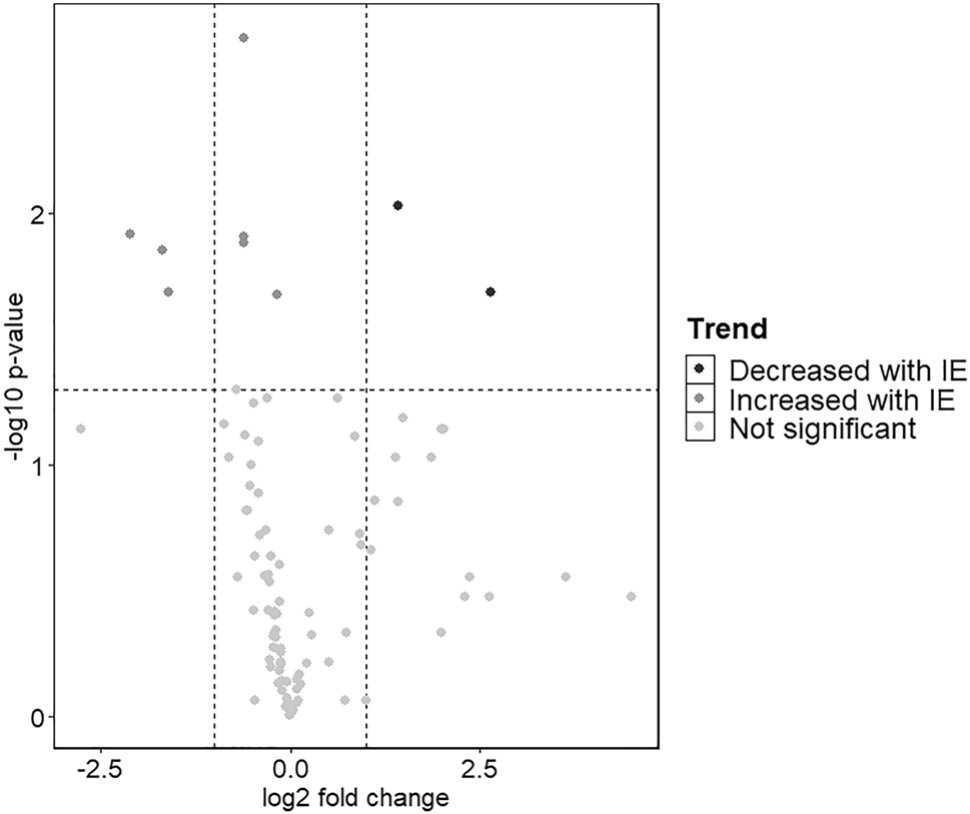
Volcano plot displaying the targeted metabolites analyzed in the CSF of dogs with IE compared to control dogs. A negative log2 fold-change indicates a lower signal in the control group compared to the IE group.

**Figure 2:**
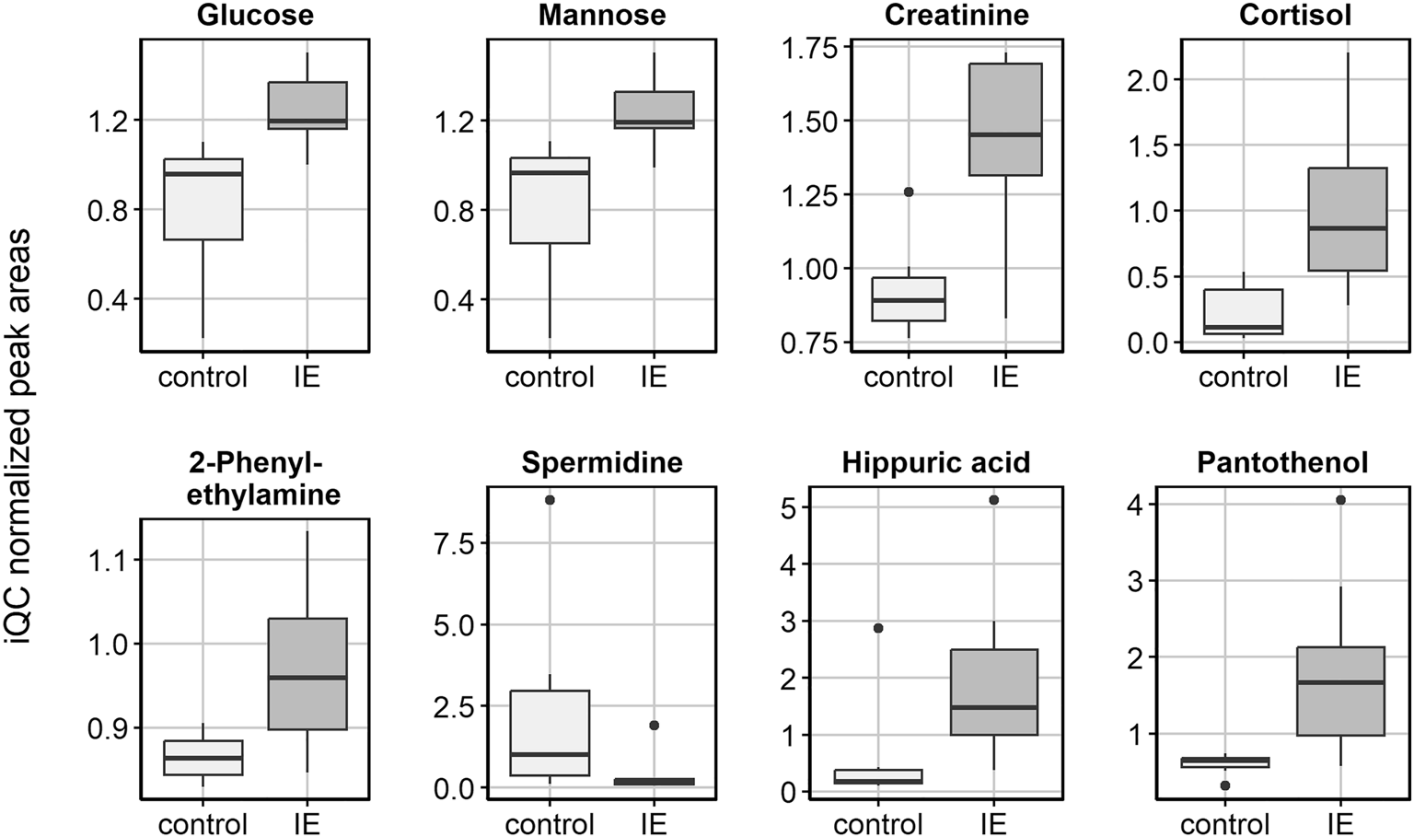
Boxplots of altered targeted metabolites (*P* value < 0.05) in the CSF of dogs with IE compared to dogs with non-brain related disease.

The presence of 101 metabolites was assessed in the client-owned dog cohort samples, for which population characteristics are displayed in Tables 2 and 3. Routine CSF analysis was available for 11/15 CSF samples, whereby all analyzed parameters were within normal limits. Total nucleated cell count was 0–2.75 cells/µl (reference range: 0–5 cells/µl), no hemodilution was seen, and total protein was additionally evaluated and normal in one atlanto-occipital sample (24.7 mg/dl; reference range < 25 mg/dl) and three lumbar samples (22.6–33.5 mg/dl; reference range < 40 mg/dl). Within these CSF samples (n = 15) 94 out of 101 metabolites were confirmed in all samples, and 7 demonstrated missing values in at least one of the samples. For the 94 consistently detected metabolites, statistical differences between dogs with IE compared to controls were assessed (Fig. 1, Supplementary Table S4). Cortisol (*P* value = 0.01; IE: 0.99 ± 0.64 vs. control: 0.23 ± 0.22), pantothenol (*P* value = 0.02; IE: 1.82 ± 1.16 vs. control: 0.60 ± 0.14), hippuric acid (*P* value = 0.01; IE: 1.93 ± 1.56 vs. control: 0.60 ± 1.01), creatinine (*P* value = 0.002; IE: 1.43 ± 0.30 vs. control: 0.93 ± 0.17), glucose (*P* value = 0.01; IE: 1.24 ± 0.17 vs. control: 0.81 ± 0.96), mannose (*P* value = 0.01; IE: 1.24 ± 0.33 vs. control: 0.81 ± 0.17) and 2-phenylethylamine (*P* value = 0.02; IE: 0.97 ± 0.10 vs. control: 0.87 ± 0.03) showed significantly higher normalized peak areas in the CSF of dogs with IE compared to controls (Fig. 2). Of these, only cortisol, hippuric acid and pantothenol showed a log2 fold change exceeding |1|. In contrast, the levels of spermidine (*P* value = 0.02; IE: 0.38 ± 0.62 *vs.* control: 2.37 ± 3.1) and acetophenone (*P* value = 0.009; IE: 1.03 ± 0.03 *vs.* control: 2.77 ± 4.46) were significantly lower in the CSF of dogs with IE compared to controls, with a log2 fold change exceeding |1|. For the latter, the boxplot revealed that significance was driven by one outlier, and therefore, findings for acetophenone were disregarded (Supplementary Fig. S1).

**Table 2:** Population characteristics of the dogs with IE included in the study cohort. *M* male, *MC* male castrated, *F* female, *FC* female castrated, CSF collection and seizure onset refer to the age of the dog in years at the time of sample collection or the occurrence of the first seizure, respectively. Days since last seizure refer to the number of days recorded between the last seizure and CSF collection of a particular dog.

Breed	Sex	CSF collection (years old)	Seizure onset (years old)	Days since last seizure	Monthly seizure frequency	Antiseizure medication
Crossbreed	MC	9	6	76	2.3	Imepitoine
Maltese dog	MC	2	1.5	6	4.5	Levetiracetam
Golden Retriever	FC	6.5	2	14	1.7	Phenobarbital, potassium bromide
Border Collie	FC	6	6	21	1.5	Levetiracetam
Maltese dog	M	1	0.5	14	0.75	Levetiracetam
Saint Bernard dog	M	1.5	1.5	28	1.5	Phenobarbital
Border Collie	M	8.5	6	1	0.5	Phenobarbital, levetiracetam
Malinois	M	3.5	3	6	1.5	Phenobarbital, levetiracetam

**Table 3:** Population characteristics of the control dogs included in the study cohort. *M* male, *MC* male castrated, *F* female, *FC* female castrated; CSF collection refers to the age of the dog in years at the time of sample collection.

Breed	Sex	CSF collection (years old)	Medical condition	Collection site
German Shepherd	M	12	Systemic neoplasia	Atlanto-occipital
French Bulldog	FC	5.5	Osteosarcoma thoracal spine	Atlanto-occipital
French Bulldog	FC	7.5	Disc extrusion cervical spine	Atlanto-occipital
German Pointer	M	13	Osteosarcoma left tibia	Atlanto-occipital
Great Dane	M	7	Lumbosacral stenosis	Lumbar
Anatolian Shepherd	M	1	Suspicion of polyarthritis	Lumbar
German Shepherd	F	1	Ataxia hind limbs, no cause identified	Lumbar

### Untargeted metabolic fingerprints

**Figure 3:**
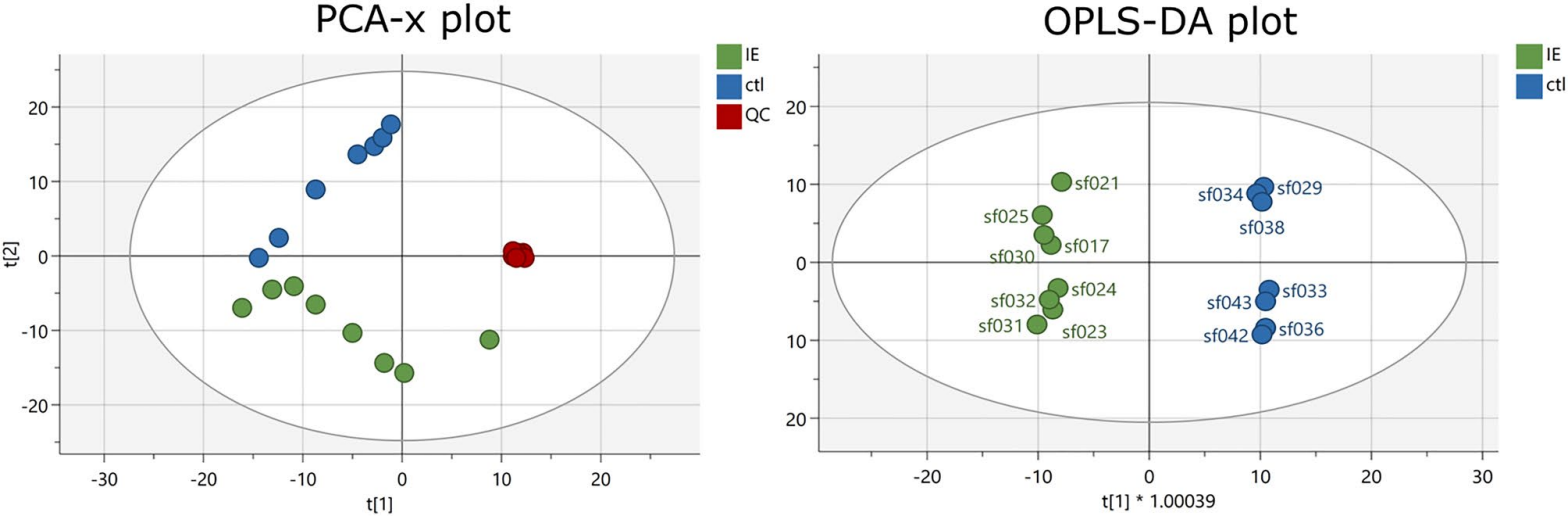
PCA-X score plot (unsupervised) and OPLS-da score plot (supervised) of the CSF metabolome fingerprint in dogs with IE in green *vs.* the control group (ctl) in blue, including QCs in red in the PCA-x score plot.

**Figure 4:**
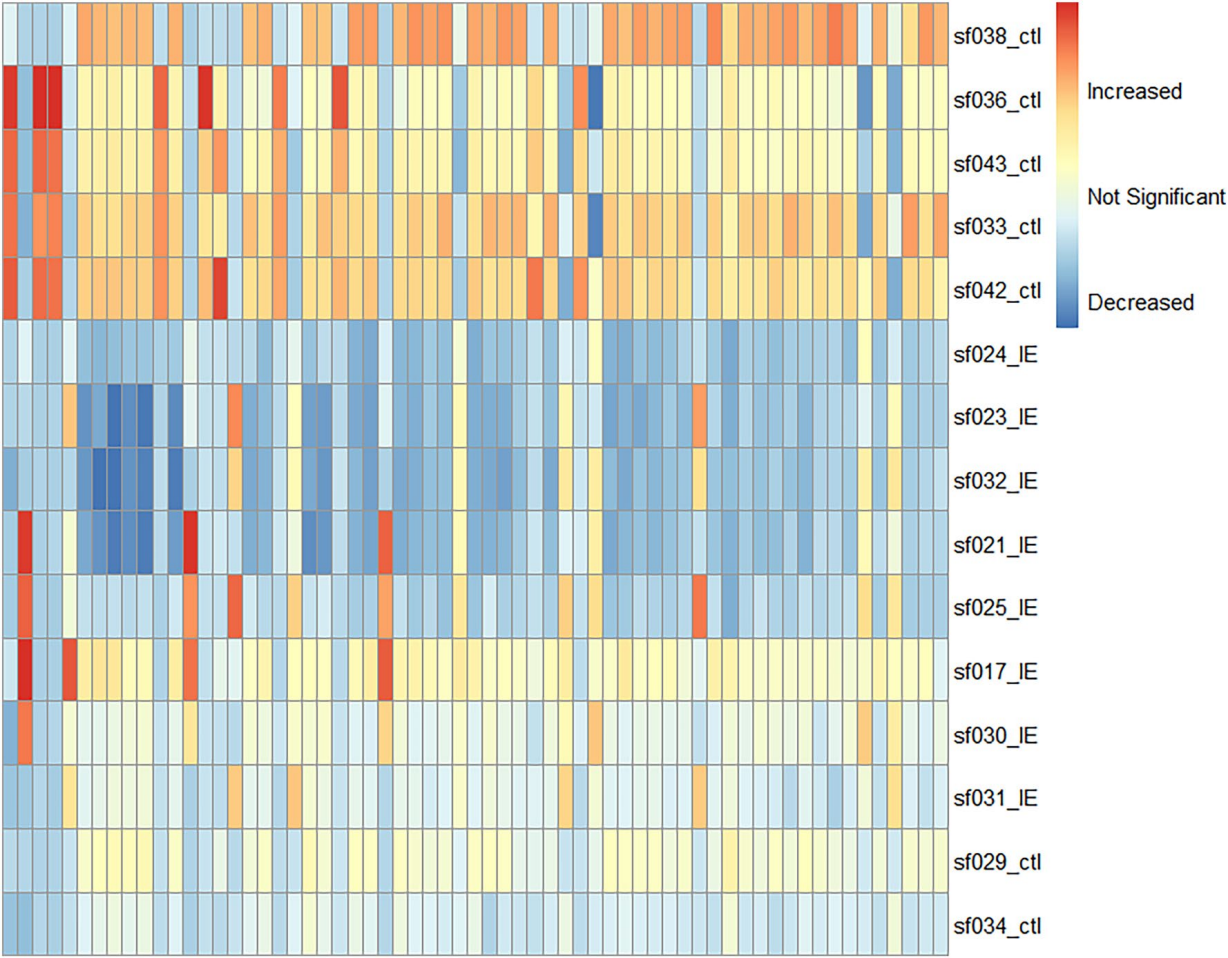
Heatmap displaying all untargeted discriminating components (n = 63) obtained from the IE group vs. ctl. Each column represents a specific untargeted component. Each row indicates one CSF sample, for which the sample name and group are indicated on the heatmap (ctl vs. IE). This figure was created by the authors using the R pheatmap package (Kolde R (2019). _pheatmap: Pretty Heatmaps_. R package version 1.0.12, https://CRAN.R-project.org/package=pheatmap).

CSF fingerprints covered 1020 components in positive and negative ionization mode (Supplementary Fig. S2). Firstly, an unsupervised PCA-X model was built to assess quality control (QC) clustering, showing clear separation between groups (Fig. 3). Secondly, Orthogonal Partial Least Squares Discriminant Analysis (OPLS-DA) modelling was performed to model the CSF metabolome in dogs with IE *vs.* dogs with non-brain related disease (i.e. the control group); with the model being compliant with the set validation criteria, i.e. R^2^ (Y) = 0.997, Q^2^(Y) = 0.828, a CV-ANOVA *P *value < 0.001 and a good permutation plot. From this model, 63 components with discriminative potential could be listed (Supplementary table S5), and within the IE group, three samples, collected at day 1, and twice on day 6 following the last seizure event, showed a higher signal for four specific untargeted discriminating metabolic components. This increase was less pronounced in the other samples, collected at day 14, 21, 28 and 76 following the last seizure event (Fig. 4). Further putative identification was pursued, but no components could be matched.

## Discussion

Despite the rapid increase in the use of metabolomics, standardized protocols for pretreatment of specific matrices like CSF are often lacking. Recently, Song et al. optimized CSF sample pretreatment for metabolomics, with a focus on the protein precipitation step^23^, leading to similar recommendations for solvent use in the final extraction protocol as compared to our work. Similarities include the use of methanol in combination with other protein precipitation solvents, and the use of water as the biggest fraction of the reconstitution solvent. Using said protocol, similar percentages of acceptable instrumental precision were found for the untargeted fingerprint, i.e. 78–98% vs. 85% in our study. However, this was accompanied by a lower absolute number of components, i.e. n = 406–702 in their method vs. n = 1256 in our study^23^. Our work also included determination of within-lab precision to assess reproducibility and intra-assay precision besides instrumental precision. To the best of our knowledge, this is the first report confirming the repeatable and reproducible detection of 102 known metabolites (with a broad physicochemical diversity) in CSF, combined with an untargeted fingerprint with a remarkable number of untargeted metabolic components (n = 1159–1480).

Within the targeted evaluation, 8 metabolites were detected in CSF for the very first time. The novelty of their detection in CSF was checked by a literature and Human Metabolome Database search (October 2023)^24^. All mentioned metabolites were described in other biofluids like blood, faeces, urine, saliva and/or bile, yet not previously in CSF. The metabolites reached good linearity and precision (Supplementary Table S6), and different metabolite classes were covered, including carboxylic acids and derivatives (i.e. *N*-acetyl-l-glutamic acid, *N*-acetyl-l-methionine and, ɣ-glutamylphenylalanine), an organic disulfide (i.e. dipropyl disulfide), an organooxygen compound (i.e. saccharic acid), a diazine (i.e. cytosine), an imidazopyrimidine (i.e. 7-methylguanine) and a bile acid (i.e. sodium taurocholate).

Optimization of the experimental design and sample preparation remains one of the key aspects to ensure standardization and reproducibility in CSF metabolomics studies^25^. The obtained validation characteristics (Supplementary Table S3) can guide the use of our extraction method in future metabolomics studies. The development of a methodology for the extraction and detection of lipids to complement the hereby described metabolomics methodology may be envisioned as well, or a dual polar metabolomics and lipidomics method, directly combining the two^26^. Ultimately, the CNS contains the second highest amount of lipids, preceded only by adipose tissue^27^.

The small sample size (n = 15) and lack of a healthy control group are two major limitations in the current CSF metabolomics study. CSF samples from healthy dogs were only available from laboratory Beagles, collected between 2013 and 2019, whereas the IE dogs included various client-owned breeds with sample collections within a 1 year time frame. These differences, especially the influence of breed^28^, would induce confounders hampering the interpretation of the results. Therefore, CSF samples from dogs with similar patient characteristics (Tables 2 and 3) and non-brain disease were included in the current control group, based on the availability of diagnostic left-overs. These samples were collected via atlanto-occipital (n = 12) or lumbar punction (n = 3). Routine CSF analysis was only conducted and evaluated in 11/15 samples, for which findings were within reference ranges. Future studies should aim to include CSF samples in the control group with normal routine CSF analysis available and collected only via atlanto-occipital punction, to reduce sample variability in the control group. Moreover, CSF samples from healthy dogs or dogs with structural epilepsy rather than non-brain disease would improve clinical interpretation of the obtained results.

Despite these limitations, significant alterations in the CSF metabolism of dogs with IE were identified compared to dogs with non-brain related disease, i.e. the control group. In the untargeted analysis, four samples within the IE group, collected at day 1, and twice on day 6 following the last seizure event, showed a higher signal for four of the untargeted discriminating metabolic components. This finding points towards an interesting future area of research regarding acute versus chronic metabolic changes.

**Figure 5:**
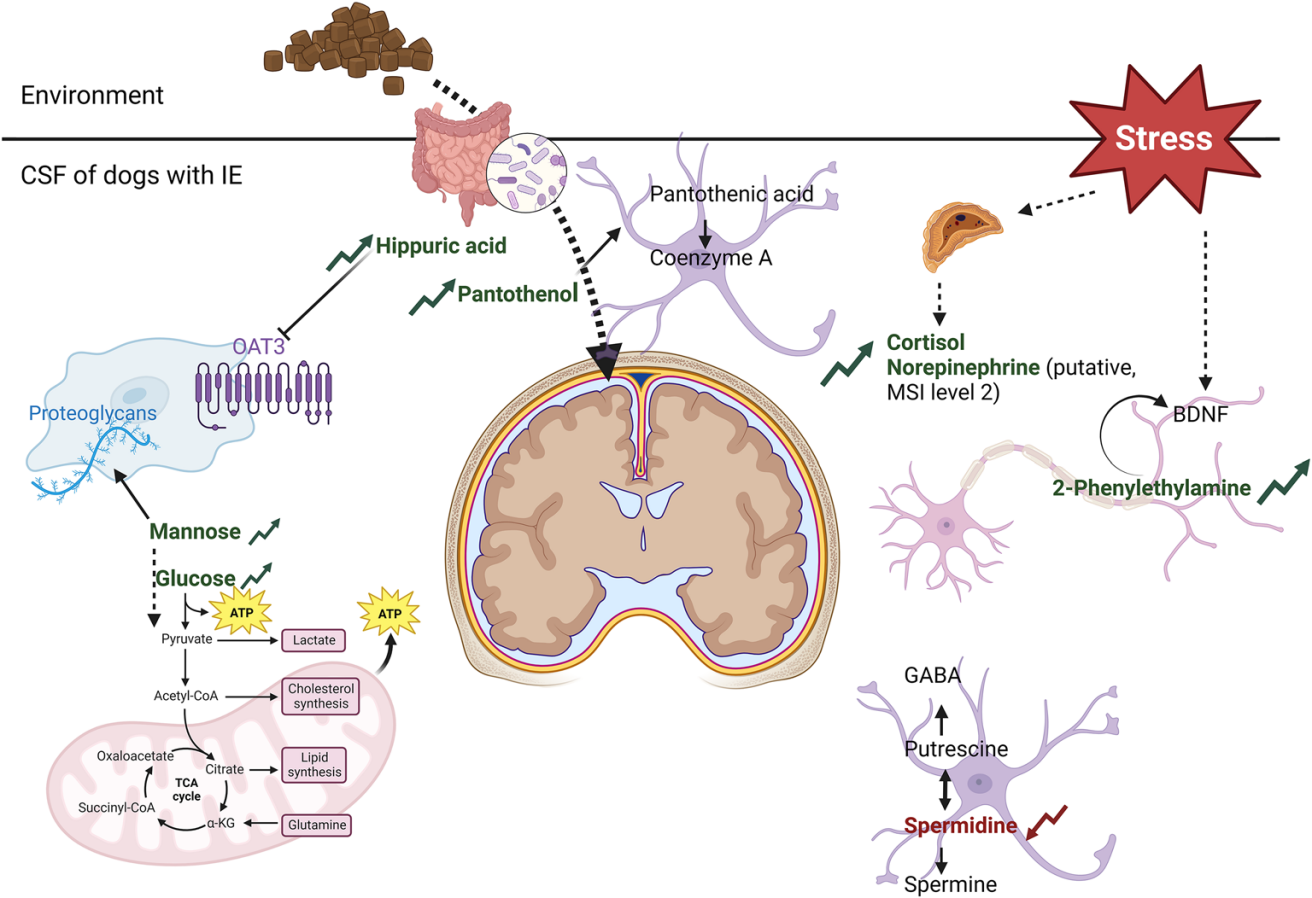
Illustration of the biological pathways involved in the CSF metabolic alterations found in dogs with IE compared to dogs with non-brain related disease. *BDNF* Brain derived neurotrophic factor, *OAT3* organic anion transporter 3.

In the targeted analysis, a significant alteration was found in metabolites involved in different metabolic pathways (Fig. 5). Firstly, energy metabolism was altered in dogs with IE, as glucose, mannose and creatinine were increased in the CSF of dogs with IE compared to the control group. Glucose is primordial in the brain for both energy generation and biosynthesis of lipids and proteins. However, the most efficient oxidative glucose metabolism, i.e. the citrate cycle, takes place in the mitochondria. Therefore, glucose transporters regulate its uptake in brain cells^29^. As a consequence, the higher glucose levels in CSF are believed to be related to reduced uptake by these glucose transporters. Moreover, the energy shortage theory states that an epileptogenic brain shows lower glucose transport and phosphorylation, combined with a higher energy demand, contributing to the generation of epileptic seizures^30^. Mannose, an epimer of glucose, is known as a less efficient cellular energy source, but of main importance for protein glycosylation^31^. Recently, the diversity and expression of glycosylated proteins in the mammalian brain was studied, whereby a specific brain glycosylation was seen, highly conserved between species (mouse and human) but clearly distinct from serum glycosylation in both species. Interestingly, around 20% of the observed glycan classes in the brain were high-mannose glycans. In brain tissue specifically, adhesion molecules and cell surface-recognition molecules are known to carry these high-mannose glycans^32^. Therefore, the observed increase in mannose could be related to an alteration in neural cell or glia adhesion and cell surface-recognition molecules, besides energy metabolism. Creatinine is the breakdown product of creatine. The latter is a nitrogenous guanidine compound playing a key role in cellular energy metabolism, especially in tissue with high energy demands, like the brain^33^. Degradation of creatine to creatinine occurs non-enzymatically on a daily basis in healthy individuals^34^. However, creatinine itself has been described as a pro-convulsive metabolite, for which transporter-mediated processes across the blood-CSF barrier are involved in cerebral clearance^35^. Our results demonstrate an increase in creatinine in the CSF of dogs with IE compared to dogs with non-brain related diseases, with no significant difference in creatine CSF levels, possibly indicating an alteration in clearance rather than metabolic turn-over.

Secondly, stress metabolism was altered as cortisol, and 2-phenylethylamine levels were increased in the CSF of dogs with IE compared to the control group. Cortisol is typically released by the hypothalamic pituitary adrenal (HPA) axis as a response to stress, regulated by numerous regulatory pathways^36^. Diurnal plasma cortisol variations occur in dogs, as in humans, and therefore lower concentrations are expected at 8 a.m. and 8 p.m., and higher concentrations at 4 p.m.^37^. In humans, it has been demonstrated that CSF cortisol follows the diurnal rhythm of plasma cortisol^38^. Therefore, it is important to consider the timing of CSF collection in our study, which was between 9 a.m. and 3 p.m. for all dogs except one control sample, which was collected at 7 p.m. Furthermore, samples were collected 1–76 days after the last seizure event, but no link was seen between cortisol levels and time between seizure event and sample collection (Supplementary Fig. S3). Moreover, no outliers were detected, indicating a minimal effect of timing on our results. The increased cortisol levels found in dogs with IE confirmed earlier findings from literature, whereby preclinical studies indicate a pro-epileptic role for cortisol^39^. Long-term exposure to cortisol moreover induces neuronal cell loss in the hippocampus of rats^40^, further contributing to hyperexcitability in epilepsy. Additionally, in dogs, like humans, the most reported common seizure precipitating factor is stress^41^, which is assumed to involve similar mechanisms. Moreover, 2-phenylethylamine was also increased in the CSF of dogs with IE. 2-Phenylethylamine is a brain amine with sympathomimetic effects, known to potentiate cortical neuron response to norepinephrine and affect the brain-derived neurotrophic factor (BDNF) signaling pathway in response to stress^42^. This could further intensify the stress-related signaling in the brain. Previously, Schmidt et al. did not observe significant differences in urinary 2-phenylethylamine levels comparing healthy to dogs with IE^43^. It could be hypothesized that CSF is more sensitive than urine to pick-up alterations in 2-phenylethylamine. Overall, our findings regarding stress metabolism are in accordance with the current state of the art, where evidence suggests a critical role for the HPA axis and stress in the pathophysiology of epilepsy^41^.

Thirdly, an alteration in the astrocytic polyamine and GABA metabolism, whereby putrescine is converted to either GABA or spermine through spermidine^44^, was observed. The CSF of dogs with IE showed a decrease in spermidine, but no alterations in GABA itself. Spermine however did not meet the preconceived area threshold for targeted metabolite integration and was therefore not included in the current CSF study. Recently, Kovacs et al. showed that inhibition of spermidine synthesis could prevent seizure generation in a rat model by increasing GABA production^44^. Based on our data, we hypothesize that a larger percentage of putrescine is needed for conversion to GABA in dogs with IE compared to dogs with non-brain related disease, resulting in lower conversion of putrescine to spermidine. Since all dogs with IE in our study received at least one type of antiseizure medication (ASM) that interacts with the GABA-receptor or neurotransmitter release^45^, this may have influenced the polyamine GABA metabolism. However, no clear link with any type of ASM and the metabolite levels was seen. Additional research including drug-naïve dogs and evaluation of spermine despite the low peak areas of this metabolite is to provide better insights in the functional alterations of this pathway.

Lastly, our metabolomics results suggest that not only endogenous (patho)physiology and metabolism plays a role, but that external exposures may be contributors to IE, as reflected by the detected metabolic changes related to feed intake, i.e. hippuric acid and pantothenol. Both were found to be increased in the CSF of dogs with IE. Hippuric acid is known as a human urine and plasma marker for consumption of phenolic compounds, like whole grains, fruits and plant-based products, which are also common ingredients used in commercial petfood. It is described as a metabolite on the crossroad of diet, gut bacterial metabolism, liver and kidney function^46^. A potential role in frailty and ageing processes has been discovered^46^, whereby lower urine and plasma hippuric acid levels were found in people with physical frailty, despite a general tendency to increase with ageing^46^. Furthermore, a positive association between plasma hippuric acid levels and Parkinson’s disease was shown^47^, whilst another study attributed neurotoxic properties to hippuric acid. The latter was related to the inhibition of the specific ‘Organic anion transporter 3′, resulting in reduced efflux of neurotransmitter metabolites from the brain^48^. Further research is warranted to better understand the role of hippuric acid metabolism in dogs with IE. Pantothenol is a xenobiotic precursor for pantothenic acid, approved as feed additive by EFSA^49^. It is known that its transformation to pantothenic acid is very efficient in CNS^50^. Indeed, the levels of pantothenic acid in all samples are much higher compared to pantothenol, with a pantothenol/pantothenic acid ratio of 0.03 ± 0.03 in the CSF of dogs with IE and 0.01 ± 0.01 in the CSF of control dogs. Our results only show a significant increase in pantothenol itself and not in pantothenic acid, nor in the pantothenol/pantothenic acid ratio. Pantothenic acid, however, is known to be deficient in multiple human neurodegenerative diseases, like Huntington’s or Alzheimer’s. Supplementation of pantothenol is moreover suggested as a possible treatment for these diseases^51^. The latter seems in contrast with our findings, but most pet dogs are fed complete and balanced commercial diets^52^, making nutritional deficiencies very unlikely. Moreover, polyphagia is a well-known adverse effect of the ASM used in the IE dogs^53^, resulting in an increased food intake. The latter could explain the increased levels of both feed-related metabolites, i.e. hippuric acid and pantothenol.

## Conclusion

The main strength and purpose of this study lies in the optimization of a novel CSF extraction method and the successful targeted and untargeted validation of the CSF metabolomics analysis method prior to its use in a clinical setting, increasing repeatability and reproducibility for a wide range of metabolites in canine CSF. To the best of our knowledge, it is the first study that assessed precision and linearity in depth for 102 targeted metabolites together with more than 1000 untargeted metabolite compounds, whereby most of the evaluated compounds met the set criteria for validation. Moreover, within the targeted validation, 8 metabolites were detected for the first time in CSF specifically. Furthermore, the use of the newly established and validated method in a clinical setting was demonstrated in a canine cohort of dogs with IE compared to dogs with non-brain related diseases. Alterations in energy, stress and astrocytic polyamine and GABA metabolism, together with an alteration in some feed-linked metabolites, as well as a distinct metabolic fingerprint, suggest differences in biologically relevant pathways and clearly demonstrate the potential of CSF metabolomics in epilepsy research, which is underexplored to date.

## Methods

### Analytical standards and reagents

Analytical and internal standards (of known endogenously occurring metabolites previously detected in other bio-fluids and/or CSF) used for UHPLC-HRMS are listed in Supplementary Table S7. Analytical standards were purchased individually from Sigma-Aldrich (St-Louis, Missouri, USA), ICN Biomedicals Inc. (Ohio, USA), TLC Pharmchem (Vaughan, Ontario, Canada) or Cambridge Isotope Laboratories Inc. (Tewksbury, Massachusetts, USA), to create in-house standard mixtures. Stock solutions were prepared at a concentration of 1 or 10 mg/ml with ultrapure water (0.055 µS/cm, delivered through a purified water system (VWR International, Belgium) or methanol. Stock solutions and the derived working solutions were stored at − 20 °C in amber glass vials. Solvents used for analysis were of LC–MS grade, obtained from VWR International (Belgium) or Fisher Scientific (USA).

### Biological samples

No prospective sampling was conducted for our research; instead, available left-over CSF samples were either pooled and used in the method optimization or used as such in our IE study cohort. CSF samples were left-overs from other, unrelated studies; EC 2011/130 and EC 2014/85^54^ collected from healthy laboratory Beagles (n = 25), combined with left-overs of diagnostic samples from canine patients at the Small Animal Department, Ghent University (n = 21) with a variety of indications for CSF collection. The CSF was collected between 2013 and 2022, by experienced veterinarians via atlanto-occipital or lumbar punction. CSF collection was performed between 9 a.m. and 3 p.m. for all dogs except one control sample, which was collected at 7 p.m. After collection, samples were stored in the fridge at 4 °C for a maximum of 24 h before transfer to the − 80 °C freezer^25^. From the 46 available CSF samples, 31 were pooled and used for the method optimization and validation, and 15 were used in the IE study cohort. For the latter, the CSF metabolome from eight dogs (6 male, 2 female), diagnosed with IE (TIER II level of confidence in accordance with the International Veterinary Epilepsy Taskforce consensus statement^55^), was compared to samples of seven dogs with non-brain related diseases (4 male, 3 female) in the control group. All samples from IE dogs and four control dogs were collected via atlanto-occipital punction, and three CSF samples from control dogs were collected via lumbar punction, as displayed in Table 3. Both groups included a variety of client-owned breeds and were collected between November 2021 and July 2022, to avoid the introduction of confounding factors like breed^28^, environmental exposures or storage time^56^. The mean age of the dogs was 5.7 ± 3.8 years at CSF collection, with no significant differences between IE (mean age: 4.8 ± 3.0 years) and control dogs (mean age: 6.7 ± 4.4 years). Samples from the dogs in the IE group were collected 1–76 days following an epileptic seizure event. All dogs in the IE group received antiseizure medication at the time of CSF collection, i.e. levetiracetam, phenobarbital, imepitoin or a combination of phenobarbital with either levetiracetam or potassium bromide. Population characteristics for the study cohort are detailed in Tables 2 and 3.

### UHPLC-HRMS analysis

UHPLC-HRMS analysis was performed according to De Paepe et al., using the same instrumental parameters^5^, as displayed in Supplementary Table S8. Instrument calibration was performed with ready-to-use calibration solutions according to the manufacturer’s guidelines (Thermo Fisher Scientific, USA). Operational conditions were evaluated by injecting a standard mixture of 262 targeted metabolites (1 ng/μL) (Supplementary Table S7) at the beginning of every sequence, followed by sample analysis in randomized order. When analyzing biological samples (see “Study in dogs with idiopathic epilepsy”), QC samples were prepared from a pool of all biological samples (n = 15). QCs were injected at the beginning of the analytical sequence for conditioning of the system and in between the analyzed biological samples (two QCs following every ten samples), together with one solvent blank (ACN) to allow for correction of instrumental drift.

### CSF analysis method optimization and validation

#### Optimization of the CSF extraction

Generic extraction of CSF metabolites was optimized by means of a design of experiments using JMP 15 software (SAS, UK). Firstly, a literature search was performed to select the relevant factors to include in our design^18,57–64^. Secondly, an FFD was established with 19 experiments and 3 center points to assess the effect of four factors: starting volume (100 µl or 200 µl), type of solvent (50% ACN in water (A) or ACN/methanol/acetone (1/1/1) (B)), centrifugation time (5 min or 10 min) and the usage (yes/no) of a PVDF membrane filter (13 mm diameter, 0.22 µm pore size, Merck, Ireland). The effect of each factor was evaluated based on the metabolome coverage (i.e. the total number of detected metabolic components in each sample) and individual peak areas of all Tier 1 identified targeted metabolites (n = 104). Factors with an effect on the metabolome coverage (*P *value < 0.05) or at least 25% of the evaluated targeted metabolites were retained for further optimization. The latter included a simple lattice MD and RSM, for which a selection of metabolites was evaluated more in detail. The MD included 10 experiments to assess the optimal ratio between ACN, methanol and acetone at 3 levels (33%, 66% and 100%), and UPW, methanol and acetone (33%, 66%, 100%). Finally, an RSM with 16 experiments and 2 center points was applied to further optimize the retained factors (i.e. starting volume, centrifugation time and speed) using a custom design.

#### Final CSF extraction protocol

In the final extraction protocol, 30 µl internal standard mixture (25 ng/µl alanine-d3 and dopamine-d4) was added to 350 µl of CSF. Next, 1050 µl of 30% UPW, 10% acetone and 60% methanol, was added and vortexed for 30 s, followed by protein precipitation during 60 min at 4 °C. After precipitation, the solution was centrifuged for 15 min at 5000×*g* at 4 °C. The supernatant was collected and evaporated to dryness under a stream of nitrogen with a Turbovap LV (Caliper Life sciences, USA). The remaining fraction was then resolved in 300 µl of UPW. After a short vortex, the extract was filtered using a PVDF membrane filter (13 mm diameter, 0.22 µm pore size, Merck, Ireland) and transferred to a glass HPLC-vial with insert.

#### Validation

The method’s analytical performance was assessed in accordance with the guidelines of Naz et al.^65^, both in a targeted and untargeted fashion. Assessment of linearity was based on the determination coefficient (R^2^) of a 9-point calibration curve established by diluting a CSF extract with UPW (1, 2, 5, 10, 20, 50, 100, 200, and 500 times), performed in triplicate. Calculations regarding linearity were performed for those targeted metabolites detected (n = 102) and untargeted metabolic components (n = 1159) recovered in all dilution series samples. Reproducibility and repeatability were assessed by calculating the instrumental, intra-assay and within-lab variability. This was based on the CV for those targeted metabolites detected (n = 102) and untargeted metabolic components (n = 1480) recovered in all precision series samples. Instrumental precision was assessed by repeatedly injecting the same QC sample, i.e. a pool from all extracts, ten times. For the intra-assay precision, multiple QCs (n = 8) were extracted under identical experimental conditions, whereas within-lab variability was evaluated by extracting multiple QCs (n = 16) on two different days, by two different analysts.

### Data processing and statistical analysis

For targeted metabolites, peak areas were obtained by manual integration using Xcalibur™ 4.1 (Thermo Fisher Scientific, USA). Only endogenous metabolites with a signal to noise ratio of at least 10 in 90% of the samples and a minimal area of 100,000 count-sec were retained. Identification was achieved based on accurate mass (*m/z*-value, considering both the molecular ion and C_13_-isotope) and retention time relative to that of an external standard (level 1 identification according to the Metabolomics Standards Initiative (MSI))^66^. Further data processing was executed using Excel (Microsoft, USA) and R (R Core Team (2021)). Significant differences, linearity and precision were assessed based on *P*-values, R^2^ and CV, respectively. *P* values < 0.05 were considered significant. The linearity was considered excellent for metabolites with an R^2^ > 0.99 or acceptable for R^2^ > 0.90. The cut-offs indicating an acceptable or good precision were determined at < 20% (when operating close to the limit of detection) or CV < 15%, respectively^65^.

Untargeted data preprocessing was performed with Compound Discoverer (CD) 3.3 (Thermo Fisher Scientific, USA), combining positive and negative ionization. Detected components were characterized by *m/z*-value (peak intensity threshold 500,000 a.u., mass tolerance 5 ppm), retention time (RT; maximum RT-shift 0.4 min) and peak intensity (minimal signal to noise ratio 10). Based on good clustering of the QC samples, no further normalization, transformation or scaling was applied for the untargeted data in the optimization and validation. *P* values and R^2^ were defined in an identical manner as described for the targeted approach. The cut-offs indicating an acceptable precision were determined at CV < 30%^65^.

### Study in dogs with idiopathic epilepsy

The novel CSF metabolomics method was applied in a clinical context, i.e. to study the metabolic differences in IE vs. non-brain disease as the control group in an adult client-owned dog cohort. Descriptive statistics include mean ± standard deviation whenever relevant.

Targeted metabolites with a CV < 30% for instrumental precision were further processed for statistical analysis in R^67^ (n = 101, Supplementary Table S9). Metabolites following a normal distribution were evaluated with a Welch two sample T-test, and for non-normally distributed metabolites a Wilcoxon rank-sum test was used. The *P* value and log2 fold change were calculated for each targeted metabolite. *P* values < 0.05 were considered significant. If biologically relevant, a ratio between metabolites or Pearson correlation was calculated in Excel to assess the relationship between specific metabolites.

Data modelling for the untargeted metabolic components was performed in Simca 17.1 (Umetrics AB, Sweden) following preprocessing in CD 3.1 (Thermo Fisher Scientific, USA). Detected components were characterized similarly as previously described in "Data processing and statistical analysis", except for the maximum RT-shift, which was set at 0.2 min. Firstly, principal component analysis (PCA-X) was performed to explore the data, check for potential instrumental drift, and identify the most optimal approach for data normalization, transformation and scaling based on clustering of QC-samples. Data was normalized using internal QC (iQC)-normalization, log transformed, and Pareto scaled. Following this, an OPLS-DA model was constructed to elucidate potential metabolic differences between the IE and the control group. The model characteristics R^2^(X) and R^2^(Y) for fit, Q^2^(Y) for predictivity, and cross-validated analysis of variance (CV-ANOVA, *P* value < 0.05) and permutation testing (n = 100) were assessed to evaluate model validity. Listing of discriminative metabolic components was done based on a variable importance in projection (VIP) score > 1.5, Jack-Knifed confidence interval not including 0, and an excentric position in the S-plot (|*P*-corr|> 0.6). The retained compounds were putatively identified whenever possible, by matching measured *m/z* values (< 5 ppm difference) to theoretical *m/z* values in the Chemspider or in-house database (level 2 identification according to MSI)^66^.

### Ethical declaration

This research was in compliance with European legislation on animal experimentation (EU directive 2010/63/EU) and ARRIVE guidelines. Regarding the samples from laboratory Beagles, these were collected in the past under the ethical approval of the ethical committee of the faculties of Veterinary Medicine and Bioscience Engineering (EC 2011/130 and EC 2014/85), for which the respective studies were finished, leaving CSF aliquots left-over. Regarding the left-over samples from the Small Animal Department, formal ethical approval was waived by the ethical committee, based on Belgian and European legislation (EU directive 2010/63/EU), as no additional samples were collected for research purpose. Informed consent was obtained from the owners to use left-over samples for research purposes. In adherence with the European privacy regulations (EU Regulation 2016/679), the left-over samples cannot be traced back to the dogs or owners based on the published information.

## Supplementary Information

Supplementary Information.

## Data Availability

Data on the samples and a summary of the results are provided within the manuscript. Data on analytical standards, instrumental settings and detailed results per metabolite are provided within the supplementary information. The datasets generated and analyzed are available from the corresponding author upon reasonable request. The raw data will be made available to researchers for the purpose of replication and further analysis.
