# Correction: Towards a better understanding of idiopathic epilepsy through metabolic fingerprinting of cerebrospinal fluid in dogs

**DOI:** 10.1038/s41598-026-48734-6

**Published:** 2026-04-28

**Authors:** Fien Verdoodt, Sofie F. M. Bhatti, Karla Kragic, Luc Van Ham, Lynn Vanhaecke, Myriam Hesta, Lieselot Y. Hemeryck

**Affiliations:** 1 Equine and Companion Animal Nutrition, Department of Morphology, Imaging, Orthopedics, Rehabilitation and Nutrition, Faculty of Veterinary Medicine, Ghent University, Salisburylaan 133, 9820 Merelbeke, Belgium; 2 SmallAnimal Department, Faculty of Veterinary Medicine, Ghent University, Salisburylaan 133, 9820 Merelbeke, Belgium; 3 Laboratory of Integrative Metabolomics, Department of Translational Physiology, Infectiology and Public Health, Faculty of Veterinary Medicine, Ghent University, Salisburylaan 133, 9820 Merelbeke, Belgium

Correction to: *Scientific Reports* 10.1038/s41598-024-64777-z, published online 26 June 2024

The original version of this Article contained errors:

During Figure creation, figure 4 was incorrectly created using a different selection of untargeted discriminating components. The incorrect Fig. 4 and its accompanying legend appear below.

**Fig. 4:**
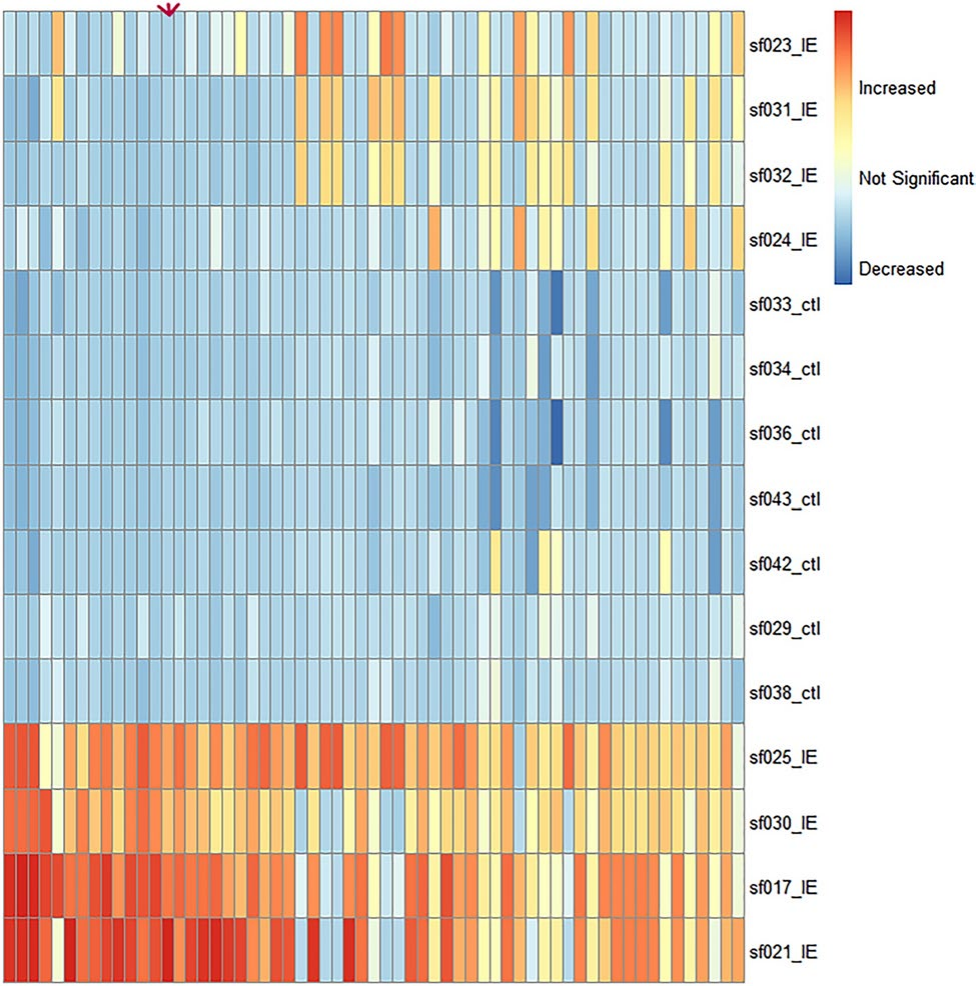
Heatmap displaying all untargeted discriminating components (n = 61) obtained from the IE group vs. ctl. Each column represents a specific untargeted component, for which the column indicated with an arrow was putatively identified (MSI level 2) as norepinephrine. Each row indicates one CSF sample, for which the group is indicated on the heatmap (ctl vs. IE). This figure was created by the authors using the R pheatmap package (Kolde R (2019). _pheatmap: Pretty Heatmaps_. R package version 1.0.12, https://CRAN.R-project.org/package=pheatmap).

Additionally, the dataset including the components with discriminative potential, used to create Figure 4, in the supplementary information file has been included. The original Supplementary Information file is provided here.

The original Article has now been corrected.

## Supplementary Information

Supplementary Information.

