# Supplementary material for: Towards a better understanding of idiopathic epilepsy through metabolic fingerprinting of cerebrospinal fluid in dogs
Source: Sci Rep. 2024 Jun 26;14:14750. doi: 10.1038/s41598-024-64777-z (PMC11208596; doi:10.1038/s41598-024-64777-z)

# Supplementary information for:

# Towards a better understanding of idiopathic epilepsy through metabolic fingerprinting of cerebrospinal fluid in dogs

Fien Verdoodt [a,b,c], Sofie F.M. Bhatti [b] , Karla Kragic [a], Luc Van Ham [b], Lynn Vanhaecke [c],

Myriam Hesta [a,1], Lieselot Y. Hemeryck [c,1,*]

**Author affiliations**:

[a] *Equine and Companion Animal Nutrition, Department of Morphology, Imaging, Orthopedics, Rehabilitation and Nutrition, Faculty of Veterinary Medicine, Ghent University, Salisburylaan 133, 9820 Merelbeke, Belgium – Fien.Verdoodt@Ugent.be, Myriam.Hesta@Ugent.be*
[b] *Small Animal Department, Faculty of Veterinary Medicine, Ghent University, Salisburylaan 133, 9820 Merelbeke, Belgium – Sofie.Bhatti@Ugent.be, Luc.VanHam@Ugent.be*
[c] *Laboratory of Integrative Metabolomics, Department of Translational Physiology, Infectiology and Public Health, Faculty of Veterinary Medicine, Ghent University, Salisburylaan 133, 9820 Merelbeke Belgium – LieselotY.Hemeryck@Ugent.be, Lynn.Vanhaecke@Ugent.be*

*** Corresponding author: LieselotY.Hemeryck@Ugent.be**

[1]These authors contributed equally to this work

**Tables S1: Table showing the obtained *P*-value for each targeted metabolite as evaluated in the FFD.**

| ***P*-values FFD** | **Startvolume (100 µl / 200 µl)** | **Filter (yes / no)** | **Centrifuge time (5 min / 10 min)** | **Solvent (A / B)** |
|---|---|---|---|---|
| Total untargeted compounds detected | 1.0E-05 | 4.5E-03 | > 0.05 | > 0.05 |
| Targeted metabolites | | | | |
| 1,3-propanediol | 1.1E-04 | > 0.05 | > 0.05 | > 0.05 |
| 1-methyl-L-histidine | 1.7E-02 | > 0.05 | > 0.05 | > 0.05 |
| 2,6-diaminopimelic acid | > 0.05 | > 0.05 | > 0.05 | > 0.05 |
| 2-hydroxyhexanoic acid | 0.0E+00 | > 0.05 | 1.4E-03 | > 0.05 |
| 2-hydroxyisocaproic acid | 0.0E+00 | > 0.05 | 2.2E-04 | 4.1E-02 |
| 2-piperidinone | 0.0E+00 | > 0.05 | 1.6E-04 | 1.3E-04 |
| 3,4-dihydroxyphenylpropionic acid | 1.1E-04 | > 0.05 | > 0.05 | > 0.05 |
| 3-hydroxybutyric acid | 0.0E+00 | > 0.05 | 1.1E-02 | > 0.05 |
| 3-indoleacetic acid | 1.0E-05 | > 0.05 | > 0.05 | 3.0E-05 |
| 3-methylbutyryl-L-carnitine | 2.0E-05 | > 0.05 | > 0.05 | > 0.05 |
| 3-phenyl-1-propanol | 3.7E-03 | > 0.05 | > 0.05 | > 0.05 |
| 3-phenylpropionic acid | 2.7E-02 | > 0.05 | > 0.05 | 3.9E-02 |
| 4-guanidinobutyric acid | 1.1E-02 | > 0.05 | 4.6E-02 | > 0.05 |
| 4-methyl-2-oxovaleric acid | 0.0E+00 | > 0.05 | 4.1E-04 | 6.1E-04 |
| 5-hydroxyindole-3-acetic acid | 0.0E+00 | 1.5E-02 | > 0.05 | 1.3E-04 |
| 6-deoxyhexose | 3.9E-02 | > 0.05 | > 0.05 | 1.1E-03 |
| 7-methylguanine | 0.0E+00 | 2.4E-02 | 9.0E-05 | 3.0E-05 |
| acetophenone | 0.0E+00 | 5.9E-03 | 3.2E-04 | 8.1E-03 |
| acetylcholine | 1.0E-05 | > 0.05 | > 0.05 | 1.4E-03 |
| adenine | 0.0E+00 | > 0.05 | 7.9E-04 | 0.0E+00 |
| adenosine-5-monophosphate | 3.8E-02 | > 0.05 | > 0.05 | > 0.05 |
| adipoylcarnitine | 0.0E+00 | > 0.05 | > 0.05 | > 0.05 |
| allantoin | 3.4E-02 | > 0.05 | 3.0E-02 | > 0.05 |
| azelaic acid | > 0.05 | 2.5E-03 | > 0.05 | 2.6E-02 |
| butyrylcarnitine | 0.0E+00 | > 0.05 | > 0.05 | > 0.05 |
| CDCA | 3.0E-05 | > 0.05 | > 0.05 | > 0.05 |
| cholic acid | 1.0E-05 | > 0.05 | > 0.05 | > 0.05 |
| cortisol | > 0.05 | > 0.05 | > 0.05 | > 0.05 |
| creatine | 0.0E+00 | > 0.05 | 2.2E-03 | > 0.05 |
| creatinine | 0.0E+00 | > 0.05 | 4.1E-03 | > 0.05 |
| cyclohexylamine | 2.0E-05 | > 0.05 | > 0.05 | 0.0E+00 |
| cytosine | 0.0E+00 | > 0.05 | 7.7E-03 | > 0.05 |
| fructose/galactose | 0.0E+00 | > 0.05 | > 0.05 | > 0.05 |
| gluconic acid | 6.4E-03 | > 0.05 | 4.2E-02 | > 0.05 |
| glucose | 0.0E+00 | > 0.05 | 2.1E-04 | > 0.05 |
| dipropyl disulfide | 0.0E+00 | 1.2E-02 | 2.0E-05 | > 0.05 |
| maltose/sucrose | | | | |

| | | | | |
|---|---|---|---|---|
| dopamine | 0.0E+00 | > 0.05 | 2.7E-02 | 1.4E-02 |
| pantothenic acid | 0.0E+00 | > 0.05 | 8.0E-05 | > 0.05 |
| trehalose | 0.0E+00 | > 0.05 | 6.1E-03 | 1.4E-03 |
| GABA | 0.0E+00 | > 0.05 | 1.1E-03 | > 0.05 |
| gamma-glutamylphenylalanine | 0.0E+00 | > 0.05 | 4.6E-02 | > 0.05 |
| glyceric acid | 0.0E+00 | > 0.05 | > 0.05 | > 0.05 |
| glycerol | 2.5E-03 | > 0.05 | > 0.05 | > 0.05 |
| glycoursodeoxycholic acid (GUDCA) | 1.0E-05 | > 0.05 | > 0.05 | > 0.05 |
| hexadecamide | > 0.05 | 1.0E-05 | > 0.05 | > 0.05 |
| hippuric acid | 0.0E+00 | > 0.05 | 2.5E-03 | > 0.05 |
| homocysteine | 0.0E+00 | 3.4E-03 | 3.8E-04 | 2.6E-04 |
| hydroquinone | 0.0E+00 | 4.0E-05 | 3.2E-04 | > 0.05 |
| hypoxanthine | 0.0E+00 | > 0.05 | 1.1E-03 | 4.3E-04 |
| isobutyrylcarnitine | 0.0E+00 | > 0.05 | > 0.05 | > 0.05 |
| kynurenic acid | 0.0E+00 | > 0.05 | 7.1E-03 | > 0.05 |
| kynurenine | 0.0E+00 | > 0.05 | 7.4E-04 | 1.8E-02 |
| lactic acid | 0.0E+00 | 2.4E-02 | 1.1E-04 | 5.5E-02 |
| arginine | 0.0E+00 | > 0.05 | 6.9E-03 | > 0.05 |
| citrulline | 2.1E-04 | 2.9E-02 | 1.5E-02 | > 0.05 |
| glutamine | 0.0E+00 | > 0.05 | 6.0E-03 | > 0.05 |
| linoleamide | > 0.05 | 2.0E-05 | > 0.05 | 9.0E-03 |
| isoleucine | 0.0E+00 | > 0.05 | 2.2E-04 | > 0.05 |
| leucine | 0.0E+00 | > 0.05 | 1.0E-05 | > 0.05 |
| methionine | 0.0E+00 | 4.3E-02 | 3.0E-05 | > 0.05 |
| phenylalanine | 0.0E+00 | > 0.05 | 7.3E-04 | > 0.05 |
| proline | 0.0E+00 | > 0.05 | 2.4E-02 | > 0.05 |
| pyroglutamic acid | > 0.05 | > 0.05 | > 0.05 | > 0.05 |
| tyrosine | 0.0E+00 | 3.4E-02 | 1.3E-04 | > 0.05 |
| malonylcarnitine | 0.0E+00 | > 0.05 | > 0.05 | > 0.05 |
| N,N-dimethylarginine | 6.0E-05 | > 0.05 | 1.5E-02 | 8.0E-03 |
| N6-acetyl-L-lysine | 0.0E+00 | > 0.05 | > 0.05 | > 0.05 |
| N-acetylasparagine | > 0.05 | > 0.05 | > 0.05 | 5.3E-03 |
| N-acetylglutamic acid | > 0.05 | > 0.05 | 3.4E-02 | 3.5E-02 |
| N-acetylglycine | 6.7E-03 | 1.7E-02 | 2.3E-02 | 6.4E-03 |
| N-acetyl-L-methionine | 0.0E+00 | > 0.05 | 4.4E-03 | 2.3E-04 |
| N-acetyl-L-phenylalanine | 2.0E-05 | > 0.05 | > 0.05 | 2.0E-05 |
| N-acetyltryptophan | 3.0E-02 | 6.6E-03 | > 0.05 | 0.0E+00 |
| N-acetyltyrosine | 0.0E+00 | > 0.05 | > 0.05 | 1.4E-02 |
| nicotinamide | 0.0E+00 | > 0.05 | 3.7E-04 | > 0.05 |
| nicotinic acid | 0.0E+00 | > 0.05 | 5.9E-04 | > 0.05 |
| N-isovalerylglycine | 0.0E+00 | > 0.05 | 8.8E-04 | 4.4E-03 |
| octanoyl-L-carnitine | 3.2E-02 | > 0.05 | 2.8E-02 | 1.0E-05 |
| oleamide | > 0.05 | 2.5E-04 | > 0.05 | > 0.05 |
| opthalmic acid | 5.4E-03 | > 0.05 | > 0.05 | > 0.05 |
| O-succinylcarnitine | 6.4E-03 | > 0.05 | > 0.05 | > 0.05 |

| | | | | |
|---|---|---|---|---|
| oxoglutaric acid | > 0.05 | > 0.05 | 4.7E-02 | 4.6E-04 |
| pantothenol | > 0.05 | > 0.05 | > 0.05 | > 0.05 |
| phenylacetic acid | 2.0E-05 | > 0.05 | > 0.05 | > 0.05 |
| phenylAc-GLN-OH | 9.4E-04 | > 0.05 | > 0.05 | > 0.05 |
| phosphocholine | > 0.05 | > 0.05 | > 0.05 | > 0.05 |
| pimelic acid | 0.03479 ^ | 1.3E-03 | > 0.05 | > 0.05 |
| pipecolic acid | 0.0E+00 | 2.4E-02 | 2.6E-04 | 3.2E-03 |
| putrescine | 1.7E-04 | > 0.05 | > 0.05 | 3.1E-04 |
| pyrrole-2-carboxylic acid | 0.0E+00 | 7.6E-02 | > 0.05 | > 0.05 |
| pyruvic acid | 0.0E+00 | > 0.05 | > 0.05 | 1.0E-05 |
| rac-glycerol-1-phosphate sodium salt hydrate | 2.7E-02 | 3.4E-02 | > 0.05 | > 0.05 |
| saccharic acid | 1.9E-04 | > 0.05 | > 0.05 | 5.7E-04 |
| S-adenosylhomocystein | 2.5E-04 | > 0.05 | > 0.05 | > 0.05 |
| sebacic acid | 3.0E-05 | 2.8E-03 | 8.5E-03 | 6.5E-04 |
| sodium glycodeoxycholate (GDCA) | 2.0E-05 | > 0.05 | > 0.05 | > 0.05 |
| sodium taurocholate (TCA) | 0.0E+00 | 2.0E-02 | > 0.05 | > 0.05 |
| spermidine | 3.5E-03 | > 0.05 | > 0.05 | > 0.05 |
| succinic acid | 0.0E+00 | 4.2E-04 | 1.9E-03 | 2.1E-04 |
| tryptophan | 0.0E+00 | > 0.05 | 5.4E-03 | > 0.05 |
| uracil | 0.0E+00 | > 0.05 | 2.7E-02 | 1.4E-02 |
| urocanic acid | > 0.05 | 1.6E-02 | > 0.05 | 5.4E-02 |
| valerylcarnitine | 0.0E+00 | > 0.05 | > 0.05 | > 0.05 |

**Table S2: Table showing the obtained *P*-values for the selection of targeted metabolites as evaluated in the RSM.**

| ***P*-values RSM** | **starting V (100 - 400 µl)** | **centrifuge t (5 - 15 min)** | **centrifuge v (5000 xg - 15 000 xg)** |
|---|---|---|---|
| targeted metabolome coverage | 0.0211 | 0.0946 | 0.0930 |
| untargeted metabolome coverage | 0.0007 | 0.3170 | 0.9852 |
| butyric acid | 0.0265 | 0.4524 | 0.5338 |
| pyrrole-2-carboxylic acid | 0.0001 | 0.3808 | 0.7946 |
| indole-3-acetic acid | 0.0002 | 0.9174 | 0.5982 |
| L-glutamine | 0.0002 | 0.4514 | 0.9674 |
| glutamic acid | 0.0175 | 0.7814 | 0.9776 |
| glucose | 0.0001 | 0.4551 | 0.5581 |
| GABA | 0.0003 | 0.4183 | 0.7844 |
| tyrosine | 0.0001 | 0.2482 | 0.7170 |
| dopamine | 0.0004 | 0.1470 | 0.8176 |
| cortisol | 0.0004 | 0.8177 | 0.4560 |
| kynurenine | 0.0001 | 0.9531 | 0.3559 |
| tryptophan | 0.0001 | 0.9668 | 0.6207 |
| kynurenic acid | 0.0001 | 0.6226 | 0.4096 |
| **Effect** | *positive towards 400 µl, plateau at 350 µl* | *positive towards 15 min* | *positive towards 5000 xg* |

**Table S3: Table showing the results of our validation for each of the evaluated targeted metabolites.**

| Targeted metabolite | Chemical Formula | Ion Adduct | m/z-value | Retention Time (min) | $R^2$ | Dilution range | CV IP (%) |
|---|---|---|---|---|---|---|---|
| 1,3-propanediol | $C_3H_8O_2$ | [M+H]+ | 77.05971 | 1.33 | 0.990 | 0.002-1 | 21.79 |
| 2-hydroxyhexanoic acid | $C_6H_{12}O_3$ | [M-H]- | 131.0695 | 9.4 | 0.999 | 0.002-1 | 11.48 |
| 2-hydroxyisocaproic acid | $C_6H_{12}O_3$ | [M+H]+ | 131.06995 | 9.16 | 0.999 | 0.002-1 | 8.01 |
| 2-hydroxy 3-methylvalerate | $C_6H_{12}O_3$ | [M-H]- | 131.06950 | 9.03 | 0.999 | 0.002-1 | 9.17 |
| 2-methylbutyryl-L-carnitine | $C_{12}H_{23}NO_4$ | [M+H]+ | 246.16998 | 8.55 | 0.987 | 0.002-1 | 8.42 |
| 2-phenylethylamine | $C_8H_{11}N$ | [M+H]+ | 122.09643 | 6.59 | 0.096 | 0.02-1 | 14.08 |
| 2-piperidinone | $C_5H_9NO$ | [M+H]+ | 100.07569 | 5 | 0.991 | 0.002-1 | 4.09 |
| 3-indoleacetic acid | $C_{10}H_9NO_2$ | [M+H]+ | 176.07061 | 8.84 | 0.998 | 0.002-1 | 7.61 |
| 3-methoxytyramine hydroxychloride | $C_9H_{13}NO_2$ | [M+H]+ | 168.10191 | 4.4 | 1.000 | 0.002-1 | 12.00 |
| 3-methylbutyryl/isovalaryl-L-carnitine | $C_{12}H_{23}NO_4$ | [M+H]+ | 246.16998 | 8.21 | 0.954 | 0.002-1 | 9.38 |
| 4-guanidinobutyric acid | $C_5H_{11}N_3O_2$ | [M+H]+ | 146.09220 | 1.44 | 0.987 | 0.002-1 | 7.60 |
| 4-hydroxyphenylpyruvic acid | $C_9H_8O_4$ | [M-H]- | 179.03498 | 4.55 | 0.999 | 0.002-1 | 6.02 |
| 4-methyl-2-oxovaleric acid | $C_6H_{10}O_3$ | [M-H]- | 129.05432 | 8.41 | 1.000 | 0.002-1 | 6.72 |
| 5-hydroxyindole-3-acetic acid | $C_{10}H_9NO_3$ | [M+H]+ | 192.06552 | 8.04 | 0.996 | 0.002-1 | 7.27 |
| 6-acetyl-L-lysine | $C_8H_{16}N_2O_3$ | [M+H]+ | 189.12337 | 1.47 | 0.990 | 0.002-1 | 9.20 |
| 7-methylguanine | $C_6H_7N_5O$ | [M+H]+ | 166.07234 | 1.62 | 0.981 | 0.002-1 | 4.26 |
| acetophenone | $C_8H_8O$ | [M+H]+ | 121.06479 | 11.28 | 0.709 | 0.002-1 | 6.97 |
| acetylcholine | $C_7H_{16}ClNO_2$ | [M+H]+ | 146.11756 | 1.1 | 0.995 | 0.01-1 | 9.05 |
| adenine | $C_5H_5N_5$ | [M+H]+ | 136.06177 | 1.33 | 0.925 | 0.002-1 | 4.40 |
| adenosine-5-monophosphate | $C_{10}H_{14}N_5O_7P$ | [M+H]+ | 348.07036 | 1.47 | 0.985 | 0.002-1 | 28.53 |
| adipoylcarnitine C16 | $C_{13}H_{23}NO_6$ | [M+H]+ | 290.15981 | 6.16 | 0.991 | 0.002-1 | 13.11 |
| alfa-hydroxyisobutyric acid | $C_4H_8O_3$ | [M+H]+ | 105.05462 | 2.5 | 0.997 | 0.002-1 | 23.37 |
| allantoin | $C_4H_6N_4O_3$ | [M+H]+ | 159.05127 | 1 | 0.899 | 0.002-0.1 | 20.63 |
| anandamide (18:1) | $C_{20}H_{39}NO_2$ | [M+H]+ | 326.30536 | 15.04 | 0.850 | 0.002-1 | 14.20 |
| azelaic acid | $C_9H_{16}O_4$ | [M-H]- | 187.09758 | 8.83 | 0.994 | 0.002-1 | 11.82 |
| B-hydroxyisovaleric acid | $C_5H_{10}O_3$ | [M-H]- | 117.05572 | 3.9 | 0.998 | 0.002-1 | 23.04 |
| butyrylcarnitine | $C_{11}H_{21}NO_4$ | [M+H]+ | 232.15433 | 6.58 | 0.999 | 0.002-1 | 7.67 |

| | | | | | | | |
|---|---|---|---|---|---|---|---|
| chenodeoxycholic acid (CDCA) | $C_{24}H_{40}O_4$ | [M-H]- | 391.28534 | 12.9 | 0.998 | 0.002-1 | 7.25 |
| cholic acid | $C_{24}H_{40}O_5$ | [M-H]- | 407.2803 | 11.99 | 0.978 | 0.002-1 | 15.18 |
| choline | $C_5H_{14}NO$ | [M+H]+ | 104.10699 | 0.83 | 0.966 | 0.002-1 | 4.72 |
| corticosterone | $C_{21}H_{30}O_4$ | [M+H]+ | 347.22170 | 11.54 | 0.999 | 0.002-1 | 7.86 |
| cortisol | $C_{21}H_{30}O_5$ | [M+H]+ | 363.21660 | 11.17 | 0.984 | 0.002-1 | 7.57 |
| creatine | $C_4H_9N_3O_2$ | [M+H]+ | 132.0768 | 0.94 | 0.907 | 0.002-1 | 5.51 |
| creatinine | $C_4H_7N_3O$ | [M+H]+ | 114.0661 | 0.89 | 0.964 | 0.002-1 | 5.35 |
| cytidine | $C_9H_{13}N_3O_5$ | [M+H]+ | 244.0928 | 1.32 | 0.964 | 0.002-1 | 7.69 |
| cytosine | $C_4H_5N_3O$ | [M+H]+ | 112.05054 | 1.5 | 0.982 | 0.002-1 | 5.81 |
| Deoxycholic acid (DCA) | $C_{24}H_{40}O_4$ | [M-H]- | 391.28534 | 13.05 | 0.998 | 0.002-1 | 7.25 |
| dipropyl disulfide | $C_6H_{14}S_2$ | [M+H]+ | 151.06097 | 1.5 | 0.989 | 0.002-1 | 11.05 |
| dodecanedioic acid | $C_{12}H_{22}O_4$ | [M-H]- | 229.14453 | 9.44 | 0.987 | 0.002-1 | 3.38 |
| pantothenic acid | $C_9H_{17}NO_5$ | [M+H]+ | 220.11795 | 6.5 | 0.999 | 0.002-1 | 4.10 |
| GABA | $C_4H_9NO_2$ | [M+H]+ | 104.07061 | 0.92 | 0.972 | 0.002-1 | 10.33 |
| gamma-glutamylphenylalanine | $C_{14}H_{18}N_2O_5$ | [M+H]+ | 295.12884 | 8.03 | 1.000 | 0.002-1 | 8.03 |
| glucose | $C_6H_{12}O_6$ | [M+Na]+ | 203.05261 | 0.91 | 0.917 | 0.002-1 | 3.41 |
| glyceric acid | $C_3H_6O_4$ | [M-H]- | 105.01779 | 1.09 | 0.999 | 0.002-1 | 4.53 |
| guanine | $C_5H_5N_5O$ | [M+H]+ | 152.05669 | 1.36 | 0.899 | 0.02-1 | 30.80 |
| hexadecamide | $C_{16}H_{33}NO$ | [M+H]+ | 256.26349 | 15.18 | 0.647 | 0.002-0.1 | 11.04 |
| hippuric acid | $C_9H_9NO_3$ | [M+H]+ | 180.06529 | 8.45 | 0.997 | 0.002-1 | 6.27 |
| homocysteine | $C_4H_9NO_2S$ | [M+H]+ | 136.04268 | 1.13 | 0.966 | 0.002-1 | 25.39 |
| hypoxanthine | $C_5H_4N_4O$ | [M+H]+ | 137.04579 | 1.48 | 0.980 | 0.002-1 | 4.43 |
| imidazolepropionic acid | $C_6H_8N_2O_2$ | [M+H]+ | 141.06585 | 1.38 | 0.988 | 0.002-1 | 9.89 |
| isobutyrylcarnitine | $C_{11}H_{21}NO_4$ | [M+H]+ | 232.15386 | 6.48 | 1.000 | 0.002-1 | 6.71 |
| kynurenic acid | $C_{10}H_7NO_3$ | [M+H]+ | 190.04987 | 7.83 | 0.993 | 0.002-1 | 7.29 |
| kynurenine | $C_{10}H_{12}N_2O_3$ | [M+H]+ | 209.09207 | 4.55 | 0.998 | 0.002-1 | 9.91 |
| lactic acid | $C_3H_6O_3$ | [M-H]- | 89.0244173 | 1.43 | 0.955 | 0.002-1 | 5.60 |
| arginine | $C_6H_{14}N_4O_2$ | [M+H]+ | 175.11895 | 0.78 | 0.792 | 0.02-1 | 9.01 |
| carnitine | $C_7H_{15}NO_3$ | [M+H]+ | 162.1125 | 0.87 | 0.982 | 0.002-0.1 | 7.16 |
| citrulline | $C_6H_{13}N_3O_3$ | [M+H]+ | 176.10297 | 0.97 | 0.906 | 0.01-1 | 24.04 |

| | | | | | | | |
|---|---|---|---|---|---|---|---|
| dehydroascorbic acid | $C_6H_6O_6$ | [M-H]- | 173.00916 | 1.16 | 0.864 | 0.02-1 | 7.00 |
| glutamine | $C_5H_{10}N_2O_3$ | [M+H]+ | 147.07642 | 0.86 | 0.630 | 0.002-0.2 | 5.93 |
| linoleamide | $C_{18}H_{33}NO$ | [M+H]+ | 280.26349 | 14.71 | 0.823 | 0.002-0.1 | 8.45 |
| isoleucine | $C_6H_{13}NO_2$ | [M+H]+ | 132.10191 | 2.2 | 0.999 | 0.002-1 | 9.90 |
| leucine | $C_6H_{13}NO_2$ | [M+H]+ | 132.10191 | 2.4 | 0.999 | 0.002-1 | 8.75 |
| lysine | $C_6H_{14}N_2O_2$ | [M+H]+ | 147.1128 | 0.75 | 0.883 | 0.002-0.2 | 10.19 |
| methionine | $C_5H_{11}NO_2S$ | [M+H]+ | 150.05833 | 1.42 | 0.986 | 0.002-1 | 10.65 |
| phenylalanine | $C_9H_{11}NO_2$ | [M+H]+ | 166.08626 | 5.09 | 1.000 | 0.002-1 | 5.88 |
| pyroglutamic acid | $C_5H_7NO_3$ | [M+H]+ | 130.04987 | 1.6 | 0.993 | 0.002-1 | 3.39 |
| threonine | $C_4H_9NO_3$ | [M+H]+ | 120.06552 | 0.87 | 0.981 | 0.002-0.2 | 11.06 |
| tyrosine | $C_9H_{11}NO_3$ | [M+H]+ | 182.08117 | 1.83 | 0.999 | 0.002-1 | 4.10 |
| valine 5-aminovaleric acid | $C_5H_{11}NO_2$ | [M+H]+ | 118.08626 | 1.13 | 0.986 | 0.002-1 | 8.06 |
| lyso-phosphatidylcholine (18:0) | $C_{26}H_{52}NO_7P$ | [M+H]+ | 522.35542 | 13.67 | 0.985 | 0.002-1 | 18.45 |
| malic acid | $C_4H_6O_5$ | [M-H]- | 133.01424 | 1.19 | 0.981 | 0.002-0.2 | 8.42 |
| malonylcarnitine | $C_{10}H_{17}NO_6$ | [M+H]+ | 248.11286 | 1.13 | 0.893 | 0.02-1 | 5.93 |
| mannose | $C_6H_{12}O_6$ | [M-H]- | 179.05611 | 1 | 0.916 | 0.002-1 | 2.86 |
| N,N-dimethylarginine | $C_8H_{18}N_4O_2$ | [M+H]+ | 203.15025 | 0.93 | 0.997 | 0.002-0.1 | 8.64 |
| N6-methyladenosine | $C_{11}H_{15}N_5O_4$ | [M+H]+ | 282.11968 | 4.2 | 0.999 | 0.002-1 | 6.70 |
| N-acetylarginine | $C_8H_{16}N_4O_3$ | [M+H]+ | 217.12952 | 1.16 | 0.981 | 0.002-1 | 14.71 |
| N-acetylasparagine | $C_6H_{10}N_2O_4$ | [M+H]+ | 175.07133 | 1.16 | 0.980 | 0.002-0.1 | 15.31 |
| N-acetyl-beta-alanine | $C_5H_9NO_3$ | [M+H]+ | 132.06552 | 1.68 | 0.946 | 0.002-1 | 13.34 |
| N-acetylglutamic acid | $C_7H_{11}NO_5$ | [M+H]+ | 190.071 | 1.76 | 0.995 | 0.002-1 | 9.44 |
| N-acetyl-L-methionine | $C_7H_{13}NO_3S$ | [M+H]+ | 192.06889 | 7.6 | 0.995 | 0.002-1 | 15.42 |
| N-acetyl-L-phenylalanine | $C_{11}H_{13}NO_3$ | [M+H]+ | 208.09682 | 10.17 | 0.994 | 0.002-1 | 7.33 |
| N-acetyltryptophan | $C_{13}H_{14}N_2O_3$ | [M+H]+ | 247.1077 | 10.42 | 0.994 | 0.002-1 | 11.85 |
| N-acetyltyrosine | $C_{11}H_{13}NO_4$ | [M+H]+ | 224.09173 | 7.67 | 0.993 | 0.002-1 | 13.47 |
| N-acetylvaline/N-isovalerylglycine | $C_7H_{13}NO_3$ | [M+H]+ | 160.09682 | 7.6 | 0.997 | 0.002-1 | 12.85 |
| nicotinamide | $C_6H_6N_2O$ | [M+H]+ | 123.05530 | 1.59 | 0.977 | 0.002-1 | 6.01 |
| O-Acetyl-L carnitine | $C_9H_{17}NO_4$ | [M+H]+ | 204.12303 | 1.5 | 0.998 | 0.002-1 | 12.28 |
| oleamide | $C_{18}H_{35}NO$ | [M+H]+ | 282.27914 | 15.34 | 0.673 | 0.002-0.1 | 10.15 |

| | | | | | | | |
|---|---|---|---|---|---|---|---|
| O-succinylcarnitine | $C_{11}H_{19}NO_6$ | [M+H]+ | 262.12851 | 1.75 | 0.999 | 0.002-1 | 3.11 |
| oxoglutaric acid | $C_5H_6O_5$ | [M+H]+ | 147.02880 | 1.4 | 0.957 | 0.002-1 | 8.89 |
| pantothenol | $C_9H_{19}NO_4$ | [M+H]+ | 206.13868 | 6.63 | 0.927 | 0.002-0.5 | 5.97 |
| phenylacetaldehyde | $C_8H_8O$ | [M+H]+ | 121.06479 | 11 | 0.689 | 0.02-1 | 7.25 |
| phenylAc-Gln-OH | $C_{13}H_{16}N_2O_4$ | [M+H]+ | 265.11828 | 8.54 | 0.996 | 0.002-1 | 8.03 |
| propionyl carnitine C3 | $C_{10}H_{19}NO_4$ | [M+H]+ | 218.13868 | 3.01 | 0.998 | 0.002-1 | 8.22 |
| pyruvic acid | $C_3H_4O_3$ | [M-H]- | 87.00877 | 1.16 | 0.905 | 0.002-1 | 9.98 |
| saccharic acid | $C_6H_{10}O_8$ | [M-H]- | 209.03029 | 1.04 | 0.987 | 0.002-0.1 | 10.16 |
| sebacic acid | $C_{10}H_{18}O_4$ | [M-H]- | 201.11323 | 9.03 | 0.996 | 0.002-1 | 6.69 |
| sodium taurocholate (TCA) | $C_{26}H_{44}NNaO_7S$ | [M-H]- | 514.28440 | 11.26 | 0.994 | 0.002-1 | 8.58 |
| spermidine | $C_7H_{19}N_3$ | [M+H]+ | 146.16517 | 0.75 | 0.950 | 0.002-1 | 18.84 |
| succinic acid | $C_4H_6O_4$ | [M-H]- | 117.01933 | 1.96 | 0.999 | 0.002-1 | 5.90 |
| threonic acid | $C_4H_8O_5$ | [M+H]+ | 137.04445 | 0.95 | 1.000 | 0.002-0.2 | 5.28 |
| tryptophan | $C_{11}H_{12}N_2O_2$ | [M+H]+ | 205.09715 | 7.5 | 1.000 | 0.002-1 | 8.33 |
| valerylcarnitine | $C_{12}H_{23}NO_4$ | [M+H]+ | 246.16940 | 8.2 | 0.991 | 0.002-1 | 13.74 |

IP: Instrumental precision; The $R^2$ is shown as obtained for the mentioned dilution range.

**Table S4: Results for the univariate analysis performed for the targeted metabolites included in the adult dog cohort for comparison of IE with non-brain related diseases. Mean, median and standard deviation (SD) for the iQC normalized peak areas is mentioned (ratio: peak area metabolite/peak area iQC).**

| **Metabolite** | **Hypothesis test** | **Group Nrs** | **Amount per group** | **Mean per group** | **Median per group** | **SD per group** | ***P*-value** | **Fold change** | **Log2FC** |
|---|---|---|---|---|---|---|---|---|---|
| 2-hydroxy 3-methylvaleric acid | Wilcoxon rank sum exact test | -1; 1 | 7; 8 | 0.898; 1.351 | 1.078; 1.435 | 0.385; 0.502 | 0.151981352 | 0.664692823 | -0.589240319 |
| 2-hydroxyhexanoic_acid | Wilcoxon rank sum exact test | -1; 1 | 7; 8 | 0.850; 1.484 | 1.011; 1.597 | 0.358; 0.622 | 0.093861694 | 0.572776283 | -0.803956338 |
| 2-hydroxyisocaproic_acid | Welch Two Sample t-test | -1; 1 | 7; 8 | 0.880; 1.173 | 1.063; 1.328 | 0.367; 0.327 | 0.130002619 | 0.750213131 | -0.41462758 |
| 2-methylbutyryl-L-carnitine | Wilcoxon rank sum exact test | -1; 1 | 7; 8 | 2.025; 0.557 | 1.356; 0.685 | 2.161; 0.272 | 0.093861694 | **3.635547529** | **1.862172657** |
| **2-phenylethylamine** | Welch Two Sample t-test | -1; 1 | 7; 8 | 0.865; 0.972 | 0.864; 0.960 | 0.029; 0.102 | **0.021074691** | 0.889917697 | -0.168256179 |
| 2-piperidinone | Wilcoxon rank sum exact test | -1; 1 | 7; 8 | 0.781; 1.075 | 0.858; 0.883 | 0.381; 0.889 | 0.866511267 | 0.72651163 | -0.460942201 |
| 3-indoleacetic_acid | Welch Two Sample t-test | -1; 1 | 7; 8 | 1.089; 1.175 | 0.873; 1.129 | 0.684; 0.526 | 0.792555639 | 0.926808511 | -0.109656802 |
| 3-methylbutyryl_Isovalarayl-L-carnitine | Wilcoxon rank sum exact test | -1; 1 | 7; 8 | 2.119; 0.529 | 1.501; 0.581 | 2.240; 0.262 | 0.072105672 | **4.005671021** | **2.00204394** |
| 4-guanidinobutyric_acid | Welch Two Sample t-test | -1; 1 | 7; 8 | 1.039; 0.854 | 0.974; 0.771 | 0.586; 0.317 | 0.475523758 | 1.216627632 | 0.282887676 |
| 4-hydroxyphenylpyruvic_acid | Wilcoxon rank sum exact test | -1; 1 | 7; 8 | 0.895; 1.298 | 0.522; 1.074 | 1.064; 0.740 | 0.120590521 | 0.689522344 | -0.536330791 |
| 4-methyl_2-oxovaleric_acid | Welch Two Sample t-test | -1; 1 | 7; 8 | 0.789; 1.288 | 0.688; 1.268 | 0.380; 0.511 | 0.050306309 | 0.612577643 | -0.707035381 |
| 5-hydroxyindole_acetic_acid | Wilcoxon rank sum exact test | -1; 1 | 7; 8 | 0.840; 1.249 | 0.668; 0.963 | 0.558; 0.695 | 0.151981352 | 0.672538033 | -0.572312238 |
| 7-methylguanine | Welch Two Sample t-test | -1; 1 | 7; 8 | 0.915; 1.285 | 0.818; 1.301 | 0.384; 0.270 | 0.05698107 | 0.712062259 | -0.489924706 |
| ***acetophenone*** | Wilcoxon rank sum exact test | -1; 1 | 7; 8 | 2.765; 1.031 | 1.082; 1.027 | 4.455; 0.033 | **0.009324009** | **2.681862253** | **1.423235139** |

| | | | | | | | | | |
|---|---|---|---|---|---|---|---|---|---|
| acetylcholine | Wilcoxon rank sum exact test | -1; 1 | 7; 8 | 1.188; 0.774 | 1.050; 0.752 | 0.572; 0.189 | 0.054079254 | 1.534883714 | 0.618129358 |
| adenine | Wilcoxon rank sum exact test | -1; 1 | 7; 8 | 2.098; 0.169 | 0.194; 0.162 | 3.975; 0.073 | 0.280963481 | **12.41420051** | **3.633919448** |
| adenosine-5_monophosphate | Wilcoxon rank sum exact test | -1; 1 | 7; 8 | 2.068; 1.238 | 2.007; 0.267 | 2.357; 2.860 | 0.463403263 | 1.670436182 | 0.740224866 |
| adipoylcarnitine_C16 | Welch Two Sample t-test | -1; 1 | 7; 8 | 0.658; 0.345 | 0.589; 0.344 | 0.574; 0.178 | 0.207505787 | 1.907246351 | 0.931491202 |
| alfa-hydroxyisobutyric_acid | Welch Two Sample t-test | -1; 1 | 7; 8 | 1.095; 1.070 | 1.203; 1.093 | 0.490; 0.325 | 0.910812613 | 1.023364486 | 0.033320073 |
| allantoin | Welch Two Sample t-test | -1; 1 | 7; 8 | 1.496; 0.534 | 1.119; 0.510 | 1.126; 0.220 | 0.065110555 | **2.801498094** | **1.486198511** |
| anandamide_C18_1 | Wilcoxon rank sum exact test | -1; 1 | 7; 8 | 0.303; 2.058 | 0.303; 0.516 | 0.158; 3.140 | 0.072105672 | 0.147230325 | -2.763853243 |
| azelaic_acid | Welch Two Sample t-test | -1; 1 | 7; 8 | 1.317; 1.449 | 1.332; 1.353 | 0.256; 0.270 | 0.349485268 | 0.908902692 | -0.137802248 |
| B-hydroxyisovaleric_acid | Welch Two Sample t-test | -1; 1 | 7; 8 | 0.911; 1.135 | 0.968; 1.133 | 0.329; 0.275 | 0.181699267 | 0.802643174 | -0.317169335 |
| choline | Wilcoxon rank sum exact test | -1; 1 | 7; 8 | 1.008; 0.502 | 0.507; 0.503 | 0.929; 0.088 | 0.866511267 | **2.007968107** | **1.005736355** |
| **cortisol** | Welch Two Sample t-test | -1; 1 | 7; 8 | 0.228; 0.989 | 0.111; 0.864 | 0.219; 0.642 | **0.012121801** | 0.230535903 | -2.116936648 |
| creatine | Welch Two Sample t-test | -1; 1 | 7; 8 | 1.310; 1.123 | 1.001; 1.070 | 0.890; 0.331 | 0.615508419 | 1.166518253 | 0.222208882 |
| **creatinine** | Welch Two Sample t-test | -1; 1 | 7; 8 | 0.927; 1.426 | 0.890; 1.452 | 0.167; 0.303 | **0.001992792** | 0.650070129 | -0.621332732 |
| cytidine | Wilcoxon rank sum exact test | -1; 1 | 7; 8 | 2.608; 0.421 | 0.697; 0.381 | 3.394; 0.134 | 0.335664336 | **6.194774223** | **2.631051702** |
| cytosine | Wilcoxon rank sum exact test | -1; 1 | 7; 8 | 2.020; 1.070 | 1.229; 0.876 | 1.638; 0.420 | 0.189277389 | 1.887850459 | 0.91674449 |
| **glucose** | Welch Two Sample t-test | -1; 1 | 7; 8 | 0.809; 1.243 | 0.958; 1.195 | 0.330; 0.168 | **0.012375596** | 0.650844733 | -0.619614682 |
| dipropyl_disulfide | Welch Two Sample t-test | -1; 1 | 7; 8 | 1.135; 1.130 | 1.145; 1.083 | 0.457; 0.406 | 0.980637629 | 1.004424779 | 0.006369525 |

| | | | | | | | | | |
|---|---|---|---|---|---|---|---|---|---|
| dodecanedioic_acid | Welch Two Sample t-test | -1; 1 | 7; 8 | 0.974; 1.080 | 0.917; 1.094 | 0.150; 0.188 | 0.248746913 | 0.901851853 | -0.149037634 |
| pantothenic_acid | Welch Two Sample t-test | -1; 1 | 7; 8 | 0.943; 1.327 | 0.733; 1.237 | 0.850; 0.781 | 0.381307011 | 0.710625473 | -0.49283869 |
| GABA_2-aminoisobutyric_acid | Welch Two Sample t-test | -1; 1 | 7; 8 | 0.928; 1.035 | 0.527; 1.036 | 0.686; 0.511 | 0.740450925 | 0.896618358 | -0.157434056 |
| gamma-glutamylphenylalanine | Wilcoxon rank sum exact test | -1; 1 | 7; 8 | 1.209; 1.130 | 1.153; 1.081 | 0.713; 0.505 | 0.866511267 | 1.069911504 | 0.097491471 |
| glyceric_acid | Welch Two Sample t-test | -1; 1 | 7; 8 | 0.972; 1.104 | 0.996; 1.148 | 0.260; 0.403 | 0.457234393 | 0.880434784 | -0.183711951 |
| hexadecamide | Welch Two Sample t-test | -1; 1 | 7; 8 | 1.093; 1.137 | 0.706; 1.026 | 0.895; 0.736 | 0.918534234 | 0.961301671 | -0.056938853 |
| **hippuric_acid** | Wilcoxon rank sum exact test | -1; 1 | 7; 8 | 0.599; 1.933 | 0.173; 1.477 | 1.009; 1.556 | **0.013986014** | 0.309881018 | -1.690213713 |
| homocysteine | Welch Two Sample t-test | -1; 1 | 7; 8 | 1.197; 1.318 | 1.479; 1.264 | 0.492; 0.542 | 0.657965237 | 0.908194234 | -0.138927217 |
| hypoxanthine | Wilcoxon rank sum exact test | -1; 1 | 7; 8 | 2.492; 0.110 | 0.977; 0.090 | 3.913; 0.064 | 0.335664336 | **22.65454349** | **4.501728514** |
| imidazolepropionic_acid | Wilcoxon rank sum exact test | -1; 1 | 7; 8 | 0.794; 1.098 | 0.629; 0.979 | 0.781; 0.627 | 0.231857032 | 0.723132972 | -0.467667137 |
| isobutyrylcarnitine | Wilcoxon rank sum exact test | -1; 1 | 7; 8 | 2.267; 0.441 | 0.547; 0.446 | 3.204; 0.117 | 0.280963481 | **5.140589475** | **2.361933804** |
| kynurenic_acid | Wilcoxon rank sum exact test | -1; 1 | 7; 8 | 1.162; 0.703 | 0.427; 0.594 | 1.624; 0.457 | 0.866511267 | 1.652916065 | 0.725013466 |
| kynurenine | Welch Two Sample t-test | -1; 1 | 7; 8 | 1.220; 1.226 | 0.995; 1.338 | 0.897; 0.436 | 0.987000087 | 0.995106036 | -0.007077831 |
| lactic_acid | Welch Two Sample t-test | -1; 1 | 7; 8 | 1.310; 0.726 | 1.195; 0.801 | 0.721; 0.156 | 0.076929028 | 1.804407702 | 0.85152535 |
| arginine | Welch Two Sample t-test | -1; 1 | 7; 8 | 1.002; 1.171 | 0.924; 1.109 | 0.345; 0.545 | 0.482617374 | 0.855678908 | -0.224858565 |
| carnitine | Wilcoxon rank sum exact test | -1; 1 | 7; 8 | 1.117; 1.328 | 0.774; 1.147 | 0.810; 0.485 | 0.231857032 | 0.841114459 | -0.249625959 |
| citrulline | Welch Two Sample t-test | -1; 1 | 7; 8 | 0.828; 0.989 | 0.934; 0.887 | 0.772; 0.441 | 0.638512182 | 0.837209304 | -0.25633975 |

| | | | | | | | | | |
|---|---|---|---|---|---|---|---|---|---|
| glutamine | Welch Two Sample t-test | -1; 1 | 7; 8 | 1.070; 1.109 | 1.094; 1.068 | 0.223; 0.220 | 0.735326258 | 0.964833183 | -0.051648568 |
| linoleamide | Welch Two Sample t-test | -1; 1 | 7; 8 | 0.926; 1.122 | 0.611; 1.086 | 0.745; 0.626 | 0.595654255 | 0.825311945 | -0.276988575 |
| isoleucine | Welch Two Sample t-test | -1; 1 | 7; 8 | 1.118; 1.158 | 1.029; 1.367 | 0.396; 0.449 | 0.857146927 | 0.965457686 | -0.050715065 |
| leucine | Welch Two Sample t-test | -1; 1 | 7; 8 | 1.049; 1.215 | 1.167; 1.313 | 0.292; 0.417 | 0.385563816 | 0.863374487 | -0.211941634 |
| lysine | Welch Two Sample t-test | -1; 1 | 7; 8 | 1.027; 1.164 | 1.034; 1.114 | 0.251; 0.347 | 0.392159148 | 0.882302407 | -0.180654875 |
| methionine | Welch Two Sample t-test | -1; 1 | 7; 8 | 1.106; 1.187 | 1.110; 1.179 | 0.428; 0.420 | 0.721097242 | 0.931760742 | -0.101968549 |
| phenylalanine | Welch Two Sample t-test | -1; 1 | 7; 8 | 1.141; 1.175 | 1.102; 1.192 | 0.353; 0.303 | 0.847507548 | 0.97106383 | -0.042361965 |
| pyroglutamic_acid | Welch Two Sample t-test | -1; 1 | 7; 8 | 0.754; 0.917 | 0.742; 0.856 | 0.185; 0.349 | 0.274201362 | 0.822246458 | -0.282357207 |
| threonine | Welch Two Sample t-test | -1; 1 | 7; 8 | 0.932; 1.408 | 0.811; 1.340 | 0.393; 0.555 | 0.075875036 | 0.661931821 | -0.595245469 |
| tyrosine | Welch Two Sample t-test | -1; 1 | 7; 8 | 1.064; 1.235 | 1.059; 1.136 | 0.332; 0.426 | 0.398336505 | 0.861538463 | -0.215012889 |
| malic_acid | Welch Two Sample t-test | -1; 1 | 7; 8 | 1.436; 0.689 | 0.991; 0.682 | 1.401; 0.418 | 0.217207082 | **2.084179955** | **1.05947985** |
| malonylcarnitine | Welch Two Sample t-test | -1; 1 | 7; 8 | 1.469; 1.335 | 1.632; 1.303 | 0.777; 0.772 | 0.74413867 | 1.100374531 | 0.137994653 |
| **mannose** | Welch Two Sample t-test | -1; 1 | 7; 8 | 0.810; 1.237 | 0.967; 1.193 | 0.328; 0.167 | **0.013054937** | 0.654810027 | -0.610851681 |
| methoxytyramine | Wilcoxon rank sum exact test | -1; 1 | 7; 8 | 1.890; 0.724 | 1.553; 0.704 | 1.593; 0.302 | 0.093861694 | **2.610497215** | **1.38432462** |
| N_N-dimethylarginine | Wilcoxon rank sum exact test | -1; 1 | 7; 8 | 1.138; 1.838 | 1.174; 1.380 | 0.299; 1.089 | 0.280963481 | 0.619151253 | -0.691636204 |
| N6-acetyl-L-lysine | Welch Two Sample t-test | -1; 1 | 7; 8 | 0.943; 1.723 | 0.979; 1.639 | 0.283; 1.009 | 0.068768384 | 0.547301221 | -0.869593019 |
| N6-methyladenosine | Welch Two Sample t-test | -1; 1 | 7; 8 | 0.628; 0.795 | 0.583; 0.693 | 0.277; 0.290 | 0.275240414 | 0.78993711 | -0.340190296 |

| | | | | | | | | | |
|---|---|---|---|---|---|---|---|---|---|
| N-acetyl_arginine | Welch Two Sample t-test | -1; 1 | 7; 8 | 0.975; 1.301 | 0.869; 1.354 | 0.297; 0.370 | 0.081022013 | 0.749423522 | -0.416146834 |
| N-acetyl_asparagine | Welch Two Sample t-test | -1; 1 | 7; 8 | 1.128; 0.788 | 1.114; 0.660 | 0.475; 0.455 | 0.182876989 | 1.431472076 | 0.517499527 |
| N-acetyl-beta-alanine | Wilcoxon rank sum exact test | -1; 1 | 7; 8 | 0.989; 1.000 | 0.898; 0.819 | 0.485; 0.443 | 0.955089355 | 0.989 | -0.015957574 |
| N-acetylglutamic_acid | Welch Two Sample t-test | -1; 1 | 7; 8 | 1.174; 1.149 | 0.963; 1.130 | 0.886; 0.418 | 0.94570414 | 1.02175805 | 0.03105361 |
| N-acetyl-L-methionine | Wilcoxon rank sum exact test | -1; 1 | 7; 8 | 1.420; 0.996 | 1.059; 0.871 | 1.392; 0.444 | 0.612587413 | 1.425702807 | 0.511673278 |
| N-acetyl-L-phenylalanine | Welch Two Sample t-test | -1; 1 | 7; 8 | 1.047; 1.184 | 1.199; 1.121 | 0.454; 0.381 | 0.541231224 | 0.884290542 | -0.177407637 |
| N-acetyltryptophan | Welch Two Sample t-test | -1; 1 | 7; 8 | 0.938; 1.071 | 0.943; 0.985 | 0.328; 0.389 | 0.486328863 | 0.875816995 | -0.19129865 |
| N-acetyltyrosine | Welch Two Sample t-test | -1; 1 | 7; 8 | 0.828; 1.190 | 0.968; 1.134 | 0.360; 0.431 | 0.099567327 | 0.695798322 | -0.523258895 |
| N-acetylvaline | Welch Two Sample t-test | -1; 1 | 7; 8 | 1.034; 1.132 | 1.110; 1.027 | 0.279; 0.321 | 0.537189093 | 0.913427563 | -0.130637771 |
| nicotinamide | Wilcoxon rank sum exact test | -1; 1 | 7; 8 | 1.110; 1.291 | 0.975; 1.050 | 0.779; 0.709 | 0.535819736 | 0.859798607 | -0.217929322 |
| O-acetyl L-carnitine | Welch Two Sample t-test | -1; 1 | 7; 8 | 1.318; 0.611 | 0.737; 0.561 | 1.083; 0.269 | 0.138615309 | **2.157119457** | **1.109106073** |
| oleamide | Welch Two Sample t-test | -1; 1 | 7; 8 | 0.808; 0.986 | 0.691; 1.015 | 0.438; 0.289 | 0.380449461 | 0.819472618 | -0.28723235 |
| O-succinylcarnitine | Welch Two Sample t-test | -1; 1 | 7; 8 | 1.122; 1.031 | 1.219; 1.040 | 0.491; 0.323 | 0.684475796 | 1.088263821 | 0.122028342 |
| oxoglutaric_acid | Welch Two Sample t-test | -1; 1 | 7; 8 | 1.017; 1.115 | 0.965; 1.179 | 0.395; 0.360 | 0.626353172 | 0.912107624 | -0.13272403 |
| **pantothenol** | Welch Two Sample t-test | -1; 1 | 7; 8 | 0.595; 1.820 | 0.646; 1.666 | 0.141; 1.164 | **0.020570625** | 0.326923081 | -1.612976861 |
| phenylacetalaldehyde | Welch Two Sample t-test | -1; 1 | 7; 8 | 1.041; 1.038 | 1.048; 1.056 | 0.056; 0.051 | 0.909017879 | 1.002890173 | 0.004163625 |
| phenylAc-GLN-OH | Wilcoxon rank sum exact test | -1; 1 | 7; 8 | 0.903; 0.991 | 0.639; 0.776 | 0.690; 0.752 | 0.612587413 | 0.911200808 | -0.134159068 |

| | | | | | | | | | |
|---|---|---|---|---|---|---|---|---|---|
| propionyl_carnitine_C3 | Welch Two Sample t-test | -1; 1 | 7; 8 | 1.755; 0.656 | 1.510; 0.503 | 1.696; 0.364 | 0.140253299 | **2.675304853** | **1.419703297** |
| pyruvic_acid | Welch Two Sample t-test | -1; 1 | 7; 8 | 1.086; 1.028 | 0.835; 0.900 | 0.832; 0.761 | 0.890835996 | 1.056420233 | 0.079183838 |
| saccharic_acid | Wilcoxon rank sum exact test | -1; 1 | 7; 8 | 0.845; 0.971 | 0.728; 0.986 | 0.240; 0.275 | 0.463403263 | 0.870236871 | -0.200519952 |
| sebacic_acid | Wilcoxon rank sum exact test | -1; 1 | 7; 8 | 0.850; 1.054 | 0.835; 0.941 | 0.094; 0.294 | 0.054079254 | 0.806451615 | -0.310340117 |
| **spermidine** | Wilcoxon rank sum exact test | -1; 1 | 7; 8 | 2.371; 0.377 | 1.013; 0.173 | 3.099; 0.622 | **0.020512821** | **6.289124528** | **2.652859202** |
| succinic_acid | Wilcoxon rank sum exact test | -1; 1 | 7; 8 | 2.053; 0.518 | 1.088; 0.509 | 2.530; 0.099 | 0.463403263 | **3.963320406** | **1.986709604** |
| TCA | Wilcoxon rank sum exact test | -1; 1 | 7; 8 | 1.625; 0.329 | 0.944; 0.310 | 2.422; 0.160 | 0.335664336 | **4.939209607** | **2.304280194** |
| threonic_acid | Welch Two Sample t-test | -1; 1 | 7; 8 | 1.275; 1.077 | 1.152; 1.033 | 0.487; 0.340 | 0.388410583 | 1.183844009 | 0.243478995 |
| tryptophan | Welch Two Sample t-test | -1; 1 | 7; 8 | 1.115; 1.050 | 1.029; 1.117 | 0.388; 0.260 | 0.715023566 | 1.061904761 | 0.086654381 |
| valerylcarnitine_C5 | Wilcoxon rank sum exact test | -1; 1 | 7; 8 | 2.241; 0.548 | 1.502; 0.582 | 2.416; 0.291 | 0.072105672 | **4.089416002** | **2.031894831** |
| valine_aminovaleric_acid | Welch Two Sample t-test | -1; 1 | 7; 8 | 1.135; 1.073 | 1.117; 1.116 | 0.427; 0.430 | 0.78500911 | 1.057781919 | 0.081042221 |

**Table S5: Table showing the untargeted components with discriminative potential.**

| ID | m/z value | RT (min) | VIP score | P corr S plot | JK CI (D*E) |
|---|---|---|---|---|---|
| **1** | 293.19874 | 11.60 | 2.81598 | 0.69644 | 6.64734E-06 |
| **4** | 125.08411 | 7.40 | 2.59233 | -0.61207 | 1.47726E-05 |
| **8** | 264.18356 | 10.82 | 2.64063 | 0.66017 | 1.72253E-05 |
| **9** | 339.20479 | 11.60 | 2.61077 | 0.65039 | 1.17662E-05 |
| **10** | 230.24366 | 11.63 | 1.50779 | -0.63352 | 3.87739E-05 |
| **41** | 282.16765 | 7.59 | 2.00021 | 0.71829 | 5.00631E-05 |
| **45** | 356.20413 | 8.00 | 2.05838 | 0.72050 | 5.75819E-05 |
| **60** | 378.18594 | 8.00 | 2.16168 | 0.71037 | 5.88242E-05 |
| **72** | 398.21452 | 9.49 | 2.06962 | 0.74461 | 4.70705E-05 |
| **76** | 420.19638 | 9.49 | 2.15114 | 0.72985 | 4.88068E-05 |
| **78** | 331.17314 | 11.61 | 2.30644 | 0.67121 | 1.53912E-05 |
| **92** | 304.14932 | 7.62 | 2.02798 | 0.70891 | 5.49126E-05 |
| **93** | 151.06322 | 6.75 | 2.12181 | -0.60714 | 1.19258E-05 |
| **95** | 208.12092 | 5.49 | 1.90844 | 0.62026 | 5.98118E-06 |
| **132** | 362.09669 | 1.99 | 1.71477 | 0.60774 | 5.1111E-06 |
| **135** | 515.32977 | 11.11 | 1.70977 | -0.60079 | 5.29422E-05 |
| **149** | 416.18092 | 10.73 | 1.70366 | 0.69675 | 2.06747E-05 |
| **156** | 466.17294 | 10.81 | 1.72140 | 0.71673 | 3.29623E-05 |
| **157** | 294.11947 | 11.36 | 2.04069 | 0.64972 | 6.03135E-07 |
| **161** | 330.24047 | 11.72 | 1.50455 | -0.82384 | 3.87873E-05 |
| **167** | 396.15212 | 10.11 | 1.80695 | 0.72670 | 1.9915E-05 |
| **169** | 438.16255 | 10.74 | 1.78216 | 0.69003 | 2.48526E-05 |
| **172** | 175.0996 | 10.82 | 2.04086 | 0.67003 | 1.74936E-05 |
| **178** | 420.17684 | 10.02 | 1.99908 | 0.77007 | 4.33639E-05 |
| **188** | 402.21004 | 8.00 | 2.09323 | 0.74705 | 5.63253E-05 |
| **194** | 171.10234 | 7.41 | 2.04116 | -0.60119 | 1.02771E-05 |
| **206** | 488.15473 | 10.81 | 1.72747 | 0.70240 | 2.9108E-05 |
| **207** | 328.17361 | 7.62 | 1.99732 | 0.73146 | 4.06492E-05 |
| **219** | 300.13378 | 9.73 | 1.72319 | 0.73074 | 2.73663E-05 |
| **236** | 380.20398 | 9.49 | 2.01116 | 0.75166 | 3.28233E-05 |
| **243** | 298.25039 | 14.33 | 1.54343 | -0.87580 | 2.99331E-05 |
| **244** | 494.21353 | 10.05 | 2.05193 | 0.77464 | 5.24731E-05 |
| **245** | 554.20974 | 10.97 | 1.73963 | 0.65169 | 3.50845E-06 |
| **247** | 444.22106 | 9.49 | 1.99323 | 0.73962 | 3.57266E-05 |
| **261** | 324.17778 | 9.38 | 1.73094 | 0.69593 | 9.51154E-06 |
| **266** | 283.09134 | 3.14 | 1.56833 | 0.61263 | 6.89819E-06 |
| **267** | 346.1597 | 9.38 | 1.82148 | 0.69317 | 1.7202E-05 |
| **270** | 244.07685 | 11.69 | 1.74155 | -0.79882 | 4.02315E-05 |
| **288** | 228.13788 | 11.34 | 1.80940 | 0.63066 | 8.76562E-07 |
| **311** | 123.03862 | 10.67 | 1.68049 | -0.69245 | 1.31629E-05 |
| **313** | 420.19661 | 9.34 | 2.15034 | 0.78609 | 4.97335E-05 |
| **315** | 379.18943 | 8.00 | 1.95351 | 0.74720 | 5.24324E-05 |
| **317** | 536.22413 | 10.65 | 1.83788 | 0.73668 | 3.22166E-05 |
| **330** | 421.19968 | 9.49 | 1.90558 | 0.75066 | 3.63956E-05 |

| ID | m/z value | RT (min) | VIP score | P corr S plot | JK CI (D*E) |
|---|---|---|---|---|---|
| **331** | 322.11545 | 9.73 | 1.78678 | 0.73233 | 2.72306E-05 |
| **349** | 462.18747 | 10.73 | 1.81787 | 0.76340 | 2.23395E-05 |
| **354** | 691.43456 | 11.19 | 1.60167 | -0.60783 | 4.9975E-05 |
| **358** | 208.13101 | 6.65 | 1.56792 | 0.62114 | 2.8651E-06 |
| **379** | 420.17674 | 10.11 | 1.84061 | 0.76510 | 2.23757E-05 |
| **402** | 530.16515 | 11.15 | 1.60359 | 0.72574 | 1.25073E-05 |
| **411** | 398.17019 | 10.73 | 1.66111 | 0.71246 | 1.98414E-05 |
| **446** | 360.17577 | 9.00 | 1.75862 | 0.71418 | 7.94569E-06 |
| **450** | 306.16731 | 9.38 | 1.84879 | 0.73769 | 1.78382E-05 |
| **492** | 604.20179 | 11.01 | 1.76966 | 0.75640 | 3.69001E-05 |
| **527** | 512.17953 | 10.81 | 1.65030 | 0.76203 | 2.22826E-05 |
| **541** | 360.17577 | 8.70 | 1.71749 | 0.71566 | 1.48182E-05 |
| **591** | 472.20679 | 10.65 | 1.63109 | 0.70959 | 1.5415E-05 |
| **599** | 338.01103 | 10.68 | 1.59061 | -0.73732 | 3.34418E-05 |
| **602** | 305.15254 | 7.62 | 1.74098 | 0.72721 | 2.08718E-05 |
| **675** | 210.05231 | 9.67 | 1.59634 | -0.75998 | 2.84371E-05 |
| **688** | 370.18456 | 9.35 | 1.71661 | 0.73220 | 1.52714E-05 |
| **692** | 346.13994 | 9.72 | 1.65101 | 0.73184 | 1.26966E-05 |
| **775** | 596.21954 | 11.34 | 1.69724 | 0.76731 | 5.08734E-06 |

m/z: mass to charge ratio, RT: retention time; VIP: variable importance of projection, JK CI (D*E): Jack-Knifed confidence interval, lower * upper bound.

**Table S6: Targeted metabolites detected for the first time in CSF, as evaluated in the validation.**

| **Metabolite** | **Chemical Formula** | **Ion adduct** | **m/z-value** | **RT** | **Class** | **Detected before in** |
|---|---|---|---|---|---|---|
| saccharic acid | $C_6H_{10}O_8$ | [M-H]- | 209.03029 | 1.04 | organooxygen compounds | blood, faeces, urine |
| dipropyl disulfide | $C_6H_{14}S_2$ | [M+H]+ | 151.06097 | 1.5 | organic disulfides | faeces |
| N-acetyl-L-glutamic acid | $C_7H_{11}NO_5$ | [M+H]+ | 190.071 | 1.76 | carboxylic acids and derivatives | blood, faeces, urine, saliva |
| N-acetyl-L-methionine | $C_7H_{13}NO_3S$ | [M+H]+ | 192.06889 | 7.6 | carboxylic acids and derivatives | blood, faeces, urine, saliva |
| cytosine | $C_4H_5N_3O$ | [M+H]+ | 112.05054 | 1.5 | diazines | blood, faeces, urine |
| sodium taurocholate (TCA) | $C_{26}H_{44}NNaO_7S$ | [M-H]- | 514.2844 | 11.26 | steroids and derivatives | blood, faeces, urine, bile |
| 7-methylguanine | $C_6H_7N_5O$ | [M+H]+ | 166.07234 | 1.62 | imidazopyrimidines | blood, faeces, urine |
| gamma-glutamylphenylalanine | $C_{14}H_{18}N_2O_5$ | [M+H]+ | 295.12884 | 8.03 | carboxylic acids and derivatives | blood, faeces, urine |

**Table S7: Analytical standards and internal standards used in the study.**

| Analytical standard | Chemical Formula | Ion Adduct | m/z-value | Retention Time (min) | Optimization | Validation |
|---|---|---|---|---|---|---|
| alanine-d3 | $CD_3CH(NH_2)COOH$ | [M+H]+ | 93.07378 | 0.97 | ISTD | ISTD |
| dopamine.HCl-d4 | $C_8H_7D_4NO_2.HCl$ | [M+H]+ | 158.11113 | 1.47 | ISTD | ISTD |
| 1,3-propanediol | $C_3H_8O_2$ | [M+H]+ | 77.05971 | 1.33 | x | x |
| 1,3-dimethoxybenzene | $C_8H_{10}O_2$ | [M+H]+ | 139.07536 | 11.94 | | |
| 13-OxoODE | $C_{18}H_30O_3$ | [M+H]+ | 295.22599 | 13.58 | | |
| 1-acetyl-3-indolecarboxaldehyde | $C_{11}H_9NO_2$ | [M+H]+ | 188.07061 | 10.99 | | |
| 1-methylguanidine | $C_2H_8ClN_3$ | [M+H]+ | 74.01712 | 0.97 | | |
| 1-methylhistamine | $C_6H_{11}N_3$ | [M+H]+ | 126.10257 | 0.85 | | |
| 1-methyl-L-histidine | $C_7H_{11}N_3O_2$ | [M+H]+ | 170.0924 | 0.79 | x | |
| 1-octen-3-one | $C_8H_{14}O$ | [M+H]+ | 127.11174 | 12.46 | | |
| 2-(dimethyloamino)acetonitrile | $C_4H_8N_2$ | [M+H]+ | 85.07602 | 1 | | |
| 2,3-heptanedione | $C_7H_{12}O_2$ | [M+H]+ | 129.09101 | 8.4 | | |
| 2,3-pentanedione | $C_5H_8O_2$ | [M+H]+ | 101.05971 | 1.17 | | |
| 2.6-diaminopimelic acid | $C_7H_{14}N_2O_4$ | [M+H]+ | 191.10263 | 0.85 | x | |
| 2-acetyl-5-methylfuran | $C_7H_8O_2$ | [M+H]+ | 125.05971 | 10.47 | | |
| 2-deoxyuridine | $C_9H_{12}N_2O_5$ | [M+H]+ | 229.08190 | 2.74 | | |
| 2-dodecanone | $C_{12}H_{24}O$ | [M+H]+ | 185.18999 | 15 | | |
| 2-hexanone | $C_6H_{12}O$ | [M+H]+ | 101.09609 | 11.37 | | |
| 3-hydroxybutyric acid | $C_4H_8O_3$ | [M+H]+ | 105.05462 | 2.4 | x | |
| 2-hydroxyhexanoic acid | $C_6H_{12}O_3$ | [M-H]- | 131.0695 | 9.4 | x | x |
| 2-hydroxyisocaproic acid | $C_6H_{12}O_3$ | [M+H]+ | 131.06995 | 9.16 | x | x |
| 2-hydroxy 3-methylvalerate | $C_6H_{12}O_3$ | [M-H]- | 131.06950 | 9.03 | | x |
| 2-methylbutyryl-L-carnitine | $C_{12}H_{23}NO_4$ | [M+H]+ | 246.16998 | 8.55 | | x |
| 2-pentanone | $C_5H_{10}O$ | [M+H]+ | 87.08095 | 9.97 | | |
| 2-phenylethylamine | $C_8H_{11}N$ | [M+H]+ | 122.09643 | 6.59 | | x |
| 2-piperidinone | $C_5H_9NO$ | [M+H]+ | 100.07569 | 5 | x | x |
| 3,4-dihydroxybenzoic acid | $C_7H_6O_4$ | [M-H]- | 153.01933 | 4.7 | | |

| **Analytical standard** | **Chemical Formula** | **Ion Adduct** | **m/z-value** | **Retention Time (min)** | **Optimization** | **Validation** |
|---|---|---|---|---|---|---|
| 3,4-dihydroxyphenylalanine (L-DOPA) | $C_9H_{11}NO_4$ | [M+H]+ | 198.07608 | 1.55 | | |
| 3,4-Dihydroxyphenylpropionic acid | $C_9H_{10}O_4$ | [M+H]+ | 183.06519 | 8.89 | x | |
| 3,5-dihydroxybenzoic acid | $C_7H_6O_4$ | [M+H]+ | 155.03389 | 6.12 | | |
| 3-amino-2-piperidone | $C_5H_{10}N_2O$ | [M+H]+ | 115.08658 | 0.92 | | |
| 3-ethylphenol | $C_8H_{10}O$ | [M+H]+ | 123.08044 | 11.56 | | |
| 3-heptanone | $C_7H_{14}O$ | [M+H]+ | 115.11174 | 12.11 | | |
| 3-hydroxy DL-kynurenin | $C_{10}H_{12}N_2O_4$ | [M+H]+ | 225.08698 | 1.9 | | |
| 3-hydroxy-3-methylglutarate | $C_6H_{10}O_5$ | [M+H]+ | 163.06010 | 2.59 | | |
| 3-Hydroxy-4-methoxycinnamic acid | $C_{10}H_{10}O_4$ | [M+H]+ | 195.06519 | 10.44 | | |
| 3-hydroxypyridine | $C_8H_8O$ | [M+H]+ | 96.04439 | 1.08 | | |
| 3-indoleacetic acid | $C_{10}H_9NO_2$ | [M+H]+ | 176.07061 | 8.84 | x | x |
| 3-methoxytyramine hydroxychloride | $C_9H_{13}NO_2$ | [M+H]+ | 168.10191 | 4.4 | | x |
| 3-methyl-2-cyclohexen-1-one | $C_7H_{10}O$ | [M+H]+ | 111.08044 | 10.14 | | |
| 3-methyl-2-oxobutyric acid | $C_5H_8NaO_3$ | [M+H]+ | 117.05462 | 2.67 | | |
| 3-methyl-2-oxopentanoic acid (ketoisoleucine, pantolactone) | $C_6H_{10}O_3$ | [M+H]+ | 131.07027 | 3.56 | | |
| 3-methyladenine | $C_6H_7N_5$ | [M+H]+ | 150.07742 | 1.41 | | |
| 3-methylbutyryl/isovalaryl-L-carnitine | $C_{12}H_{23}NO_4$ | [M+H]+ | 246.16998 | 8.21 | x | x |
| 3-phenyl-1-propanol | $C_9H_{12}O$ | [M+H]+ | 137.09609 | 11.24 | x | |
| 3-phenylbutyric acid | $C_{10}H_{12}O_2$ | [M-H]- | 163.07572 | 11.47 | | |
| 3-phenylpropionic acid | $C_9H_{10}O_2$ | [M-H]- | 149.0608 | 9.2 | x | |
| 4-guanidinobutyric acid | $C_5H_{11}N_3O_2$ | [M+H]+ | 146.09220 | 1.44 | x | x |
| 4-hexen-3-one | $C_6H_{10}O$ | [M+H]+ | 99.08044 | 9.78 | | |
| 4-hydroxybenzaldehyde | $C_7H_6O_2$ | [M+H]+ | 123.04406 | 8.87 | | |
| 4-hydroxyphenylacetic acid | $C_8H_8O_3$ | [M+H]+ | 153.05462 | 9.4 | | |

| Analytical standard | Chemical Formula | Ion Adduct | m/z-value | Retention Time (min) | Optimization | Validation |
|---|---|---|---|---|---|---|
| 4-hydroxyphenylpyruvic acid | $C_9H_8O_4$ | [M-H]- | 179.03498 | 4.55 | | x |
| 4-methyl-2-oxovaleric acid | $C_6H_{10}O_3$ | [M-H]- | 129.05432 | 8.41 | x | x |
| 4-methylcatechol | $C_7H_8O_2$ | [M-H]- | 123.04515 | 8.1 | | |
| 4-methylvaleric acid | $C_6H_{12}O_2$ | [M+H]+ | 117.09071 | 9.26 | | |
| 5-hydroxyindole-3-acetic acid | $C_{10}H_9NO_3$ | [M+H]+ | 192.06552 | 8.04 | x | x |
| 5-hydroxytryptophol | $C_{10}H_{11}NO_2$ | [M+H]+ | 178.08626 | 7.86 | | |
| 6-acetyl-L-lysine | $C_8H_{16}N_2O_3$ | [M+H]+ | 189.12337 | 1.47 | x | x |
| 6-deoxyhexose | $C_6H_{12}O_5$ | [M+H]+ | 163.06060 | 1.12 | x | |
| 7-ketodeoxycholate | $C_{24}H_{38}O_5$ | [M-H]- | 405.26465 | 11.53 | | |
| 7-methylguanine | $C_6H_7N_5O$ | [M+H]+ | 166.07234 | 1.62 | x | x |
| abscisic acid | $C_{15}H_20O_4$ | [M+H]+ | 265.14344 | 11.05 | | |
| acetoin | $C_4H_8O_2$ | [M+H]+ | 89.05971 | 2.41 | | |
| acetophenone | $C_8H_8O$ | [M+H]+ | 121.06479 | 11.28 | x | x |
| acetylcholine | $C_7H_{16}ClNO_2$ | [M+H]+ | 146.11756 | 1.1 | x | x |
| adenine | $C_5H_5N_5$ | [M+H]+ | 136.06177 | 1.33 | x | x |
| adenosine-5-monophosphate | $C_{10}H_{14}N_5O_7P$ | [M+H]+ | 348.07036 | 1.47 | x | x |
| adipic acid | $C_6H_{10}O_4$ | [M+H]+ | 147.06519 | 5.1 | | |
| adipoylcarnitine C16 | $C_{13}H_{23}NO_6$ | [M+H]+ | 290.15981 | 6.16 | x | x |
| alanine | $C_3H_7NO_2$ | [M+H]+ | 90.05496 | 0.85 | | |
| aldosterone | $C_{21}H_{28}O_5$ | [M+H]+ | 361.20095 | 10.95 | | |
| alfa-hydroxyisobutyric acid | $C_4H_8O_3$ | [M+H]+ | 105.05462 | 2.5 | | x |
| allantoin | $C_4H_6N_4O_3$ | [M+H]+ | 159.05127 | 1 | x | x |
| allylanthranilate | $C_{10}H_{11}NO_2$ | [M+H]+ | 178.08626 | 12.27 | | |
| alpha-lactose | $C_{12}H_{22}O_{11}$ | [M-H]- | 341.10893 | 0.95 | | |
| alpha-linolenic acid | $C_{18}H_30O_2$ | [M+H]+ | 279.23186 | 15 | | |
| anandamide (17:1) | $C_{19}H_{37}NO_2$ | [M+H]+ | 312.28971 | 14.54 | | |
| anandamide (18:1) | $C_20H_{39}NO_2$ | [M+H]+ | 326.30536 | 15.04 | | x |
| arabinose/ribose | $C_5H_{10}O_5$ | [M-H]- | 149.04555 | 1.47 | | |
| arabitol/xylitol | $C_5H_{12}O_5$ | [M+H]+ | 153.07575 | 1 | | |

| Analytical standard | Chemical Formula | Ion Adduct | m/z-value | Retention Time (min) | Optimization | Validation |
|---|---|---|---|---|---|---|
| azelaic acid | $C_{9}H_{16}O_{4}$ | [M-H]- | 187.09758 | 8.83 | x | x |
| benzoic acid | $C_{7}H_{6}O_{2}$ | [M-H]- | 121.0295 | 8.8 | | |
| betaine | $C_{5}H_{11}NO_{2}$ | [M+H]+ | 118.08626 | 0.91 | | |
| B-hydroxyisovaleric acid | $C_{5}H_{10}O_{3}$ | [M-H]- | 117.05572 | 3.9 | x | x |
| Butyric acid | $C_{4}H_{8}O_{2}$ | [M+H]+ | 89.05971 | 6.4 | | |
| butyrylcarnitine | $C_{11}H_{21}NO_{4}$ | [M+H]+ | 232.15433 | 6.58 | x | x |
| chenodeoxycholic acid (CDCA) | $C_{24}H_{4}0O_{4}$ | [M-H]- | 391.28534 | 12.9 | x | x |
| cholic acid | $C_{24}H_{4}0O_{5}$ | [M-H]- | 407.2803 | 11.99 | x | x |
| choline | $C_{5}H_{14}NO$ | [M+H]+ | 104.10699 | 0.83 | | x |
| cinnamaldehyde | $C_{9}H_{8}O$ | [M+H]+ | 133.06479 | 11.58 | | |
| Citric acid | $C_{6}H_{8}O_{7}$ | [M+H]+ | 193.03428 | 1.4 | | |
| corticosterone | $C_{21}H_{3}0O_{4}$ | [M+H]+ | 347.22170 | 11.54 | | x |
| cortisol | $C_{21}H_{3}0O_{5}$ | [M+H]+ | 363.21660 | 11.17 | x | x |
| cortisone | $C_{21}H_{28}O_{5}$ | [M+H]+ | 361.20095 | 11.18 | | |
| coumaric acid | $C_{9}H_{8}O_{3}$ | [M+H]+ | 165.05462 | 10.28 | | |
| creatine | $C_{4}H_{9}N_{3}O_{2}$ | [M+H]+ | 132.0768 | 0.94 | x | x |
| creatinine | $C_{4}H_{7}N_{3}O$ | [M+H]+ | 114.0661 | 0.89 | x | x |
| cyclohexylamine | $C_{6}H_{13}N$ | [M+H]+ | 100.11208 | 3.5 | x | |
| cytidine | $C_{9}H_{13}N_{3}O_{5}$ | [M+H]+ | 244.0928 | 1.32 | | x |
| cytosine | $C_{4}H_{5}N_{3}O$ | [M+H]+ | 112.05054 | 1.5 | x | x |
| decanoyl-L-carnitine (10:0) | $C_{17}H_{33}NO_{4}$ | [M+H]+ | 316.24824 | 11.28 | | |
| Deoxycholic acid (DCA) | $C_{24}H_{4}0O_{4}$ | [M-H]- | 391.28534 | 13.05 | | x |
| deoxycorticosterone | $C_{21}H_{3}0O_{3}$ | [M+H]+ | 331.26677 | 12.15 | | |
| D-erythronic acid | $C_{4}H_{8}O_{5}$ | [M+H]+ | 137.04445 | 1 | | |
| dihydroorotic acid | $C_{5}H_{6}N_{2}O_{4}$ | [M+H]+ | 159.04003 | 1.05 | | |
| dipropyl disulfide | $C_{6}H_{14}S_{2}$ | [M+H]+ | 151.06097 | 1.5 | x | x |
| DL-2-aminoadipic acid | $C_{6}H_{11}NO_{4}$ | [M+H]+ | 162.07608 | 0.97 | | |
| DL-hexanoylcarnitine (C6) | $C_{13}H_{25}NO_{4}$ | [M+H]+ | 260.18563 | 10.14 | | |
| DL-leucyl DL-phenylalanine | $C_{15}H_{22}N_{2}O_{3}$ | [M+H]+ | 279.17031 | 7.69 | | |

| Analytical standard | Chemical Formula | Ion Adduct | m/z-value | Retention Time (min) | Optimization | Validation |
|---|---|---|---|---|---|---|
| DL-Maleic acid | $C_4H_4O_4$ | [M-H]- | 115.00368 | 1.5 | | |
| DL-methionine sulfoxide | $C_5H_{11}NO_3S$ | [M+H]+ | 166.05324 | 0.93 | | |
| dodecanedioic acid | $C_{12}H_{22}O_4$ | [M-H]- | 229.14453 | 9.44 | | x |
| dodecenoylcarnitine (12:1) | $C_{19}H_{38}NO_4$ | [M+H]+ | 342.26389 | 11.61 | | |
| dopamine | $C_8H_{11}NO_2$ | [M+H]+ | 154.08626 | 1.48 | | |
| D-pantothenic acid | $C_9H_{17}NO_5$ | [M+H]+ | 220.11795 | 6.5 | x | x |
| D-quinic acid | $C_7H_{12}O_6$ | [M+H]+ | 193.07066 | 1.06 | | |
| epinephrine | $C_9H_{13}NO_3$ | [M+H]+ | 184.09682 | 1.27 | | |
| equol | $C_{15}H_{14}O_3$ | [M+H]+ | 243.10215 | 11.3 | | |
| ethanolamine | $C_2H_7NO$ | [M+H]+ | 62.06004 | 0.79 | | |
| ethyl heptanoate | $C_9H_{18}O_2$ | [M+H]+ | 159.13796 | 13.5 | | |
| fructose/galactose | $C_6H_{12}O_6$ | [M-H]- | 179.05611 | 0.89 | x | |
| fumaric acid | $C_4H_4O_4$ | [M-H]- | 115.00368 | 1.9 | | |
| furfural | $C_5H_4O_2$ | [M+H]+ | 97.02841 | 6.2 | | |
| GABA | $C_4H_9NO_2$ | [M+H]+ | 104.07061 | 0.92 | x | x |
| gamma-butyrolactone | $C_4H_6O_2$ | [M+H]+ | 87.04406 | 2.5 | | |
| gamma-glutamylphenylalanine | $C_{14}H_{18}N_2O_5$ | [M+H]+ | 295.12884 | 8.03 | x | x |
| Glucitol | $C_6H_{14}O_6$ | [M+H]+ | 183.08631 | 1 | | |
| gluconic acid | $C_6H_{12}O_7$ | [M-H]- | 195.05103 | 0.95 | x | |
| glucose | $C_6H_{12}O_6$ | [M+Na]+ | 203.05261 | 0.91 | x | x |
| glutaric acid | $C_5H_8O_4$ | [M-H]- | 131.03498 | 3.6 | | |
| glyceric acid | $C_3H_6O_4$ | [M-H]- | 105.01779 | 1.09 | x | x |
| glycerol | $C_3H_8O_3$ | [M+H]+ | 93.05462 | 1 | x | |
| glycerolphosphorylcholine | $C_8H_20NO_6P$ | [M+H]+ | 258.11010 | 0.97 | | |
| glycoursodeoxycholic acid (GUDCA) | $C_{26}H_{43}NO_5$ | [M-H]- | 448.30685 | 12.02 | x | |
| guanine | $C_5H_5N_5O$ | [M+H]+ | 152.05669 | 1.36 | | x |
| hexadecamide | $C_{16}H_{33}NO$ | [M+H]+ | 256.26349 | 15.18 | x | x |
| hippuric acid | $C_9H_9NO_3$ | [M+H]+ | 180.06529 | 8.45 | x | x |

| Analytical standard | Chemical Formula | Ion Adduct | m/z-value | Retention Time (min) | Optimization | Validation |
|---|---|---|---|---|---|---|
| homocysteine | $C_4H_9NO_2S$ | [M+H]+ | 136.04268 | 1.13 | x | x |
| hydroquinone | $C_6H_6O_2$ | [M+H]+ | 111.04406 | 6.9 | x | |
| hypoxanthine | $C_5H_4N_4O$ | [M+H]+ | 137.04579 | 1.48 | x | x |
| imidazolepropionic acid | $C_6H_8N_2O_2$ | [M+H]+ | 141.06585 | 1.38 | | x |
| indole | $C_8H_7N$ | [M+H]+ | 118.06513 | 11.88 | | |
| isobutyrylcarnitine | $C_{11}H_{21}NO_4$ | [M+H]+ | 232.15386 | 6.48 | x | x |
| kynurenic acid | $C_{10}H_7NO_3$ | [M+H]+ | 190.04987 | 7.83 | x | x |
| kynurenine | $C_{10}H_{12}N_2O_3$ | [M+H]+ | 209.09207 | 4.55 | x | x |
| L3-phenylacetic acid | $C_8H_8O_2$ | [M+H]+ | 137.05971 | 8.89 | | |
| lactic acid | $C_3H_6O_3$ | [M-H]- | 89.0244173 | 1.43 | x | x |
| L-arginine | $C_6H_{14}N_4O_2$ | [M+H]+ | 175.11895 | 0.78 | x | x |
| L-asparagine | $C_4H_8N_2O_3$ | [M+H]+ | 133.06077 | 0.85 | | |
| L-carnitine | $C_7H_{15}NO_3$ | [M+H]+ | 162.1125 | 0.87 | | x |
| L-citrulline | $C_6H_{13}N_3O_3$ | [M+H]+ | 176.10297 | 0.97 | x | x |
| L-dehydroascorbic acid | $C_6H_6O_6$ | [M-H]- | 173.00916 | 1.16 | | x |
| leucylglycine | $C_8H_{16}N_2O_3$ | [M+H]+ | 189.12337 | 3.8 | | |
| L-glutamic acid | $C_5H_9NO_4$ | [M+H]+ | 148.06043 | 0.88 | | |
| L-glutamine | $C_5H_{10}N_2O_3$ | [M+H]+ | 147.07642 | 0.86 | x | x |
| L-gulonic acid gamma lactone | $C_6H_{10}O_6$ | [M-H]- | 177.04046 | 0.9 | | |
| L-histidine | $C_6H_9N_3O_2$ | [M+H]+ | 156.07675 | 0.85 | | |
| linoleamide | $C_{18}H_{33}NO$ | [M+H]+ | 280.26349 | 14.71 | x | x |
| L-isoleucine | $C_6H_{13}NO_2$ | [M+H]+ | 132.10191 | 2.2 | x | x |
| L-leucine | $C_6H_{13}NO_2$ | [M+H]+ | 132.10191 | 2.4 | x | x |
| L-lysine | $C_6H_{14}N_2O_2$ | [M+H]+ | 147.1128 | 0.75 | | x |
| L-methionine | $C_5H_{11}NO_2S$ | [M+H]+ | 150.05833 | 1.42 | x | x |
| L-methionine sulfone | $C_5H_{11}NO_4S$ | [M+H]+ | 182.04816 | 1.02 | | |
| L-ornithine | $C_5H_{12}N_2O_2$ | [M-H]- | 131.0826 | 0.9 | | |
| L-phenylalanine | $C_9H_{11}NO_2$ | [M+H]+ | 166.08626 | 5.09 | x | x |
| L-proline | $C_5H_9NO_2$ | [M+H]+ | 116.07061 | 0.95 | x | |

| Analytical standard | Chemical Formula | Ion Adduct | m/z-value | Retention Time (min) | Optimization | Validation |
|---|---|---|---|---|---|---|
| L-pyroglutamic acid | $C_5H_7NO_3$ | [M+H]+ | 130.04987 | 1.6 | x | x |
| L-serine | $C_3H_7NO_3$ | [M+H]+ | 106.04987 | 0.84 | | |
| L-threonine | $C_4H_9NO_3$ | [M+H]+ | 120.06552 | 0.87 | | x |
| L-tyrosine | $C_9H_{11}NO_3$ | [M+H]+ | 182.08117 | 1.83 | x | x |
| L-valine 5-aminovaleric acid | $C_5H_{11}NO_2$ | [M+H]+ | 118.08626 | 1.13 | | x |
| lyso-phosphatidylcholine (18:0) | $C_{26}H_{52}NO_7P$ | [M+H]+ | 522.35542 | 13.67 | | x |
| malic acid | $C_4H_6O_5$ | [M-H]- | 133.01424 | 1.19 | | x |
| malonylcarnitine | $C_{10}H_{17}NO_6$ | [M+H]+ | 248.11286 | 1.13 | x | x |
| mannose | $C_6H_{12}O_6$ | [M-H]- | 179.05611 | 1 | x | x |
| methyl cyclohexanecarboxylate | $C_8H_{14}O_2$ | [M+H]+ | 143.10666 | 12.56 | | |
| methyl isobutyrate | $C_5H_{10}O_2$ | [M+H]+ | 103.07536 | 11.09 | | |
| methylsuccinic acid | $C_5H_8O_4$ | [M+H]+ | 133.04954 | 0.99 | | |
| myo-inositol | $C_6H_{12}O_6$ | [M-H]- | 179.05611 | 0.86 | | |
| myristoleic acid | $C_{14}H_{26}O_2$ | [M+H]+ | 227.20056 | 14.3 | | |
| N,N-dimethylarginine | $C_8H_{18}N_4O_2$ | [M+H]+ | 203.15025 | 0.93 | x | x |
| N6-methyladenosine | $C_{11}H_{15}N_5O_4$ | [M+H]+ | 282.11968 | 4.2 | | x |
| N-acetyl galactosamine | $C_8H_{15}NO_6$ | [M+H]+ | 222.09721 | 1.05 | | |
| N-acetylagmatine | $C_7H_{16}N_4O$ | [M+H]+ | 173.13951 | 1.55 | | |
| N-acetylarginine | $C_8H_{16}N_4O_3$ | [M+H]+ | 217.12952 | 1.16 | | x |
| N-acetylasparagine | $C_6H_{10}N_2O_4$ | [M+H]+ | 175.07133 | 1.16 | x | x |
| N-acetyl-beta-alanine | $C_5H_9NO_3$ | [M+H]+ | 132.06552 | 1.68 | | x |
| N-acetylglutamic acid | $C_7H_{11}NO_5$ | [M+H]+ | 190.071 | 1.76 | x | x |
| N-acetylglycine | $C_4H_7NO_3$ | [M+H]+ | 118.04987 | 1.5 | x | |
| N-acetyl-L-methionine | $C_7H_{13}NO_3S$ | [M+H]+ | 192.06889 | 7.6 | x | x |
| N-acetyl-L-phenylalanine | $C_{11}H_{13}NO_3$ | [M+H]+ | 208.09682 | 10.17 | x | x |
| N-acetyl-L-proline | $C_7H_{11}NO_3$ | [M+H]+ | 158.08117 | 6.34 | | |
| N-acetyltryptophan | $C_{13}H_{14}N_2O_3$ | [M+H]+ | 247.1077 | 10.42 | x | x |
| N-acetyltyrosine | $C_{11}H_{13}NO_4$ | [M+H]+ | 224.09173 | 7.67 | x | x |

| Analytical standard | Chemical Formula | Ion Adduct | m/z-value | Retention Time (min) | Optimization | Validation |
|---|---|---|---|---|---|---|
| N-acetylvaline/N-isovalerylglycine | $C_7H_{13}NO_3$ | [M+H]+ | 160.09682 | 7.6 | x | x |
| nicotinamide | $C_6H_6N_2O$ | [M+H]+ | 123.05530 | 1.59 | x | x |
| nicotinic acid/picolinic acid | $C_6H_5NO_2$ | [M+H]+ | 124.0393 | 1.42 | x | |
| N-nitrosodiethylamine | $C_4H_{10}N_2O$ | [M+H]+ | 103.08659 | 2.1 | | |
| N-palmitoylglycine | $C_{18}H_{35}NO_3$ | [M+H]+ | 314.26897 | 14.47 | | |
| N-tigloylglycine | $C_7H_{11}NO_3$ | [M+H]+ | 158.08117 | 7.29 | | |
| O-Acetyl-L carnitine | $C_9H_{17}NO_4$ | [M+H]+ | 204.12303 | 1.5 | | x |
| octanoyl-L-carnitine (8:0) | $C_{15}H_{29}NO_4$ | [M+H]+ | 288.21693 | 10.89 | x | |
| oleamide | $C_{18}H_{35}NO$ | [M+H]+ | 282.27914 | 15.34 | x | x |
| oleoyl-L-carnitine | $C_{25}H_{47}NO_4$ | [M+H]+ | 426.35779 | 12.72 | | |
| ophthalmic acid | $C_{11}H_{19}N_3O_6$ | [M+H]+ | 290.134436 | 1.65 | x | |
| O-succinylcarnitine | $C_{11}H_{19}NO_6$ | [M+H]+ | 262.12851 | 1.75 | x | x |
| oxoglutaric acid | $C_5H_6O_5$ | [M+H]+ | 147.02880 | 1.4 | x | x |
| pantothenol | $C_9H_{19}NO_4$ | [M+H]+ | 206.13868 | 6.63 | x | x |
| phenyl actic acid | $C_8H_8O_2$ | [M-H]- | 135.04515 | 10.04 | x | |
| phenylacetaldehyde | $C_8H_8O$ | [M+H]+ | 121.06479 | 11 | | x |
| phenylAc-Gln-OH | $C_{13}H_{16}N_2O_4$ | [M+H]+ | 265.11828 | 8.54 | x | x |
| phosphocholine calcium salt tetrahydrate | $C_5H_{13}CaClNO_4P{\cdot}4H_2O$ | [M+H]+ | 184.07339 | 0.94 | x | |
| pimelic acid | $C_7H_{12}O_4$ | [M-H]- | 159.06628 | 6.5 | x | |
| pipecolic acid | $C_6H_{11}NO_2$ | [M+H]+ | 130.08626 | 1.4 | x | |
| piperine | $C_{17}H_{19}NO_3$ | [M+H]+ | 286.14377 | 12.34 | | |
| propionyl carnitine C3 | $C_{10}H_{19}NO_4$ | [M+H]+ | 218.13868 | 3.01 | | x |
| putrescine | $C_4H_{12}N_2$ | [M+H]+ | 89.10732 | 0.75 | x | |
| pyridine | $C_5H_5N$ | [M+H]+ | 80.04948 | 1 | | |
| pyrrole-2-carboxylic acid | $C_5H_5NO_2$ | [M+H]+ | 112.03930 | 7.14 | x | |
| pyruvic acid | $C_3H_4O_3$ | [M-H]- | 87.00877 | 1.16 | x | x |
| rac-glycerol 1-phosphate sodium salt hydrate | $C_3H_9O_6P$ | [M+H]+ | 173.02095 | 1 | x | |

| Analytical standard | Chemical Formula | Ion Adduct | m/z-value | Retention Time (min) | Optimization | Validation |
|---|---|---|---|---|---|---|
| saccharic acid | $C_6H_{10}O_8$ | [M-H]- | 209.03029 | 1.04 | x | x |
| S-adenosylhomocystein | $C_{14}H_20N_6O_5S$ | [M+H]+ | 385.12886 | 2.13 | x | |
| sarcosine | $C_3H_7NO_2$ | [M+H]+ | 90.05496 | 0.85 | | |
| sebacic acid | $C_{10}H_{18}O_4$ | [M-H]- | 201.11323 | 9.03 | x | x |
| serotonin | $C_{10}H_{12}N_2O$ | [M+H]+ | 177.10224 | 3.7 | | |
| sinapic acid | $C_{11}H_{12}O_5$ | [M-H]- | 223.0612 | 10.2 | | |
| sodium glycochenodeoxycholate (GCDCA) | $C_{26}H_{42}NNaO_5$ | [M-H]- | 391.28534 | 12.03 | | |
| sodium glycocholate (GCA) | $C_{26}H_{42}NNaO_6$ | [M-H]- | 464.30176 | 11.47 | | |
| sodium glycodeoxycholate (GDCA) | $C_{26}H_{42}NO_5Na$ | [M-H]- | 448.30685 | 12.18 | x | |
| sodium taurocholate (TCA) | $C_{26}H_{44}NNaO_7S$ | [M-H]- | 514.28440 | 11.26 | x | x |
| sodium taurodeoxycholate hydrate (TDCA) | $C_{26}H_{44}NO_6SNa$ | [M-H]- | 498.28948 | 11.78 | | |
| spermidine | $C_7H_{19}N_3$ | [M+H]+ | 146.16517 | 0.75 | x | x |
| spermine | $C_{10}H_{26}N_4$ | [M+H]+ | 203.22302 | 0.7 | | |
| stearoyl-L-carnitine | $C_{25}H_{49}NO_4$ | [M+H]+ | 428.37240 | 13.09 | | |
| succinic acid | $C_4H_6O_4$ | [M-H]- | 117.01933 | 1.96 | x | x |
| sucrose | $C_{12}H_{22}O_{11}$ | [M-H]- | 341.10893 | 1.02 | | |
| syringic acid | $C_9H_{10}O_5$ | [M+H]+ | 199.0601 | 7 | | |
| taurine | $C_2H_7NO_3S$ | [M+H]+ | 126.02190 | 0.93 | | |
| taurochenodeoxycholic acid sodium salt (TCDCA) | $C_{26}H_{44}NO_6SNa$ | [M-H]- | 498.28948 | 11.65 | | |
| testosterone | $C_{19}H_{28}O_2$ | [M+H]+ | 289.21621 | 12.47 | | |
| tetradecanoylcarnitine (14:0) | $C_{21}H_{41}NO_4$ | [M+H]+ | 372.31084 | 12.24 | | |
| thiabendazole | $C_{10}H_7N_3S$ | [M+H]+ | 202.04334 | 8.4 | | |
| thiazolidine-4-carboxylic acid | $C_4H_7NO_2S$ | [M+H]+ | 134.02703 | 1 | | |
| threonic acid | $C_4H_8O_5$ | [M+H]+ | 137.04445 | 0.95 | | x |
| thymine | $C_5H_6N_2O_2$ | [M+H]+ | 127.0502 | 2.6 | | |
| tolbutamide | $C_{12}H_{18}N_2O_3S$ | [M+H]+ | 271.11110 | 11.75 | | |

| **Analytical standard** | **Chemical Formula** | **Ion Adduct** | **m/z-value** | **Retention Time (min)** | **Optimization** | **Validation** |
|---|---|---|---|---|---|---|
| trans, trans-2,4decadienal | $C_{10}H_{16}O$ | [M+H]+ | 153.12739 | 13.27 | | |
| trans-2-octenoylcarnitine | $C_{15}H_{27}NO_4$ | [M+H]+ | 286.20128 | 10.81 | | |
| trans-3-octen-2-one | $C_8H_{14}O$ | [M+H]+ | 127.11174 | 12.16 | | |
| trans-4-hydroxy-l-proline | $C_5H_9NO_3$ | [M+H]+ | 132.06552 | 0.9 | | |
| trans-hexadec-2-enoyl carnitine | $C_{23}H_{43}NO_4$ | [M+H]+ | 398.32549 | 12.44 | | |
| trehalose | $C_{12}H_{22}O_{11}$ | [M+H]+ | 360.15004 | 1.04 | x | |
| Triethanolamine hydrochloride | $C_6H_{16}ClNO_3$ | [M+H]+ | 150.11247 | 0.87 | | |
| tryptamine | $C_{10}H_{12}N_2$ | [M+H]+ | 161.10732 | 7.97 | | |
| tryptophan | $C_{11}H_{12}N_2O_2$ | [M+H]+ | 205.09715 | 7.5 | x | x |
| tyramine | $C_8H_{11}NO$ | [M+H]+ | 138.09134 | 2.35 | | |
| UDCA | $C_{24}H_40O_4$ | [M-H]- | 391.28538 | 12.09 | | |
| uracil | $C_4H_4N_2O_2$ | [M+H]+ | 113.03455 | 1.42 | x | |
| Uridine | $C_9H_{12}N_2O_6$ | [M+H]+ | 245.07681 | 1.47 | | |
| urocanic acid | $C_6H_6N_2O_2$ | [M+H]+ | 139.0502 | 1.41 | x | |
| valerylcarnitine | $C_{12}H_{23}NO_4$ | [M+H]+ | 246.16940 | 8.2 | x | x |
| vanillic acid | $C_8H_8O_4$ | [M+H]+ | 169.04954 | 8.7 | | |
| L-valine/aminovaleric acid | $C_5H_{11}NO_2$ | [M+H]+ | 118.08626 | 1.1 | | x |

**Table S8: Instrumental parameters for UHPLC-HRMS analysis**

| **Instrumental parameters** | **Settings** |
|---|---|
| mass spectrometer | Q-Exactive™ stand-alone bench top quadrupole-Orbitrap high-resolution mass spectrometer ((Thermo FisherScientific, San José, CA, USA) |
| liquid chromatographer | Dionex UltiMate 3000 XRS UHPLC system (Thermo FisherScientific, San José, CA, USA) |
| ionization source/mode | HESI-II/ positive and negative |
| column type | Acquity HSS T3 C18 column (1.8µm, 150 x 2.1mm) |
| column temperature | 45°C |
| flow rate | 0.4 ml $min^{-1}$ |
| binary solvent | UPW (A) and acetonitrile (B) with 0.1 % formic acid |
| gradient profile (v/v of solvent A) | 0−1.5 min at 98 %, 1.5−7.0 min from 98 % to 75 %, 7.0−8.0 min from 75 % to 40 %, 8.0−12.0 min from 40 % to 5 %, 12.0−14.0 min at 5 %, 14.0−14.1 min from 5 % to 98 %; 4 min re-equilibration |
| injection volume | 10 µl |
| sheath gas flow rate | 50 a.u. |
| auxiliary gas flow rate | 25 a.u. |
| sweep gas flow rate | 5 a.u. |
| heater temperature | 350°C |
| capillary temperature | 250°C |
| S-lens RF level | 50% |
| spray voltage | 3 kV (pos) and 2 kV (neg) |
| *m/z* scan range | 53-800 Da |
| automatic gain control target | 1 x 106 ions |
| maximum injection time | 70 ms |
| acquisition mode | full scan |
| mass resolution settings | 140 000 FWHM (1 Hz) |

a.u.: arbitrary units, UHPLC: ultrahigh performance liquid chromatography, UPW: ultrapure water, FWHM: full width half maximum

**Table S9: List of the targeted metabolites (n = 101) in the adult dog cohort for comparison of IE with non-brain related diseases.**

| **Targeted metabolite** | **Chemical Formula** | **Ion Adduct** | **m/z-value** | **Retention Time (min)** |
|---|---|---|---|---|
| 1,3-propanediol | $C_3H_8O_2$ | [M+H]+ | 77.05971 | 1.33 |
| 2-hydroxyhexanoic acid | $C_6H_{12}O_3$ | [M-H]- | 131.0695 | 9.4 |
| 2-hydroxyisocaproic acid | $C_6H_{12}O_3$ | [M+H]+ | 131.06995 | 9.16 |
| 2-hydroxy 3-methylvalerate | $C_6H_{12}O_3$ | [M-H]- | 131.06950 | 9.03 |
| 2-methylbutyryl-L-carnitine | $C_{12}H_{23}NO_4$ | [M+H]+ | 246.16998 | 8.55 |
| 2-phenylethylamine | $C_8H_{11}N$ | [M+H]+ | 122.09643 | 6.59 |
| 2-piperidinone | $C_5H_9NO$ | [M+H]+ | 100.07569 | 5 |
| 3-indoleacetic acid | $C_10H_9NO_2$ | [M+H]+ | 176.07061 | 8.84 |
| 3-methoxytyramine hydroxychloride | $C_9H_{13}NO_2$ | [M+H]+ | 168.10191 | 4.4 |
| 3-methylbutyryl/isovalaryl-L-carnitine | $C_{12}H_{23}NO_4$ | [M+H]+ | 246.16998 | 8.21 |
| 4-guanidinobutyric acid | $C_5H_{11}N_3O_2$ | [M+H]+ | 146.09220 | 1.44 |
| 4-hydroxyphenylpyruvic acid | $C_9H_8O_4$ | [M-H]- | 179.03498 | 4.55 |
| 4-methyl-2-oxovaleric acid | $C_6H_10O_3$ | [M-H]- | 129.05432 | 8.41 |
| 5-hydroxyindole-3-acetic acid | $C_10H_9NO_3$ | [M+H]+ | 192.06552 | 8.04 |
| 6-acetyl-L-lysine | $C_8H_{16}N_2O_3$ | [M+H]+ | 189.12337 | 1.47 |
| 7-methylguanine | $C_6H_7N_5O$ | [M+H]+ | 166.07234 | 1.62 |
| acetophenone | $C_8H_8O$ | [M+H]+ | 121.06479 | 11.28 |
| acetylcholine | $C_7H_{16}ClNO_2$ | [M+H]+ | 146.11756 | 1.1 |
| adenine | $C_5H_5N_5$ | [M+H]+ | 136.06177 | 1.33 |
| adenosine-5-monophosphate | $C_10H_{14}N_5O_7P$ | [M+H]+ | 348.07036 | 1.47 |
| adipoylcarnitine C16 | $C_{13}H_{23}NO_6$ | [M+H]+ | 290.15981 | 6.16 |
| alfa-hydroxyisobutyric acid | $C_4H_8O_3$ | [M+H]+ | 105.05462 | 2.5 |
| allantoin | $C_4H_6N_4O_3$ | [M+H]+ | 159.05127 | 1 |
| anandamide (18:1) | $C_20H_{39}NO_2$ | [M+H]+ | 326.30536 | 15.04 |
| azelaic acid | $C_9H_{16}O_4$ | [M-H]- | 187.09758 | 8.83 |

| | | | | |
|---|---|---|---|---|
| B-hydroxyisovaleric acid | $C_{5}H_{1}0O_{3}$ | [M-H]- | 117.05572 | 3.9 |
| butyrylcarnitine | $C_{11}H_{21}NO_{4}$ | [M+H]+ | 232.15433 | 6.58 |
| chenodeoxycholic acid (CDCA) | $C_{24}H_{4}0O_{4}$ | [M-H]- | 391.28534 | 12.9 |
| cholic acid | $C_{24}H_{4}0O_{5}$ | [M-H]- | 407.2803 | 11.99 |
| choline | $C_{5}H_{14}NO$ | [M+H]+ | 104.10699 | 0.83 |
| corticosterone | $C_{21}H_{3}0O_{4}$ | [M+H]+ | 347.22170 | 11.54 |
| cortisol | $C_{21}H_{3}0O_{5}$ | [M+H]+ | 363.21660 | 11.17 |
| creatine | $C_{4}H_{9}N_{3}O_{2}$ | [M+H]+ | 132.0768 | 0.94 |
| creatinine | $C_{4}H_{7}N_{3}O$ | [M+H]+ | 114.0661 | 0.89 |
| cytidine | $C_{9}H_{13}N_{3}O_{5}$ | [M+H]+ | 244.0928 | 1.32 |
| cytosine | $C_{4}H_{5}N_{3}O$ | [M+H]+ | 112.05054 | 1.5 |
| Deoxycholic acid (DCA) | $C_{24}H_{4}0O_{4}$ | [M-H]- | 391.28534 | 13.05 |
| dipropyl disulfide | $C_{6}H_{14}S_{2}$ | [M+H]+ | 151.06097 | 1.5 |
| dodecanedioic acid | $C_{12}H_{22}O_{4}$ | [M-H]- | 229.14453 | 9.44 |
| D-pantothenic acid | $C_{9}H_{17}NO_{5}$ | [M+H]+ | 220.11795 | 6.5 |
| GABA | $C_{4}H_{9}NO_{2}$ | [M+H]+ | 104.07061 | 0.92 |
| gamma-glutamylphenylalanine | $C_{14}H_{18}N_{2}O_{5}$ | [M+H]+ | 295.12884 | 8.03 |
| glucose | $C_{6}H_{12}O_{6}$ | [M+Na]+ | 203.05261 | 0.91 |
| glyceric acid | $C_{3}H_{6}O_{4}$ | [M-H]- | 105.01779 | 1.09 |
| hexadecamide | $C_{16}H_{33}NO$ | [M+H]+ | 256.26349 | 15.18 |
| hippuric acid | $C_{9}H_{9}NO_{3}$ | [M+H]+ | 180.06529 | 8.45 |
| homocysteine | $C_{4}H_{9}NO_{2}S$ | [M+H]+ | 136.04268 | 1.13 |
| hypoxanthine | $C_{5}H_{4}N_{4}O$ | [M+H]+ | 137.04579 | 1.48 |
| imidazolepropionic acid | $C_{6}H_{8}N_{2}O_{2}$ | [M+H]+ | 141.06585 | 1.38 |
| isobutyrylcarnitine | $C_{11}H_{21}NO_{4}$ | [M+H]+ | 232.15386 | 6.48 |
| kynurenic acid | $C_{1}0H_{7}NO_{3}$ | [M+H]+ | 190.04987 | 7.83 |
| kynurenine | $C_{1}0H_{12}N_{2}O_{3}$ | [M+H]+ | 209.09207 | 4.55 |
| lactic acid | $C_{3}H_{6}O_{3}$ | [M-H]- | 89.0244173 | 1.43 |
| L-arginine | $C_{6}H_{14}N_{4}O_{2}$ | [M+H]+ | 175.11895 | 0.78 |
| L-carnitine | $C_{7}H_{15}NO_{3}$ | [M+H]+ | 162.1125 | 0.87 |

| | | | | |
|---|---|---|---|---|
| L-citrulline | $C_6H_{13}N_3O_3$ | [M+H]+ | 176.10297 | 0.97 |
| L-dehydroascorbic acid | $C_6H_6O_6$ | [M-H]- | 173.00916 | 1.16 |
| L-glutamine | $C_5H_10N_2O_3$ | [M+H]+ | 147.07642 | 0.86 |
| linoleamide | $C_{18}H_{33}NO$ | [M+H]+ | 280.26349 | 14.71 |
| L-isoleucine | $C_6H_{13}NO_2$ | [M+H]+ | 132.10191 | 2.2 |
| L-leucine | $C_6H_{13}NO_2$ | [M+H]+ | 132.10191 | 2.4 |
| L-lysine | $C_6H_{14}N_2O_2$ | [M+H]+ | 147.1128 | 0.75 |
| L-methionine | $C_5H_{11}NO_2S$ | [M+H]+ | 150.05833 | 1.42 |
| L-phenylalanine | $C_9H_{11}NO_2$ | [M+H]+ | 166.08626 | 5.09 |
| L-pyroglutamic acid | $C_5H_7NO_3$ | [M+H]+ | 130.04987 | 1.6 |
| L-threonine | $C_4H_9NO_3$ | [M+H]+ | 120.06552 | 0.87 |
| L-tyrosine | $C_9H_{11}NO_3$ | [M+H]+ | 182.08117 | 1.83 |
| L-valine 5-aminovaleric acid | $C_5H_{11}NO_2$ | [M+H]+ | 118.08626 | 1.13 |
| lyso-phosphatidylcholine (18:0) | $C_{26}H_{52}NO_7P$ | [M+H]+ | 522.35542 | 13.67 |
| malic acid | $C_4H_6O_5$ | [M-H]- | 133.01424 | 1.19 |
| malonylcarnitine | $C_10H_{17}NO_6$ | [M+H]+ | 248.11286 | 1.13 |
| mannose | $C_6H_{12}O_6$ | [M-H]- | 179.05611 | 1 |
| N,N-dimethylarginine | $C_8H_{18}N_4O_2$ | [M+H]+ | 203.15025 | 0.93 |
| N6-methyladenosine | $C_{11}H_{15}N_5O_4$ | [M+H]+ | 282.11968 | 4.2 |
| N-acetylarginine | $C_8H_{16}N_4O_3$ | [M+H]+ | 217.12952 | 1.16 |
| N-acetylasparagine | $C_6H_10N_2O_4$ | [M+H]+ | 175.07133 | 1.16 |
| N-acetyl-beta-alanine | $C_5H_9NO_3$ | [M+H]+ | 132.06552 | 1.68 |
| N-acetylglutamic acid | $C_7H_{11}NO_5$ | [M+H]+ | 190.071 | 1.76 |
| N-acetyl-L-methionine | $C_7H_{13}NO_3S$ | [M+H]+ | 192.06889 | 7.6 |
| N-acetyl-L-phenylalanine | $C_{11}H_{13}NO_3$ | [M+H]+ | 208.09682 | 10.17 |
| N-acetyltryptophan | $C_{13}H_{14}N_2O_3$ | [M+H]+ | 247.1077 | 10.42 |
| N-acetyltyrosine | $C_{11}H_{13}NO_4$ | [M+H]+ | 224.09173 | 7.67 |
| N-acetylvaline/N-isovalerylglycine | $C_7H_{13}NO_3$ | [M+H]+ | 160.09682 | 7.6 |
| nicotinamide | $C_6H_6N_2O$ | [M+H]+ | 123.05530 | 1.59 |
| O-Acetyl-L carnitine | $C_9H_{17}NO_4$ | [M+H]+ | 204.12303 | 1.5 |

| | | | | |
|---|---|---|---|---|
| oleamide | $C_{18}H_{35}NO$ | [M+H]+ | 282.27914 | 15.34 |
| O-succinylcarnitine | $C_{11}H_{19}NO_6$ | [M+H]+ | 262.12851 | 1.75 |
| oxoglutaric acid | $C_5H_6O_5$ | [M+H]+ | 147.02880 | 1.4 |
| pantothenol | $C_9H_{19}NO_4$ | [M+H]+ | 206.13868 | 6.63 |
| phenylacetaldehyde | $C_8H_8O$ | [M+H]+ | 121.06479 | 11 |
| phenylAc-Gln-OH | $C_{13}H_{16}N_2O_4$ | [M+H]+ | 265.11828 | 8.54 |
| propionyl carnitine C3 | $C_{1}0H_{19}NO_4$ | [M+H]+ | 218.13868 | 3.01 |
| pyruvic acid | $C_3H_4O_3$ | [M-H]- | 87.00877 | 1.16 |
| saccharic acid | $C_6H_{1}0O_8$ | [M-H]- | 209.03029 | 1.04 |
| sebacic acid | $C_{1}0H_{18}O_4$ | [M-H]- | 201.11323 | 9.03 |
| sodium taurocholate (TCA) | $C_{26}H_{44}NNaO_7S$ | [M-H]- | 514.28440 | 11.26 |
| spermidine | $C_7H_{19}N_3$ | [M+H]+ | 146.16517 | 0.75 |
| succinic acid | $C_4H_6O_4$ | [M-H]- | 117.01933 | 1.96 |
| threonic acid | $C_4H_8O_5$ | [M+H]+ | 137.04445 | 0.95 |
| tryptophan | $C_{11}H_{12}N_2O_2$ | [M+H]+ | 205.09715 | 7.5 |
| valerylcarnitine | $C_{12}H_{23}NO_4$ | [M+H]+ | 246.16940 | 8.2 |

**Figure S1: Boxplot illustrating the iQC normalized peak areas for acetophenone in the control and IE group.**

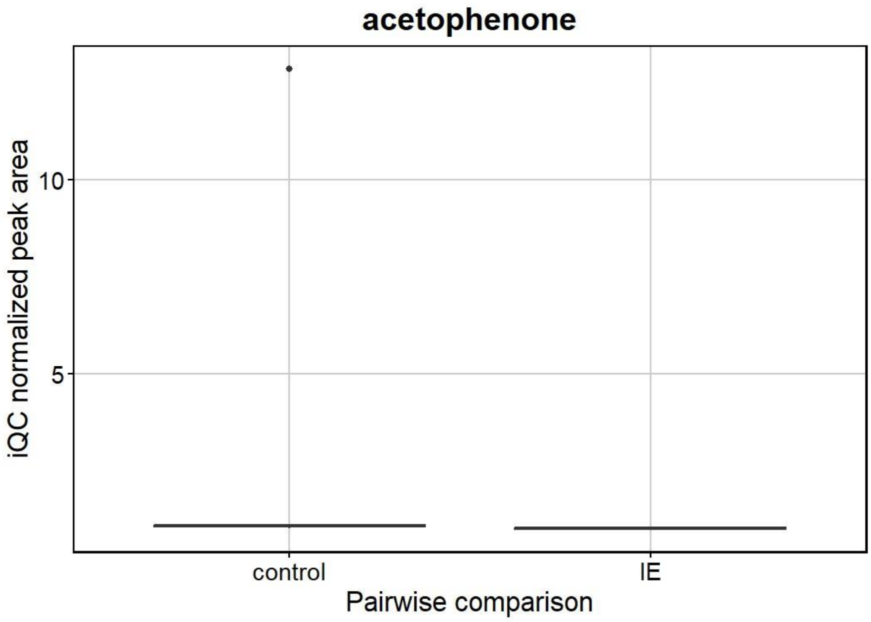


**Figure S2: Fingerprint of combined positive and negative component ions upon UHPLC-HRMS analysis of canine cerebrospinal fluid (n = 15). The putatively identified metabolite (norepinephrine) is indicated in red.**

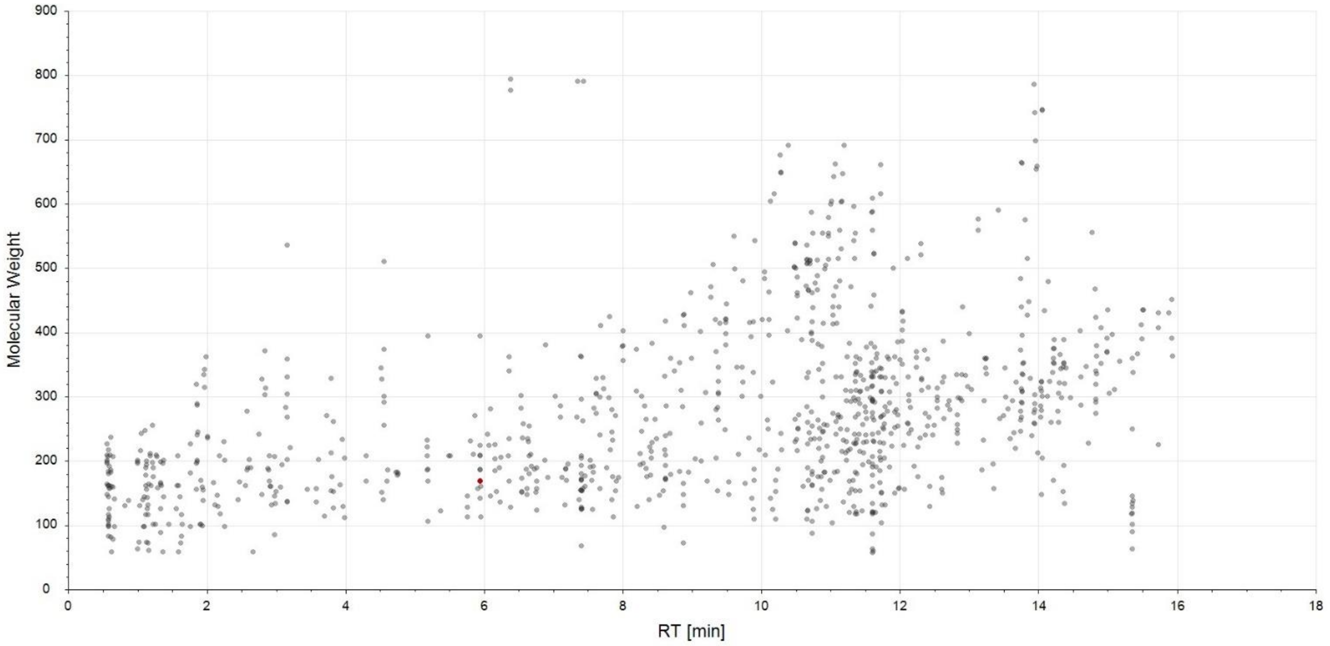

**Figure S3: Scatterplot illustrating the iQC normalized peak areas for cortisol on the y-axis and number of days between sampling and the last seizure event on the x-axis.**

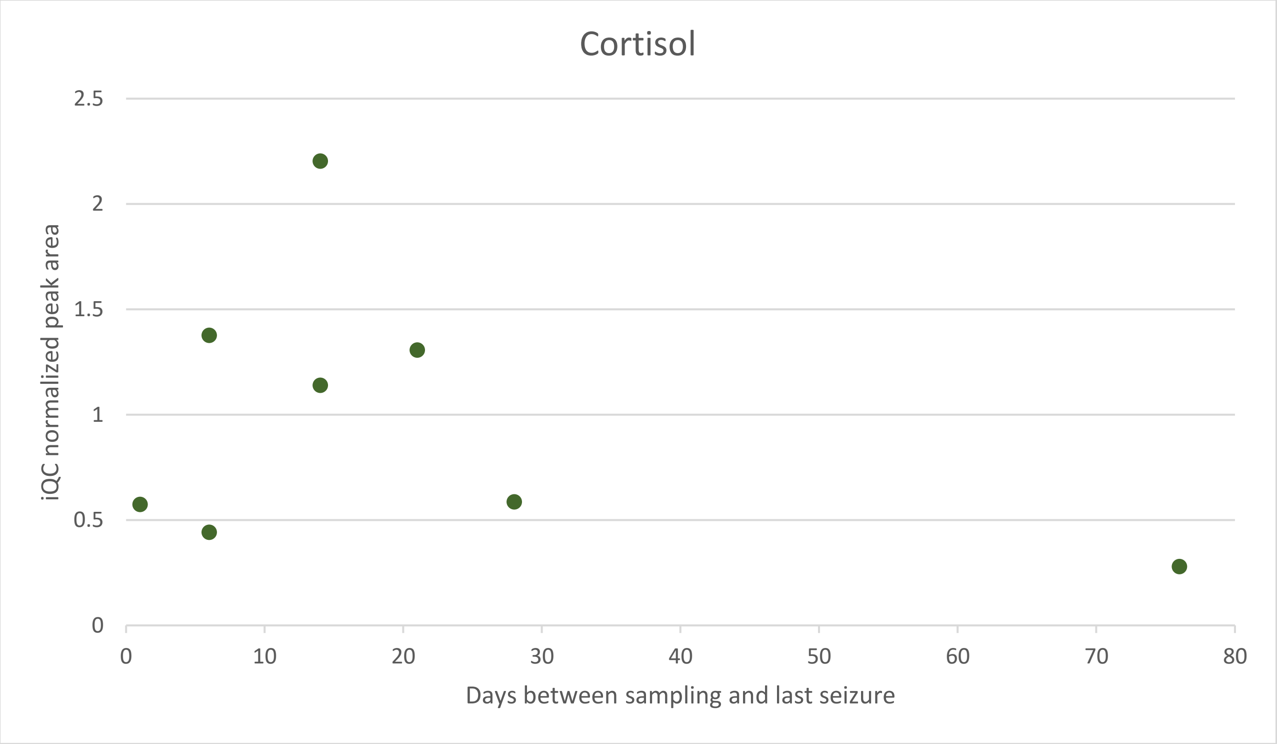

Supplement: Supplementary file 1 — Supplementary Information. [file 41598_2024_64777_MOESM1_ESM.pdf]
