# Supplementary material for: Correction: Towards a better understanding of idiopathic epilepsy through metabolic fingerprinting of cerebrospinal fluid in dogs
Source: Sci Rep. 2026 Apr 28;16:13644. doi: 10.1038/s41598-026-48734-6 (PMC13125199; doi:10.1038/s41598-026-48734-6)

Supplementary information:
TOWARDS A BETTER UNDERSTANDING OF IDIOPATHIC EPILEPSY THROUGH METABOLIC FINGERPRINTING OF CEREBROSPINAL FLUID IN DOGS

Fien Verdoodt [a,b,c], Sofie F.M. Bhatti [b] , Karla Kragic [a], Luc Van Ham [b], Lynn Vanhaecke [c],

Myriam Hesta [a,1], Lieselot Y. Hemeryck [c,1,*]

Author affiliations:

[a] *Equine and Companion Animal Nutrition, Department of Morphology, Imaging, Orthopedics, Rehabilitation and Nutrition, Faculty of Veterinary Medicine, Ghent University, Salisburylaan 133, 9820 Merelbeke, Belgium – Fien.Verdoodt@Ugent.be, Myriam.Hesta@Ugent.be*
[b] *Small Animal Department, Faculty of Veterinary Medicine, Ghent University, Salisburylaan 133, 9820 Merelbeke, Belgium – Sofie.Bhatti@Ugent.be, Luc.VanHam@Ugent.be*
[c] *Laboratory of Integrative Metabolomics, Department of Translational Physiology, Infectiology and Public Health, Faculty of Veterinary Medicine, Ghent University, Salisburylaan 133, 9820 Merelbeke Belgium – LieselotY.Hemeryck@Ugent.be, Lynn.Vanhaecke@Ugent.be*

*** Corresponding author: LieselotY.Hemeryck@Ugent.be**

[1]These authors contributed equally to this work

Tables S1: Table showing the obtained *P*-value for each targeted metabolite as evaluated in the FFD.

***P*-values FFD**	**Startvolume (100 µl / 200 µl)**	**Filter (yes / no)**	**Centrifuge time (5 min / 10 min)**	**Solvent (A / B)**
Total untargeted compounds detected	1.0E-05	4.5E-03	> 0.05	> 0.05
Targeted metabolites				
1,3-propanediol	1.1E-04	> 0.05	> 0.05	> 0.05
1-methyl-L-histidine	1.7E-02	> 0.05	> 0.05	> 0.05
2,6-diaminopimelic acid	> 0.05	> 0.05	> 0.05	> 0.05
2-hydroxyhexanoic acid	0.0E+00	> 0.05	1.4E-03	> 0.05
2-hydroxyisocaproic acid	0.0E+00	> 0.05	2.2E-04	4.1E-02
2-piperidinone	0.0E+00	> 0.05	1.6E-04	1.3E-04
3,4-dihydroxyphenylpropionic acid	1.1E-04	> 0.05	> 0.05	> 0.05
3-hydroxybutyric acid	0.0E+00	> 0.05	1.1E-02	> 0.05
3-indoleacetic acid	1.0E-05	> 0.05	> 0.05	3.0E-05
3-methylbutyryl-L-carnitine	2.0E-05	> 0.05	> 0.05	> 0.05
3-phenyl-1-propanol	3.7E-03	> 0.05	> 0.05	> 0.05
3-phenylpropionic acid	2.7E-02	> 0.05	> 0.05	3.9E-02
4-guanidinobutyric acid	1.1E-02	> 0.05	4.6E-02	> 0.05
4-methyl-2-oxovaleric acid	0.0E+00	> 0.05	4.1E-04	6.1E-04
5-hydroxyindole-3-acetic acid	0.0E+00	1.5E-02	> 0.05	1.3E-04
6-deoxyhexose	3.9E-02	> 0.05	> 0.05	1.1E-03
7-methylguanine	0.0E+00	2.4E-02	9.0E-05	3.0E-05
acetophenone	0.0E+00	5.9E-03	3.2E-04	8.1E-03
acetylcholine	1.0E-05	> 0.05	> 0.05	1.4E-03
adenine	0.0E+00	> 0.05	7.9E-04	0.0E+00
adenosine-5-monophosphate	3.8E-02	> 0.05	> 0.05	> 0.05
adipoylcarnitine	0.0E+00	> 0.05	> 0.05	> 0.05
allantoin	3.4E-02	> 0.05	3.0E-02	> 0.05
azelaic acid	> 0.05	2.5E-03	> 0.05	2.6E-02
butyrylcarnitine	0.0E+00	> 0.05	> 0.05	> 0.05
CDCA	3.0E-05	> 0.05	> 0.05	> 0.05
cholic acid	1.0E-05	> 0.05	> 0.05	> 0.05
cortisol	> 0.05	> 0.05	> 0.05	> 0.05
creatine	0.0E+00	> 0.05	2.2E-03	> 0.05
creatinine	0.0E+00	> 0.05	4.1E-03	> 0.05
cyclohexylamine	2.0E-05	> 0.05	> 0.05	0.0E+00
cytosine	0.0E+00	> 0.05	7.7E-03	> 0.05
fructose/galactose	0.0E+00	> 0.05	> 0.05	> 0.05
gluconic acid	6.4E-03	> 0.05	4.2E-02	> 0.05
glucose	0.0E+00	> 0.05	2.1E-04	> 0.05
dipropyl disulfide	0.0E+00	1.2E-02	2.0E-05	> 0.05
maltose/sucrose				

dopamine	0.0E+00	> 0.05	2.7E-02	1.4E-02
pantothenic acid	0.0E+00	> 0.05	8.0E-05	> 0.05
trehalose	0.0E+00	> 0.05	6.1E-03	1.4E-03
GABA	0.0E+00	> 0.05	1.1E-03	> 0.05
gamma-glutamylphenylalanine	0.0E+00	> 0.05	4.6E-02	> 0.05
glyceric acid	0.0E+00	> 0.05	> 0.05	> 0.05
glycerol	2.5E-03	> 0.05	> 0.05	> 0.05
glycoursodeoxycholic acid (GUDCA)	1.0E-05	> 0.05	> 0.05	> 0.05
hexadecamide	> 0.05	1.0E-05	> 0.05	> 0.05
hippuric acid	0.0E+00	> 0.05	2.5E-03	> 0.05
homocysteine	0.0E+00	3.4E-03	3.8E-04	2.6E-04
hydroquinone	0.0E+00	4.0E-05	3.2E-04	> 0.05
hypoxanthine	0.0E+00	> 0.05	1.1E-03	4.3E-04
isobutyrylcarnitine	0.0E+00	> 0.05	> 0.05	> 0.05
kynurenic acid	0.0E+00	> 0.05	7.1E-03	> 0.05
kynurenine	0.0E+00	> 0.05	7.4E-04	1.8E-02
lactic acid	0.0E+00	2.4E-02	1.1E-04	5.5E-02
arginine	0.0E+00	> 0.05	6.9E-03	> 0.05
citrulline	2.1E-04	2.9E-02	1.5E-02	> 0.05
glutamine	0.0E+00	> 0.05	6.0E-03	> 0.05
linoleamide	> 0.05	2.0E-05	> 0.05	9.0E-03
isoleucine	0.0E+00	> 0.05	2.2E-04	> 0.05
leucine	0.0E+00	> 0.05	1.0E-05	> 0.05
methionine	0.0E+00	4.3E-02	3.0E-05	> 0.05
phenylalanine	0.0E+00	> 0.05	7.3E-04	> 0.05
proline	0.0E+00	> 0.05	2.4E-02	> 0.05
pyroglutamic acid	> 0.05	> 0.05	> 0.05	> 0.05
tyrosine	0.0E+00	3.4E-02	1.3E-04	> 0.05
malonylcarnitine	0.0E+00	> 0.05	> 0.05	> 0.05
N,N-dimethylarginine	6.0E-05	> 0.05	1.5E-02	8.0E-03
N6-acetyl-L-lysine	0.0E+00	> 0.05	> 0.05	> 0.05
N-acetylasparagine	> 0.05	> 0.05	> 0.05	5.3E-03
N-acetylglutamic acid	> 0.05	> 0.05	3.4E-02	3.5E-02
N-acetylglycine	6.7E-03	1.7E-02	2.3E-02	6.4E-03
N-acetyl-L-methionine	0.0E+00	> 0.05	4.4E-03	2.3E-04
N-acetyl-L-phenylalanine	2.0E-05	> 0.05	> 0.05	2.0E-05
N-acetyltryptophan	3.0E-02	6.6E-03	> 0.05	0.0E+00
N-acetyltyrosine	0.0E+00	> 0.05	> 0.05	1.4E-02
nicotinamide	0.0E+00	> 0.05	3.7E-04	> 0.05
nicotinic acid	0.0E+00	> 0.05	5.9E-04	> 0.05
N-isovalerylglycine	0.0E+00	> 0.05	8.8E-04	4.4E-03
octanoyl-L-carnitine	3.2E-02	> 0.05	2.8E-02	1.0E-05
oleamide	> 0.05	2.5E-04	> 0.05	> 0.05
opthalmic acid	5.4E-03	> 0.05	> 0.05	> 0.05
O-succinylcarnitine	6.4E-03	> 0.05	> 0.05	> 0.05

oxoglutaric acid	> 0.05	> 0.05	4.7E-02	4.6E-04
pantothenol	> 0.05	> 0.05	> 0.05	> 0.05
phenylacetic acid	2.0E-05	> 0.05	> 0.05	> 0.05
phenylAc-GLN-OH	9.4E-04	> 0.05	> 0.05	> 0.05
phosphocholine	> 0.05	> 0.05	> 0.05	> 0.05
pimelic acid	0.03479 ^	1.3E-03	> 0.05	> 0.05
pipecolic acid	0.0E+00	2.4E-02	2.6E-04	3.2E-03
putrescine	1.7E-04	> 0.05	> 0.05	3.1E-04
pyrrole-2-carboxylic acid	0.0E+00	7.6E-02	> 0.05	> 0.05
pyruvic acid	0.0E+00	> 0.05	> 0.05	1.0E-05
rac-glycerol-1-phosphate sodium salt hydrate	2.7E-02	3.4E-02	> 0.05	> 0.05
saccharic acid	1.9E-04	> 0.05	> 0.05	5.7E-04
S-adenosylhomocystein	2.5E-04	> 0.05	> 0.05	> 0.05
sebacic acid	3.0E-05	2.8E-03	8.5E-03	6.5E-04
sodium glycodeoxycholate (GDCA)	2.0E-05	> 0.05	> 0.05	> 0.05
sodium taurocholate (TCA)	0.0E+00	2.0E-02	> 0.05	> 0.05
spermidine	3.5E-03	> 0.05	> 0.05	> 0.05
succinic acid	0.0E+00	4.2E-04	1.9E-03	2.1E-04
tryptophan	0.0E+00	> 0.05	5.4E-03	> 0.05
uracil	0.0E+00	> 0.05	2.7E-02	1.4E-02
urocanic acid	> 0.05	1.6E-02	> 0.05	5.4E-02
valerylcarnitine	0.0E+00	> 0.05	> 0.05	> 0.05

Table S2: Table showing the obtained *P*-values for the selection of targeted metabolites as evaluated in the RSM.

***P*-values RSM**	**starting V (100 - 400 µl)**	**centrifuge t (5 - 15 min)**	**centrifuge v (5000 xg - 15 000 xg)**
targeted metabolome coverage	0.0211	0.0946	0.0930
untargeted metabolome coverage	0.0007	0.3170	0.9852
butyric acid	0.0265	0.4524	0.5338
pyrrole-2-carboxylic acid	0.0001	0.3808	0.7946
indole-3-acetic acid	0.0002	0.9174	0.5982
L-glutamine	0.0002	0.4514	0.9674
glutamic acid	0.0175	0.7814	0.9776
glucose	0.0001	0.4551	0.5581
GABA	0.0003	0.4183	0.7844
tyrosine	0.0001	0.2482	0.7170
dopamine	0.0004	0.1470	0.8176
cortisol	0.0004	0.8177	0.4560
kynurenine	0.0001	0.9531	0.3559
tryptophan	0.0001	0.9668	0.6207
kynurenic acid	0.0001	0.6226	0.4096
**Effect**	*positive towards 400 µl, plateau at 350 µl*	*positive towards 15 min*	*positive towards 5000 xg*

Table S3: Table showing the results of our validation for each of the evaluated targeted metabolites.

Targeted metabolite	Chemical Formula	Ion Adduct	m/z-value	Retention Time (min)	R^2	Dilution range	CV IP (%)
1,3-propanediol	$C_3H_8O_2$	[M+H]+	77.05971	1.33	0.990	0.002-1	21.79
2-hydroxyhexanoic acid	$C_6H_{12}O_3$	[M-H]-	131.0695	9.4	0.999	0.002-1	11.48
2-hydroxyisocaproic acid	$C_6H_{12}O_3$	[M+H]+	131.06995	9.16	0.999	0.002-1	8.01
2-hydroxy 3-methylvalerate	$C_6H_{12}O_3$	[M-H]-	131.06950	9.03	0.999	0.002-1	9.17
2-methylbutyryl-L-carnitine	$C_{12}H_{23}NO_4$	[M+H]+	246.16998	8.55	0.987	0.002-1	8.42
2-phenylethylamine	$C_8H_{11}N$	[M+H]+	122.09643	6.59	0.096	0.02-1	14.08
2-piperidinone	C_5H_9NO	[M+H]+	100.07569	5	0.991	0.002-1	4.09
3-indoleacetic acid	$C_{10}H_9NO_2$	[M+H]+	176.07061	8.84	0.998	0.002-1	7.61
3-methoxytyramine hydroxychloride	$C_9H_{13}NO_2$	[M+H]+	168.10191	4.4	1.000	0.002-1	12.00
3-methylbutyryl/isovalaryl-L-carnitine	$C_{12}H_{23}NO_4$	[M+H]+	246.16998	8.21	0.954	0.002-1	9.38
4-guanidinobutyric acid	$C_5H_{11}N_3O_2$	[M+H]+	146.09220	1.44	0.987	0.002-1	7.60
4-hydroxyphenylpyruvic acid	$C_9H_8O_4$	[M-H]-	179.03498	4.55	0.999	0.002-1	6.02
4-methyl-2-oxovaleric acid	$C_6H_{10}O_3$	[M-H]-	129.05432	8.41	1.000	0.002-1	6.72
5-hydroxyindole-3-acetic acid	$C_{10}H_9NO_3$	[M+H]+	192.06552	8.04	0.996	0.002-1	7.27
6-acetyl-L-lysine	$C_8H_{16}N_2O_3$	[M+H]+	189.12337	1.47	0.990	0.002-1	9.20
7-methylguanine	$C_6H_7N_5O$	[M+H]+	166.07234	1.62	0.981	0.002-1	4.26
acetophenone	C_8H_8O	[M+H]+	121.06479	11.28	0.709	0.002-1	6.97
acetylcholine	$C_7H_{16}ClNO_2$	[M+H]+	146.11756	1.1	0.995	0.01-1	9.05
adenine	$C_5H_5N_5$	[M+H]+	136.06177	1.33	0.925	0.002-1	4.40
adenosine-5-monophosphate	$C_{10}H_{14}N_5O_7P$	[M+H]+	348.07036	1.47	0.985	0.002-1	28.53
adipoylcarnitine C16	$C_{13}H_{23}NO_6$	[M+H]+	290.15981	6.16	0.991	0.002-1	13.11
alfa-hydroxyisobutyric acid	$C_4H_8O_3$	[M+H]+	105.05462	2.5	0.997	0.002-1	23.37
allantoin	$C_4H_6N_4O_3$	[M+H]+	159.05127	1	0.899	0.002-0.1	20.63
anandamide (18:1)	$C_{20}H_{39}NO_2$	[M+H]+	326.30536	15.04	0.850	0.002-1	14.20
azelaic acid	$C_9H_{16}O_4$	[M-H]-	187.09758	8.83	0.994	0.002-1	11.82
B-hydroxyisovaleric acid	$C_5H_{10}O_3$	[M-H]-	117.05572	3.9	0.998	0.002-1	23.04
butyrylcarnitine	$C_{11}H_{21}NO_4$	[M+H]+	232.15433	6.58	0.999	0.002-1	7.67

chenodeoxycholic acid (CDCA)	$C_{24}H_{40}O_4$	[M-H]-	391.28534	12.9		0.998	0.002-1	7.25
cholic acid	$C_{24}H_{40}O_5$	[M-H]-	407.2803	11.99		0.978	0.002-1	15.18
choline	$C_5H_{14}NO$	[M+H]+	104.10699	0.83		0.966	0.002-1	4.72
corticosterone	$C_{21}H_{30}O_4$	[M+H]+	347.22170	11.54		0.999	0.002-1	7.86
cortisol	$C_{21}H_{30}O_5$	[M+H]+	363.21660	11.17		0.984	0.002-1	7.57
creatine	$C_4H_9N_3O_2$	[M+H]+	132.0768	0.94		0.907	0.002-1	5.51
creatinine	$C_4H_7N_3O$	[M+H]+	114.0661	0.89		0.964	0.002-1	5.35
cytidine	$C_9H_{13}N_3O_5$	[M+H]+	244.0928	1.32		0.964	0.002-1	7.69
cytosine	$C_4H_5N_3O$	[M+H]+	112.05054	1.5		0.982	0.002-1	5.81
Deoxycholic acid (DCA)	$C_{24}H_{40}O_4$	[M-H]-	391.28534	13.05		0.998	0.002-1	7.25
dipropyl disulfide	$C_6H_{14}S_2$	[M+H]+	151.06097	1.5		0.989	0.002-1	11.05
dodecanedioic acid	$C_{12}H_{22}O_4$	[M-H]-	229.14453	9.44		0.987	0.002-1	3.38
pantothenic acid	$C_9H_{17}NO_5$	[M+H]+	220.11795	6.5		0.999	0.002-1	4.10
GABA	$C_4H_9NO_2$	[M+H]+	104.07061	0.92		0.972	0.002-1	10.33
gamma-glutamylphenylalanine	$C_{14}H_{18}N_2O_5$	[M+H]+	295.12884	8.03		1.000	0.002-1	8.03
glucose	$C_6H_{12}O_6$	[M+Na]+	203.05261	0.91		0.917	0.002-1	3.41
glyceric acid	$C_3H_6O_4$	[M-H]-	105.01779	1.09		0.999	0.002-1	4.53
guanine	$C_5H_5N_5O$	[M+H]+	152.05669	1.36		0.899	0.02-1	30.80
hexadecamide	$C_{16}H_{33}NO$	[M+H]+	256.26349	15.18		0.647	0.002-0.1	11.04
hippuric acid	$C_9H_9NO_3$	[M+H]+	180.06529	8.45		0.997	0.002-1	6.27
homocysteine	$C_4H_9NO_2S$	[M+H]+	136.04268	1.13		0.966	0.002-1	25.39
hypoxanthine	$C_5H_4N_4O$	[M+H]+	137.04579	1.48		0.980	0.002-1	4.43
imidazolepropionic acid	$C_6H_8N_2O_2$	[M+H]+	141.06585	1.38		0.988	0.002-1	9.89
isobutyrylcarnitine	$C_{11}H_{21}NO_4$	[M+H]+	232.15386	6.48		1.000	0.002-1	6.71
kynurenic acid	$C_{10}H_7NO_3$	[M+H]+	190.04987	7.83		0.993	0.002-1	7.29
kynurenine	$C_{10}H_{12}N_2O_3$	[M+H]+	209.09207	4.55		0.998	0.002-1	9.91
lactic acid	$C_3H_6O_3$	[M-H]-	89.0244173	1.43		0.955	0.002-1	5.60
arginine	$C_6H_{14}N_4O_2$	[M+H]+	175.11895	0.78		0.792	0.02-1	9.01
carnitine	$C_7H_{15}NO_3$	[M+H]+	162.1125	0.87		0.982	0.002-0.1	7.16
citrulline	$C_6H_{13}N_3O_3$	[M+H]+	176.10297	0.97		0.906	0.01-1	24.04

dehydroascorbic acid	$C_6H_6O_6$	[M-H]-	173.00916	1.16	0.864	0.02-1	7.00
glutamine	$C_5H_{10}N_2O_3$	[M+H]+	147.07642	0.86	0.630	0.002-0.2	5.93
linoleamide	$C_{18}H_{33}NO$	[M+H]+	280.26349	14.71	0.823	0.002-0.1	8.45
isoleucine	$C_6H_{13}NO_2$	[M+H]+	132.10191	2.2	0.999	0.002-1	9.90
leucine	$C_6H_{13}NO_2$	[M+H]+	132.10191	2.4	0.999	0.002-1	8.75
lysine	$C_6H_{14}N_2O_2$	[M+H]+	147.1128	0.75	0.883	0.002-0.2	10.19
methionine	$C_5H_{11}NO_2S$	[M+H]+	150.05833	1.42	0.986	0.002-1	10.65
phenylalanine	$C_9H_{11}NO_2$	[M+H]+	166.08626	5.09	1.000	0.002-1	5.88
pyroglutamic acid	$C_5H_7NO_3$	[M+H]+	130.04987	1.6	0.993	0.002-1	3.39
threonine	$C_4H_9NO_3$	[M+H]+	120.06552	0.87	0.981	0.002-0.2	11.06
tyrosine	$C_9H_{11}NO_3$	[M+H]+	182.08117	1.83	0.999	0.002-1	4.10
valine 5-aminovaleric acid	$C_5H_{11}NO_2$	[M+H]+	118.08626	1.13	0.986	0.002-1	8.06
lyso-phosphatidylcholine (18:0)	$C_{26}H_{52}NO_7P$	[M+H]+	522.35542	13.67	0.985	0.002-1	18.45
malic acid	$C_4H_6O_5$	[M-H]-	133.01424	1.19	0.981	0.002-0.2	8.42
malonylcarnitine	$C_{10}H_{17}NO_6$	[M+H]+	248.11286	1.13	0.893	0.02-1	5.93
mannose	$C_6H_{12}O_6$	[M-H]-	179.05611	1	0.916	0.002-1	2.86
N,N-dimethylarginine	$C_8H_{18}N_4O_2$	[M+H]+	203.15025	0.93	0.997	0.002-0.1	8.64
N6-methyladenosine	$C_{11}H_{15}N_5O_4$	[M+H]+	282.11968	4.2	0.999	0.002-1	6.70
N-acetylarginine	$C_8H_{16}N_4O_3$	[M+H]+	217.12952	1.16	0.981	0.002-1	14.71
N-acetylasparagine	$C_6H_{10}N_2O_4$	[M+H]+	175.07133	1.16	0.980	0.002-0.1	15.31
N-acetyl-beta-alanine	$C_5H_9NO_3$	[M+H]+	132.06552	1.68	0.946	0.002-1	13.34
N-acetylglutamic acid	$C_7H_{11}NO_5$	[M+H]+	190.071	1.76	0.995	0.002-1	9.44
N-acetyl-L-methionine	$C_7H_{13}NO_3S$	[M+H]+	192.06889	7.6	0.995	0.002-1	15.42
N-acetyl-L-phenylalanine	$C_{11}H_{13}NO_3$	[M+H]+	208.09682	10.17	0.994	0.002-1	7.33
N-acetyltryptophan	$C_{13}H_{14}N_2O_3$	[M+H]+	247.1077	10.42	0.994	0.002-1	11.85
N-acetyltyrosine	$C_{11}H_{13}NO_4$	[M+H]+	224.09173	7.67	0.993	0.002-1	13.47
N-acetylvaline/N-isovalerylglycine	$C_7H_{13}NO_3$	[M+H]+	160.09682	7.6	0.997	0.002-1	12.85
nicotinamide	$C_6H_6N_2O$	[M+H]+	123.05530	1.59	0.977	0.002-1	6.01
O-Acetyl-L carnitine	$C_9H_{17}NO_4$	[M+H]+	204.12303	1.5	0.998	0.002-1	12.28
oleamide	$C_{18}H_{35}NO$	[M+H]+	282.27914	15.34	0.673	0.002-0.1	10.15

O-succinylcarnitine	$C_{11}H_{19}NO_6$	[M+H]+	262.12851	1.75		0.999	0.002-1	3.11
oxoglutaric acid	$C_5H_6O_5$	[M+H]+	147.02880	1.4		0.957	0.002-1	8.89
pantothenol	$C_9H_{19}NO_4$	[M+H]+	206.13868	6.63		0.927	0.002-0.5	5.97
phenylacetaldehyde	C_8H_8O	[M+H]+	121.06479	11		0.689	0.02-1	7.25
phenylAc-Gln-OH	$C_{13}H_{16}N_2O_4$	[M+H]+	265.11828	8.54		0.996	0.002-1	8.03
propionyl carnitine C3	$C_{10}H_{19}NO_4$	[M+H]+	218.13868	3.01		0.998	0.002-1	8.22
pyruvic acid	$C_3H_4O_3$	[M-H]-	87.00877	1.16		0.905	0.002-1	9.98
saccharic acid	$C_6H_{10}O_8$	[M-H]-	209.03029	1.04		0.987	0.002-0.1	10.16
sebacic acid	$C_{10}H_{18}O_4$	[M-H]-	201.11323	9.03		0.996	0.002-1	6.69
sodium taurocholate (TCA)	$C_{26}H_{44}NNaO_7S$	[M-H]-	514.28440	11.26		0.994	0.002-1	8.58
spermidine	$C_7H_{19}N_3$	[M+H]+	146.16517	0.75		0.950	0.002-1	18.84
succinic acid	$C_4H_6O_4$	[M-H]-	117.01933	1.96		0.999	0.002-1	5.90
threonic acid	$C_4H_8O_5$	[M+H]+	137.04445	0.95		1.000	0.002-0.2	5.28
tryptophan	$C_{11}H_{12}N_2O_2$	[M+H]+	205.09715	7.5		1.000	0.002-1	8.33
valerylcarnitine	$C_{12}H_{23}NO_4$	[M+H]+	246.16940	8.2		0.991	0.002-1	13.74

IP: Instrumental precision; The R^2 is shown as obtained for the mentioned dilution range.

Table S4: Results for the univariate analysis performed for the targeted metabolites included in the adult dog cohort for comparison of IE with non-brain related diseases. Mean, median and standard deviation (SD) for the iQC normalized peak areas is mentioned (ratio: peak area metabolite/peak area iQC).

**Metabolite**	**Hypothesis test**	**Group Nrs**	**Amount per group**	**Mean per group**	**Median per group**	**SD per group**	***P*-value**	**Fold change**	**Log2FC**
2-hydroxy 3-methylvaleric acid	Wilcoxon rank sum exact test	-1; 1	7; 8	0.898; 1.351	1.078; 1.435	0.385; 0.502	0.151981352	0.664692823	-0.589240319
2-hydroxyhexanoic_acid	Wilcoxon rank sum exact test	-1; 1	7; 8	0.850; 1.484	1.011; 1.597	0.358; 0.622	0.093861694	0.572776283	-0.803956338
2-hydroxyisocaproic_acid	Welch Two Sample t-test	-1; 1	7; 8	0.880; 1.173	1.063; 1.328	0.367; 0.327	0.130002619	0.750213131	-0.41462758
2-methylbutyryl-L-carnitine	Wilcoxon rank sum exact test	-1; 1	7; 8	2.025; 0.557	1.356; 0.685	2.161; 0.272	0.093861694	**3.635547529**	**1.862172657**
**2-phenylethylamine**	Welch Two Sample t-test	-1; 1	7; 8	0.865; 0.972	0.864; 0.960	0.029; 0.102	**0.021074691**	0.889917697	-0.168256179
2-piperidinone	Wilcoxon rank sum exact test	-1; 1	7; 8	0.781; 1.075	0.858; 0.883	0.381; 0.889	0.866511267	0.72651163	-0.460942201
3-indoleacetic_acid	Welch Two Sample t-test	-1; 1	7; 8	1.089; 1.175	0.873; 1.129	0.684; 0.526	0.792555639	0.926808511	-0.109656802
3-methylbutyryl_Isovalarayl-L-carnitine	Wilcoxon rank sum exact test	-1; 1	7; 8	2.119; 0.529	1.501; 0.581	2.240; 0.262	0.072105672	**4.005671021**	**2.00204394**
4-guanidinobutyric_acid	Welch Two Sample t-test	-1; 1	7; 8	1.039; 0.854	0.974; 0.771	0.586; 0.317	0.475523758	1.216627632	0.282887676
4-hydroxyphenylpyruvic_acid	Wilcoxon rank sum exact test	-1; 1	7; 8	0.895; 1.298	0.522; 1.074	1.064; 0.740	0.120590521	0.689522344	-0.536330791
4-methyl_2-oxovaleric_acid	Welch Two Sample t-test	-1; 1	7; 8	0.789; 1.288	0.688; 1.268	0.380; 0.511	0.050306309	0.612577643	-0.707035381
5-hydroxyindole_acetic_acid	Wilcoxon rank sum exact test	-1; 1	7; 8	0.840; 1.249	0.668; 0.963	0.558; 0.695	0.151981352	0.672538033	-0.572312238
7-methylguanine	Welch Two Sample t-test	-1; 1	7; 8	0.915; 1.285	0.818; 1.301	0.384; 0.270	0.05698107	0.712062259	-0.489924706
acetophenone	Wilcoxon rank sum exact test	-1; 1	7; 8	2.765; 1.031	1.082; 1.027	4.455; 0.033	**0.009324009**	**2.681862253**	**1.423235139**

acetylcholine	Wilcoxon rank sum exact test	-1; 1	7; 8	1.188; 0.774	1.050; 0.752	0.572; 0.189	0.054079254	1.534883714	0.618129358
adenine	Wilcoxon rank sum exact test	-1; 1	7; 8	2.098; 0.169	0.194; 0.162	3.975; 0.073	0.280963481	**12.41420051**	**3.633919448**
adenosine-5_monophosphate	Wilcoxon rank sum exact test	-1; 1	7; 8	2.068; 1.238	2.007; 0.267	2.357; 2.860	0.463403263	1.670436182	0.740224866
adipoylcarnitine_C16	Welch Two Sample t-test	-1; 1	7; 8	0.658; 0.345	0.589; 0.344	0.574; 0.178	0.207505787	1.907246351	0.931491202
alfa-hydroxyisobutyric_acid	Welch Two Sample t-test	-1; 1	7; 8	1.095; 1.070	1.203; 1.093	0.490; 0.325	0.910812613	1.023364486	0.033320073
allantoin	Welch Two Sample t-test	-1; 1	7; 8	1.496; 0.534	1.119; 0.510	1.126; 0.220	0.065110555	**2.801498094**	**1.486198511**
anandamide_C18_1	Wilcoxon rank sum exact test	-1; 1	7; 8	0.303; 2.058	0.303; 0.516	0.158; 3.140	0.072105672	0.147230325	-2.763853243
azelaic_acid	Welch Two Sample t-test	-1; 1	7; 8	1.317; 1.449	1.332; 1.353	0.256; 0.270	0.349485268	0.908902692	-0.137802248
B-hydroxyisovaleric_acid	Welch Two Sample t-test	-1; 1	7; 8	0.911; 1.135	0.968; 1.133	0.329; 0.275	0.181699267	0.802643174	-0.317169335
choline	Wilcoxon rank sum exact test	-1; 1	7; 8	1.008; 0.502	0.507; 0.503	0.929; 0.088	0.866511267	**2.007968107**	**1.005736355**
**cortisol**	Welch Two Sample t-test	-1; 1	7; 8	0.228; 0.989	0.111; 0.864	0.219; 0.642	**0.012121801**	0.230535903	-2.116936648
creatine	Welch Two Sample t-test	-1; 1	7; 8	1.310; 1.123	1.001; 1.070	0.890; 0.331	0.615508419	1.166518253	0.222208882
**creatinine**	Welch Two Sample t-test	-1; 1	7; 8	0.927; 1.426	0.890; 1.452	0.167; 0.303	**0.001992792**	0.650070129	-0.621332732
cytidine	Wilcoxon rank sum exact test	-1; 1	7; 8	2.608; 0.421	0.697; 0.381	3.394; 0.134	0.335664336	**6.194774223**	**2.631051702**
cytosine	Wilcoxon rank sum exact test	-1; 1	7; 8	2.020; 1.070	1.229; 0.876	1.638; 0.420	0.189277389	1.887850459	0.91674449
**glucose**	Welch Two Sample t-test	-1; 1	7; 8	0.809; 1.243	0.958; 1.195	0.330; 0.168	**0.012375596**	0.650844733	-0.619614682
dipropyl_disulfide	Welch Two Sample t-test	-1; 1	7; 8	1.135; 1.130	1.145; 1.083	0.457; 0.406	0.980637629	1.004424779	0.006369525

dodecanedioic_acid	Welch Two Sample t-test	-1; 1	7; 8	0.974; 1.080	0.917; 1.094	0.150; 0.188	0.248746913	0.901851853	-0.149037634
pantothenic_acid	Welch Two Sample t-test	-1; 1	7; 8	0.943; 1.327	0.733; 1.237	0.850; 0.781	0.381307011	0.710625473	-0.49283869
GABA_2-aminoisobutyric_acid	Welch Two Sample t-test	-1; 1	7; 8	0.928; 1.035	0.527; 1.036	0.686; 0.511	0.740450925	0.896618358	-0.157434056
gamma-glutamylphenylalanine	Wilcoxon rank sum exact test	-1; 1	7; 8	1.209; 1.130	1.153; 1.081	0.713; 0.505	0.866511267	1.069911504	0.097491471
glyceric_acid	Welch Two Sample t-test	-1; 1	7; 8	0.972; 1.104	0.996; 1.148	0.260; 0.403	0.457234393	0.880434784	-0.183711951
hexadecamide	Welch Two Sample t-test	-1; 1	7; 8	1.093; 1.137	0.706; 1.026	0.895; 0.736	0.918534234	0.961301671	-0.056938853
**hippuric_acid**	Wilcoxon rank sum exact test	-1; 1	7; 8	0.599; 1.933	0.173; 1.477	1.009; 1.556	**0.013986014**	0.309881018	-1.690213713
homocysteine	Welch Two Sample t-test	-1; 1	7; 8	1.197; 1.318	1.479; 1.264	0.492; 0.542	0.657965237	0.908194234	-0.138927217
hypoxanthine	Wilcoxon rank sum exact test	-1; 1	7; 8	2.492; 0.110	0.977; 0.090	3.913; 0.064	0.335664336	**22.65454349**	**4.501728514**
imidazolepropionic_acid	Wilcoxon rank sum exact test	-1; 1	7; 8	0.794; 1.098	0.629; 0.979	0.781; 0.627	0.231857032	0.723132972	-0.467667137
isobutyrylcarnitine	Wilcoxon rank sum exact test	-1; 1	7; 8	2.267; 0.441	0.547; 0.446	3.204; 0.117	0.280963481	**5.140589475**	**2.361933804**
kynurenic_acid	Wilcoxon rank sum exact test	-1; 1	7; 8	1.162; 0.703	0.427; 0.594	1.624; 0.457	0.866511267	1.652916065	0.725013466
kynurenine	Welch Two Sample t-test	-1; 1	7; 8	1.220; 1.226	0.995; 1.338	0.897; 0.436	0.987000087	0.995106036	-0.007077831
lactic_acid	Welch Two Sample t-test	-1; 1	7; 8	1.310; 0.726	1.195; 0.801	0.721; 0.156	0.076929028	1.804407702	0.85152535
arginine	Welch Two Sample t-test	-1; 1	7; 8	1.002; 1.171	0.924; 1.109	0.345; 0.545	0.482617374	0.855678908	-0.224858565
carnitine	Wilcoxon rank sum exact test	-1; 1	7; 8	1.117; 1.328	0.774; 1.147	0.810; 0.485	0.231857032	0.841114459	-0.249625959
citrulline	Welch Two Sample t-test	-1; 1	7; 8	0.828; 0.989	0.934; 0.887	0.772; 0.441	0.638512182	0.837209304	-0.25633975

glutamine	Welch Two Sample t-test	-1; 1	7; 8	1.070; 1.109	1.094; 1.068	0.223; 0.220	0.735326258	0.964833183	-0.051648568
linoleamide	Welch Two Sample t-test	-1; 1	7; 8	0.926; 1.122	0.611; 1.086	0.745; 0.626	0.595654255	0.825311945	-0.276988575
isoleucine	Welch Two Sample t-test	-1; 1	7; 8	1.118; 1.158	1.029; 1.367	0.396; 0.449	0.857146927	0.965457686	-0.050715065
leucine	Welch Two Sample t-test	-1; 1	7; 8	1.049; 1.215	1.167; 1.313	0.292; 0.417	0.385563816	0.863374487	-0.211941634
lysine	Welch Two Sample t-test	-1; 1	7; 8	1.027; 1.164	1.034; 1.114	0.251; 0.347	0.392159148	0.882302407	-0.180654875
methionine	Welch Two Sample t-test	-1; 1	7; 8	1.106; 1.187	1.110; 1.179	0.428; 0.420	0.721097242	0.931760742	-0.101968549
phenylalanine	Welch Two Sample t-test	-1; 1	7; 8	1.141; 1.175	1.102; 1.192	0.353; 0.303	0.847507548	0.97106383	-0.042361965
pyroglutamic_acid	Welch Two Sample t-test	-1; 1	7; 8	0.754; 0.917	0.742; 0.856	0.185; 0.349	0.274201362	0.822246458	-0.282357207
threonine	Welch Two Sample t-test	-1; 1	7; 8	0.932; 1.408	0.811; 1.340	0.393; 0.555	0.075875036	0.661931821	-0.595245469
tyrosine	Welch Two Sample t-test	-1; 1	7; 8	1.064; 1.235	1.059; 1.136	0.332; 0.426	0.398336505	0.861538463	-0.215012889
malic_acid	Welch Two Sample t-test	-1; 1	7; 8	1.436; 0.689	0.991; 0.682	1.401; 0.418	0.217207082	**2.084179955**	**1.05947985**
malonylcarnitine	Welch Two Sample t-test	-1; 1	7; 8	1.469; 1.335	1.632; 1.303	0.777; 0.772	0.74413867	1.100374531	0.137994653
**mannose**	Welch Two Sample t-test	-1; 1	7; 8	0.810; 1.237	0.967; 1.193	0.328; 0.167	**0.013054937**	0.654810027	-0.610851681
methoxytyramine	Wilcoxon rank sum exact test	-1; 1	7; 8	1.890; 0.724	1.553; 0.704	1.593; 0.302	0.093861694	**2.610497215**	**1.38432462**
N_N-dimethylarginine	Wilcoxon rank sum exact test	-1; 1	7; 8	1.138; 1.838	1.174; 1.380	0.299; 1.089	0.280963481	0.619151253	-0.691636204
N6-acetyl-L-lysine	Welch Two Sample t-test	-1; 1	7; 8	0.943; 1.723	0.979; 1.639	0.283; 1.009	0.068768384	0.547301221	-0.869593019
N6-methyladenosine	Welch Two Sample t-test	-1; 1	7; 8	0.628; 0.795	0.583; 0.693	0.277; 0.290	0.275240414	0.78993711	-0.340190296

N-acetyl_arginine	Welch Two Sample t-test	-1; 1	7; 8	0.975; 1.301	0.869; 1.354	0.297; 0.370	0.081022013	0.749423522	-0.416146834
N-acetyl_asparagine	Welch Two Sample t-test	-1; 1	7; 8	1.128; 0.788	1.114; 0.660	0.475; 0.455	0.182876989	1.431472076	0.517499527
N-acetyl-beta-alanine	Wilcoxon rank sum exact test	-1; 1	7; 8	0.989; 1.000	0.898; 0.819	0.485; 0.443	0.955089355	0.989	-0.015957574
N-acetylglutamic_acid	Welch Two Sample t-test	-1; 1	7; 8	1.174; 1.149	0.963; 1.130	0.886; 0.418	0.94570414	1.02175805	0.03105361
N-acetyl-L-methionine	Wilcoxon rank sum exact test	-1; 1	7; 8	1.420; 0.996	1.059; 0.871	1.392; 0.444	0.612587413	1.425702807	0.511673278
N-acetyl-L-phenylalanine	Welch Two Sample t-test	-1; 1	7; 8	1.047; 1.184	1.199; 1.121	0.454; 0.381	0.541231224	0.884290542	-0.177407637
N-acetyltryptophan	Welch Two Sample t-test	-1; 1	7; 8	0.938; 1.071	0.943; 0.985	0.328; 0.389	0.486328863	0.875816995	-0.19129865
N-acetyltyrosine	Welch Two Sample t-test	-1; 1	7; 8	0.828; 1.190	0.968; 1.134	0.360; 0.431	0.099567327	0.695798322	-0.523258895
N-acetylvaline	Welch Two Sample t-test	-1; 1	7; 8	1.034; 1.132	1.110; 1.027	0.279; 0.321	0.537189093	0.913427563	-0.130637771
nicotinamide	Wilcoxon rank sum exact test	-1; 1	7; 8	1.110; 1.291	0.975; 1.050	0.779; 0.709	0.535819736	0.859798607	-0.217929322
O-acetyl L-carnitine	Welch Two Sample t-test	-1; 1	7; 8	1.318; 0.611	0.737; 0.561	1.083; 0.269	0.138615309	**2.157119457**	**1.109106073**
oleamide	Welch Two Sample t-test	-1; 1	7; 8	0.808; 0.986	0.691; 1.015	0.438; 0.289	0.380449461	0.819472618	-0.28723235
O-succinylcarnitine	Welch Two Sample t-test	-1; 1	7; 8	1.122; 1.031	1.219; 1.040	0.491; 0.323	0.684475796	1.088263821	0.122028342
oxoglutaric_acid	Welch Two Sample t-test	-1; 1	7; 8	1.017; 1.115	0.965; 1.179	0.395; 0.360	0.626353172	0.912107624	-0.13272403
**pantothenol**	Welch Two Sample t-test	-1; 1	7; 8	0.595; 1.820	0.646; 1.666	0.141; 1.164	**0.020570625**	0.326923081	-1.612976861
phenylacetalaldehyde	Welch Two Sample t-test	-1; 1	7; 8	1.041; 1.038	1.048; 1.056	0.056; 0.051	0.909017879	1.002890173	0.004163625
phenylAc-GLN-OH	Wilcoxon rank sum exact test	-1; 1	7; 8	0.903; 0.991	0.639; 0.776	0.690; 0.752	0.612587413	0.911200808	-0.134159068

propionyl_carnitine_C3	Welch Two Sample t-test	-1; 1	7; 8	1.755; 0.656	1.510; 0.503	1.696; 0.364	0.140253299	**2.675304853**	**1.419703297**
pyruvic_acid	Welch Two Sample t-test	-1; 1	7; 8	1.086; 1.028	0.835; 0.900	0.832; 0.761	0.890835996	1.056420233	0.079183838
saccharic_acid	Wilcoxon rank sum exact test	-1; 1	7; 8	0.845; 0.971	0.728; 0.986	0.240; 0.275	0.463403263	0.870236871	-0.200519952
sebacic_acid	Wilcoxon rank sum exact test	-1; 1	7; 8	0.850; 1.054	0.835; 0.941	0.094; 0.294	0.054079254	0.806451615	-0.310340117
**spermidine**	Wilcoxon rank sum exact test	-1; 1	7; 8	2.371; 0.377	1.013; 0.173	3.099; 0.622	**0.020512821**	**6.289124528**	**2.652859202**
succinic_acid	Wilcoxon rank sum exact test	-1; 1	7; 8	2.053; 0.518	1.088; 0.509	2.530; 0.099	0.463403263	**3.963320406**	**1.986709604**
TCA	Wilcoxon rank sum exact test	-1; 1	7; 8	1.625; 0.329	0.944; 0.310	2.422; 0.160	0.335664336	**4.939209607**	**2.304280194**
threonic_acid	Welch Two Sample t-test	-1; 1	7; 8	1.275; 1.077	1.152; 1.033	0.487; 0.340	0.388410583	1.183844009	0.243478995
tryptophan	Welch Two Sample t-test	-1; 1	7; 8	1.115; 1.050	1.029; 1.117	0.388; 0.260	0.715023566	1.061904761	0.086654381
valerylcarnitine_C5	Wilcoxon rank sum exact test	-1; 1	7; 8	2.241; 0.548	1.502; 0.582	2.416; 0.291	0.072105672	**4.089416002**	**2.031894831**
valine_aminovaleric_acid	Welch Two Sample t-test	-1; 1	7; 8	1.135; 1.073	1.117; 1.116	0.427; 0.430	0.78500911	1.057781919	0.081042221

Table S5: Targeted metabolites detected for the first time in CSF, as evaluated in the validation.

Metabolite	Chemical Formula	Ion adduct	m/z-value	RT	Class	Detected before in
saccharic acid	$C_6H_{10}O_8$	[M-H]-	209.03029	1.04	organooxygen compounds	blood, faeces, urine
dipropyl disulfide	$C_6H_{14}S_2$	[M+H]+	151.06097	1.5	organic disulfides	faeces
N-acetyl-L-glutamic acid	$C_7H_{11}NO_5$	[M+H]+	190.071	1.76	carboxylic acids and derivatives	blood, faeces, urine, saliva
N-acetyl-L-methionine	$C_7H_{13}NO_3S$	[M+H]+	192.06889	7.6	carboxylic acids and derivatives	blood, faeces, urine, saliva
cytosine	$C_4H_5N_3O$	[M+H]+	112.05054	1.5	diazines	blood, faeces, urine
sodium taurocholate (TCA)	$C_{26}H_{44}NNaO_7S$	[M-H]-	514.2844	11.26	steroids and derivatives	blood, faeces, urine, bile
7-methylguanine	$C_6H_7N_5O$	[M+H]+	166.07234	1.62	imidazopyrimidines	blood, faeces, urine
gamma-glutamylphenylalanine	$C_{14}H_{18}N_2O_5$	[M+H]+	295.12884	8.03	carboxylic acids and derivatives	blood, faeces, urine

Table S6: Analytical standards and internal standards used in the study.

Analytical standard	Chemical Formula	Ion Adduct	m/z-value	Retention Time (min)	Optimization	Validation
alanine-d3	$CD_3CH(NH_2)COOH$	[M+H]+	93.07378	0.97	ISTD	ISTD
dopamine.HCl-d4	$C_8H_7D_4NO_2.HCl$	[M+H]+	158.11113	1.47	ISTD	ISTD
1,3-propanediol	$C_3H_8O_2$	[M+H]+	77.05971	1.33	x	x
1,3-dimethoxybenzene	$C_8H_{10}O_2$	[M+H]+	139.07536	11.94		
13-OxoODE	$C_{18}H_30O_3$	[M+H]+	295.22599	13.58		
1-acetyl-3-indolecarboxaldehyde	$C_{11}H_9NO_2$	[M+H]+	188.07061	10.99		
1-methylguanidine	$C_2H_8ClN_3$	[M+H]+	74.01712	0.97		
1-methylhistamine	$C_6H_{11}N_3$	[M+H]+	126.10257	0.85		
1-methyl-L-histidine	$C_7H_{11}N_3O_2$	[M+H]+	170.0924	0.79	x	
1-octen-3-one	$C_8H_{14}O$	[M+H]+	127.11174	12.46		
2-(dimethyloamino)acetonitrile	$C_4H_8N_2$	[M+H]+	85.07602	1		
2,3-heptanedione	$C_7H_{12}O_2$	[M+H]+	129.09101	8.4		
2,3-pentanedione	$C_5H_8O_2$	[M+H]+	101.05971	1.17		
2.6-diaminopimelic acid	$C_7H_{14}N_2O_4$	[M+H]+	191.10263	0.85	x	
2-acetyl-5-methylfuran	$C_7H_8O_2$	[M+H]+	125.05971	10.47		
2-deoxyuridine	$C_9H_{12}N_2O_5$	[M+H]+	229.08190	2.74		
2-dodecanone	$C_{12}H_{24}O$	[M+H]+	185.18999	15		
2-hexanone	$C_6H_{12}O$	[M+H]+	101.09609	11.37		
3-hydroxybutyric acid	$C_4H_8O_3$	[M+H]+	105.05462	2.4	x	
2-hydroxyhexanoic acid	$C_6H_{12}O_3$	[M-H]-	131.0695	9.4	x	x
2-hydroxyisocaproic acid	$C_6H_{12}O_3$	[M+H]+	131.06995	9.16	x	x
2-hydroxy 3-methylvalerate	$C_6H_{12}O_3$	[M-H]-	131.06950	9.03		x
2-methylbutyryl-L-carnitine	$C_{12}H_{23}NO_4$	[M+H]+	246.16998	8.55		x
2-pentanone	$C_5H_{10}O$	[M+H]+	87.08095	9.97		
2-phenylethylamine	$C_8H_{11}N$	[M+H]+	122.09643	6.59		x
2-piperidinone	C_5H_9NO	[M+H]+	100.07569	5	x	x
3,4-dihydroxybenzoic acid	$C_7H_6O_4$	[M-H]-	153.01933	4.7		

Analytical standard	Chemical Formula	Ion Adduct	m/z-value	Retention Time (min)	Optimization	Validation
3,4-dihydroxyphenylalanine (L-DOPA)	$C_9H_{11}NO_4$	[M+H]+	198.07608	1.55		
3,4-Dihydroxyphenylpropionic acid	$C_9H_{10}O_4$	[M+H]+	183.06519	8.89	x	
3,5-dihydroxybenzoic acid	$C_7H_6O_4$	[M+H]+	155.03389	6.12		
3-amino-2-piperidone	$C_5H_{10}N_2O$	[M+H]+	115.08658	0.92		
3-ethylphenol	$C_8H_{10}O$	[M+H]+	123.08044	11.56		
3-heptanone	$C_7H_{14}O$	[M+H]+	115.11174	12.11		
3-hydroxy DL-kynurenin	$C_{10}H_{12}N_2O_4$	[M+H]+	225.08698	1.9		
3-hydroxy-3-methylglutarate	$C_6H_{10}O_5$	[M+H]+	163.06010	2.59		
3-Hydroxy-4-methoxycinnamic acid	$C_{10}H_{10}O_4$	[M+H]+	195.06519	10.44		
3-hydroxypyridine	C_8H_8O	[M+H]+	96.04439	1.08		
3-indoleacetic acid	$C_{10}H_9NO_2$	[M+H]+	176.07061	8.84	x	x
3-methoxytyramine hydroxychloride	$C_9H_{13}NO_2$	[M+H]+	168.10191	4.4		x
3-methyl-2-cyclohexen-1-one	$C_7H_{10}O$	[M+H]+	111.08044	10.14		
3-methyl-2-oxobutyric acid	$C_5H_8NaO_3$	[M+H]+	117.05462	2.67		
3-methyl-2-oxopentanoic acid (ketoisoleucine, pantolactone)	$C_6H_{10}O_3$	[M+H]+	131.07027	3.56		
3-methyladenine	$C_6H_7N_5$	[M+H]+	150.07742	1.41		
3-methylbutyryl/isovalaryl-L-carnitine	$C_{12}H_{23}NO_4$	[M+H]+	246.16998	8.21	x	x
3-phenyl-1-propanol	$C_9H_{12}O$	[M+H]+	137.09609	11.24	x	
3-phenylbutyric acid	$C_{10}H_{12}O_2$	[M-H]-	163.07572	11.47		
3-phenylpropionic acid	$C_9H_{10}O_2$	[M-H]-	149.0608	9.2	x	
4-guanidinobutyric acid	$C_5H_{11}N_3O_2$	[M+H]+	146.09220	1.44	x	x
4-hexen-3-one	$C_6H_{10}O$	[M+H]+	99.08044	9.78		
4-hydroxybenzaldehyde	$C_7H_6O_2$	[M+H]+	123.04406	8.87		
4-hydroxyphenylacetic acid	$C_8H_8O_3$	[M+H]+	153.05462	9.4		

Analytical standard	Chemical Formula	Ion Adduct	m/z-value	Retention Time (min)	Optimization	Validation
4-hydroxyphenylpyruvic acid	$C_{9}H_{8}O_{4}$	[M-H]-	179.03498	4.55		x
4-methyl-2-oxovaleric acid	$C_{6}H_{10}O_{3}$	[M-H]-	129.05432	8.41	x	x
4-methylcatechol	$C_{7}H_{8}O_{2}$	[M-H]-	123.04515	8.1		
4-methylvaleric acid	$C_{6}H_{12}O_{2}$	[M+H]+	117.09071	9.26		
5-hydroxyindole-3-acetic acid	$C_{10}H_{9}NO_{3}$	[M+H]+	192.06552	8.04	x	x
5-hydroxytryptophol	$C_{10}H_{11}NO_{2}$	[M+H]+	178.08626	7.86		
6-acetyl-L-lysine	$C_{8}H_{16}N_{2}O_{3}$	[M+H]+	189.12337	1.47	x	x
6-deoxyhexose	$C_{6}H_{12}O_{5}$	[M+H]+	163.06060	1.12	x	
7-ketodeoxycholate	$C_{24}H_{38}O_{5}$	[M-H]-	405.26465	11.53		
7-methylguanine	$C_{6}H_{7}N_{5}O$	[M+H]+	166.07234	1.62	x	x
abscisic acid	$C_{15}H_{2}0O_{4}$	[M+H]+	265.14344	11.05		
acetoin	$C_{4}H_{8}O_{2}$	[M+H]+	89.05971	2.41		
acetophenone	$C_{8}H_{8}O$	[M+H]+	121.06479	11.28	x	x
acetylcholine	$C_{7}H_{16}ClNO_{2}$	[M+H]+	146.11756	1.1	x	x
adenine	$C_{5}H_{5}N_{5}$	[M+H]+	136.06177	1.33	x	x
adenosine-5-monophosphate	$C_{10}H_{14}N_{5}O_{7}P$	[M+H]+	348.07036	1.47	x	x
adipic acid	$C_{6}H_{10}O_{4}$	[M+H]+	147.06519	5.1		
adipoylcarnitine C16	$C_{13}H_{23}NO_{6}$	[M+H]+	290.15981	6.16	x	x
alanine	$C_{3}H_{7}NO_{2}$	[M+H]+	90.05496	0.85		
aldosterone	$C_{21}H_{28}O_{5}$	[M+H]+	361.20095	10.95		
alfa-hydroxyisobutyric acid	$C_{4}H_{8}O_{3}$	[M+H]+	105.05462	2.5		x
allantoin	$C_{4}H_{6}N_{4}O_{3}$	[M+H]+	159.05127	1	x	x
allylanthranilate	$C_{10}H_{11}NO_{2}$	[M+H]+	178.08626	12.27		
alpha-lactose	$C_{12}H_{22}O_{11}$	[M-H]-	341.10893	0.95		
alpha-linolenic acid	$C_{18}H_{3}0O_{2}$	[M+H]+	279.23186	15		
anandamide (17:1)	$C_{19}H_{37}NO_{2}$	[M+H]+	312.28971	14.54		
anandamide (18:1)	$C_{2}0H_{39}NO_{2}$	[M+H]+	326.30536	15.04		x
arabinose/ribose	$C_{5}H_{10}O_{5}$	[M-H]-	149.04555	1.47		
arabitol/xylitol	$C_{5}H_{12}O_{5}$	[M+H]+	153.07575	1		

Analytical standard	Chemical Formula	Ion Adduct	m/z-value	Retention Time (min)	Optimization	Validation
azelaic acid	$C_{9}H_{16}O_{4}$	[M-H]-	187.09758	8.83	x	x
benzoic acid	$C_{7}H_{6}O_{2}$	[M-H]-	121.0295	8.8		
betaine	$C_{5}H_{11}NO_{2}$	[M+H]+	118.08626	0.91		
B-hydroxyisovaleric acid	$C_{5}H_{10}O_{3}$	[M-H]-	117.05572	3.9	x	x
Butyric acid	$C_{4}H_{8}O_{2}$	[M+H]+	89.05971	6.4		
butyrylcarnitine	$C_{11}H_{21}NO_{4}$	[M+H]+	232.15433	6.58	x	x
chenodeoxycholic acid (CDCA)	$C_{24}H_{4}0O_{4}$	[M-H]-	391.28534	12.9	x	x
cholic acid	$C_{24}H_{4}0O_{5}$	[M-H]-	407.2803	11.99	x	x
choline	$C_{5}H_{14}NO$	[M+H]+	104.10699	0.83		x
cinnamaldehyde	$C_{9}H_{8}O$	[M+H]+	133.06479	11.58		
Citric acid	$C_{6}H_{8}O_{7}$	[M+H]+	193.03428	1.4		
corticosterone	$C_{21}H_{3}0O_{4}$	[M+H]+	347.22170	11.54		x
cortisol	$C_{21}H_{3}0O_{5}$	[M+H]+	363.21660	11.17	x	x
cortisone	$C_{21}H_{28}O_{5}$	[M+H]+	361.20095	11.18		
coumaric acid	$C_{9}H_{8}O_{3}$	[M+H]+	165.05462	10.28		
creatine	$C_{4}H_{9}N_{3}O_{2}$	[M+H]+	132.0768	0.94	x	x
creatinine	$C_{4}H_{7}N_{3}O$	[M+H]+	114.0661	0.89	x	x
cyclohexylamine	$C_{6}H_{13}N$	[M+H]+	100.11208	3.5	x	
cytidine	$C_{9}H_{13}N_{3}O_{5}$	[M+H]+	244.0928	1.32		x
cytosine	$C_{4}H_{5}N_{3}O$	[M+H]+	112.05054	1.5	x	x
decanoyl-L-carnitine (10:0)	$C_{17}H_{33}NO_{4}$	[M+H]+	316.24824	11.28		
Deoxycholic acid (DCA)	$C_{24}H_{4}0O_{4}$	[M-H]-	391.28534	13.05		x
deoxycorticosterone	$C_{21}H_{3}0O_{3}$	[M+H]+	331.26677	12.15		
D-erythronic acid	$C_{4}H_{8}O_{5}$	[M+H]+	137.04445	1		
dihydroorotic acid	$C_{5}H_{6}N_{2}O_{4}$	[M+H]+	159.04003	1.05		
dipropyl disulfide	$C_{6}H_{14}S_{2}$	[M+H]+	151.06097	1.5	x	x
DL-2-aminoadipic acid	$C_{6}H_{11}NO_{4}$	[M+H]+	162.07608	0.97		
DL-hexanoylcarnitine (C6)	$C_{13}H_{25}NO_{4}$	[M+H]+	260.18563	10.14		
DL-leucyl DL-phenylalanine	$C_{15}H_{22}N_{2}O_{3}$	[M+H]+	279.17031	7.69		

Analytical standard	Chemical Formula	Ion Adduct	m/z-value	Retention Time (min)	Optimization	Validation
DL-Maleic acid	$C_4H_4O_4$	[M-H]-	115.00368	1.5		
DL-methionine sulfoxide	$C_5H_{11}NO_3S$	[M+H]+	166.05324	0.93		
dodecanedioic acid	$C_{12}H_{22}O_4$	[M-H]-	229.14453	9.44		x
dodecenoylcarnitine (12:1)	$C_{19}H_{38}NO_4$	[M+H]+	342.26389	11.61		
dopamine	$C_8H_{11}NO_2$	[M+H]+	154.08626	1.48		
D-pantothenic acid	$C_9H_{17}NO_5$	[M+H]+	220.11795	6.5	x	x
D-quinic acid	$C_7H_{12}O_6$	[M+H]+	193.07066	1.06		
epinephrine	$C_9H_{13}NO_3$	[M+H]+	184.09682	1.27		
equol	$C_{15}H_{14}O_3$	[M+H]+	243.10215	11.3		
ethanolamine	C_2H_7NO	[M+H]+	62.06004	0.79		
ethyl heptanoate	$C_9H_{18}O_2$	[M+H]+	159.13796	13.5		
fructose/galactose	$C_6H_{12}O_6$	[M-H]-	179.05611	0.89	x	
fumaric acid	$C_4H_4O_4$	[M-H]-	115.00368	1.9		
furfural	$C_5H_4O_2$	[M+H]+	97.02841	6.2		
GABA	$C_4H_9NO_2$	[M+H]+	104.07061	0.92	x	x
gamma-butyrolactone	$C_4H_6O_2$	[M+H]+	87.04406	2.5		
gamma-glutamylphenylalanine	$C_{14}H_{18}N_2O_5$	[M+H]+	295.12884	8.03	x	x
Glucitol	$C_6H_{14}O_6$	[M+H]+	183.08631	1		
gluconic acid	$C_6H_{12}O_7$	[M-H]-	195.05103	0.95	x	
glucose	$C_6H_{12}O_6$	[M+Na]+	203.05261	0.91	x	x
glutaric acid	$C_5H_8O_4$	[M-H]-	131.03498	3.6		
glyceric acid	$C_3H_6O_4$	[M-H]-	105.01779	1.09	x	x
glycerol	$C_3H_8O_3$	[M+H]+	93.05462	1	x	
glycerolphosphorylcholine	$C_8H_20NO_6P$	[M+H]+	258.11010	0.97		
glycoursodeoxycholic acid (GUDCA)	$C_{26}H_{43}NO_5$	[M-H]-	448.30685	12.02	x	
guanine	$C_5H_5N_5O$	[M+H]+	152.05669	1.36		x
hexadecamide	$C_{16}H_{33}NO$	[M+H]+	256.26349	15.18	x	x
hippuric acid	$C_9H_9NO_3$	[M+H]+	180.06529	8.45	x	x

Analytical standard	Chemical Formula	Ion Adduct	m/z-value	Retention Time (min)	Optimization	Validation
homocysteine	$C_4H_9NO_2S$	[M+H]+	136.04268	1.13	x	x
hydroquinone	$C_6H_6O_2$	[M+H]+	111.04406	6.9	x	
hypoxanthine	$C_5H_4N_4O$	[M+H]+	137.04579	1.48	x	x
imidazolepropionic acid	$C_6H_8N_2O_2$	[M+H]+	141.06585	1.38		x
indole	C_8H_7N	[M+H]+	118.06513	11.88		
isobutyrylcarnitine	$C_{11}H_{21}NO_4$	[M+H]+	232.15386	6.48	x	x
kynurenic acid	$C_{10}H_7NO_3$	[M+H]+	190.04987	7.83	x	x
kynurenine	$C_{10}H_{12}N_2O_3$	[M+H]+	209.09207	4.55	x	x
L3-phenylacetic acid	$C_8H_8O_2$	[M+H]+	137.05971	8.89		
lactic acid	$C_3H_6O_3$	[M-H]-	89.0244173	1.43	x	x
L-arginine	$C_6H_{14}N_4O_2$	[M+H]+	175.11895	0.78	x	x
L-asparagine	$C_4H_8N_2O_3$	[M+H]+	133.06077	0.85		
L-carnitine	$C_7H_{15}NO_3$	[M+H]+	162.1125	0.87		x
L-citrulline	$C_6H_{13}N_3O_3$	[M+H]+	176.10297	0.97	x	x
L-dehydroascorbic acid	$C_6H_6O_6$	[M-H]-	173.00916	1.16		x
leucylglycine	$C_8H_{16}N_2O_3$	[M+H]+	189.12337	3.8		
L-glutamic acid	$C_5H_9NO_4$	[M+H]+	148.06043	0.88		
L-glutamine	$C_5H_{10}N_2O_3$	[M+H]+	147.07642	0.86	x	x
L-gulonic acid gamma lactone	$C_6H_{10}O_6$	[M-H]-	177.04046	0.9		
L-histidine	$C_6H_9N_3O_2$	[M+H]+	156.07675	0.85		
linoleamide	$C_{18}H_{33}NO$	[M+H]+	280.26349	14.71	x	x
L-isoleucine	$C_6H_{13}NO_2$	[M+H]+	132.10191	2.2	x	x
L-leucine	$C_6H_{13}NO_2$	[M+H]+	132.10191	2.4	x	x
L-lysine	$C_6H_{14}N_2O_2$	[M+H]+	147.1128	0.75		x
L-methionine	$C_5H_{11}NO_2S$	[M+H]+	150.05833	1.42	x	x
L-methionine sulfone	$C_5H_{11}NO_4S$	[M+H]+	182.04816	1.02		
L-ornithine	$C_5H_{12}N_2O_2$	[M-H]-	131.0826	0.9		
L-phenylalanine	$C_9H_{11}NO_2$	[M+H]+	166.08626	5.09	x	x
L-proline	$C_5H_9NO_2$	[M+H]+	116.07061	0.95	x	

Analytical standard	Chemical Formula	Ion Adduct	m/z-value	Retention Time (min)	Optimization	Validation
L-pyroglutamic acid	$C_5H_7NO_3$	[M+H]+	130.04987	1.6	x	x
L-serine	$C_3H_7NO_3$	[M+H]+	106.04987	0.84		
L-threonine	$C_4H_9NO_3$	[M+H]+	120.06552	0.87		x
L-tyrosine	$C_9H_{11}NO_3$	[M+H]+	182.08117	1.83	x	x
L-valine 5-aminovaleric acid	$C_5H_{11}NO_2$	[M+H]+	118.08626	1.13		x
lyso-phosphatidylcholine (18:0)	$C_{26}H_{52}NO_7P$	[M+H]+	522.35542	13.67		x
malic acid	$C_4H_6O_5$	[M-H]-	133.01424	1.19		x
malonylcarnitine	$C_{10}H_{17}NO_6$	[M+H]+	248.11286	1.13	x	x
mannose	$C_6H_{12}O_6$	[M-H]-	179.05611	1	x	x
methyl cyclohexanecarboxylate	$C_8H_{14}O_2$	[M+H]+	143.10666	12.56		
methyl isobutyrate	$C_5H_{10}O_2$	[M+H]+	103.07536	11.09		
methylsuccinic acid	$C_5H_8O_4$	[M+H]+	133.04954	0.99		
myo-inositol	$C_6H_{12}O_6$	[M-H]-	179.05611	0.86		
myristoleic acid	$C_{14}H_{26}O_2$	[M+H]+	227.20056	14.3		
N,N-dimethylarginine	$C_8H_{18}N_4O_2$	[M+H]+	203.15025	0.93	x	x
N6-methyladenosine	$C_{11}H_{15}N_5O_4$	[M+H]+	282.11968	4.2		x
N-acetyl galactosamine	$C_8H_{15}NO_6$	[M+H]+	222.09721	1.05		
N-acetylagmatine	$C_7H_{16}N_4O$	[M+H]+	173.13951	1.55		
N-acetylarginine	$C_8H_{16}N_4O_3$	[M+H]+	217.12952	1.16		x
N-acetylasparagine	$C_6H_{10}N_2O_4$	[M+H]+	175.07133	1.16	x	x
N-acetyl-beta-alanine	$C_5H_9NO_3$	[M+H]+	132.06552	1.68		x
N-acetylglutamic acid	$C_7H_{11}NO_5$	[M+H]+	190.071	1.76	x	x
N-acetylglycine	$C_4H_7NO_3$	[M+H]+	118.04987	1.5	x	
N-acetyl-L-methionine	$C_7H_{13}NO_3S$	[M+H]+	192.06889	7.6	x	x
N-acetyl-L-phenylalanine	$C_{11}H_{13}NO_3$	[M+H]+	208.09682	10.17	x	x
N-acetyl-L-proline	$C_7H_{11}NO_3$	[M+H]+	158.08117	6.34		
N-acetyltryptophan	$C_{13}H_{14}N_2O_3$	[M+H]+	247.1077	10.42	x	x
N-acetyltyrosine	$C_{11}H_{13}NO_4$	[M+H]+	224.09173	7.67	x	x

Analytical standard	Chemical Formula	Ion Adduct	m/z-value	Retention Time (min)	Optimization	Validation
N-acetylvaline/N-isovalerylglycine	$C_7H_{13}NO_3$	[M+H]+	160.09682	7.6	x	x
nicotinamide	$C_6H_6N_2O$	[M+H]+	123.05530	1.59	x	x
nicotinic acid/picolinic acid	$C_6H_5NO_2$	[M+H]+	124.0393	1.42	x	
N-nitrosodiethylamine	$C_4H_{10}N_2O$	[M+H]+	103.08659	2.1		
N-palmitoylglycine	$C_{18}H_{35}NO_3$	[M+H]+	314.26897	14.47		
N-tigloylglycine	$C_7H_{11}NO_3$	[M+H]+	158.08117	7.29		
O-Acetyl-L carnitine	$C_9H_{17}NO_4$	[M+H]+	204.12303	1.5		x
octanoyl-L-carnitine (8:0)	$C_{15}H_{29}NO_4$	[M+H]+	288.21693	10.89	x	
oleamide	$C_{18}H_{35}NO$	[M+H]+	282.27914	15.34	x	x
oleoyl-L-carnitine	$C_{25}H_{47}NO_4$	[M+H]+	426.35779	12.72		
ophthalmic acid	$C_{11}H_{19}N_3O_6$	[M+H]+	290.134436	1.65	x	
O-succinylcarnitine	$C_{11}H_{19}NO_6$	[M+H]+	262.12851	1.75	x	x
oxoglutaric acid	$C_5H_6O_5$	[M+H]+	147.02880	1.4	x	x
pantothenol	$C_9H_{19}NO_4$	[M+H]+	206.13868	6.63	x	x
phenyl actic acid	$C_8H_8O_2$	[M-H]-	135.04515	10.04	x	
phenylacetaldehyde	C_8H_8O	[M+H]+	121.06479	11		x
phenylAc-Gln-OH	$C_{13}H_{16}N_2O_4$	[M+H]+	265.11828	8.54	x	x
phosphocholine calcium salt tetrahydrate	$C_5H_{13}CaClNO_4P\cdot{}_4H_2O$	[M+H]+	184.07339	0.94	x	
pimelic acid	$C_7H_{12}O_4$	[M-H]-	159.06628	6.5	x	
pipecolic acid	$C_6H_{11}NO_2$	[M+H]+	130.08626	1.4	x	
piperine	$C_{17}H_{19}NO_3$	[M+H]+	286.14377	12.34		
propionyl carnitine C3	$C_{10}H_{19}NO_4$	[M+H]+	218.13868	3.01		x
putrescine	$C_4H_{12}N_2$	[M+H]+	89.10732	0.75	x	
pyridine	C_5H_5N	[M+H]+	80.04948	1		
pyrrole-2-carboxylic acid	$C_5H_5NO_2$	[M+H]+	112.03930	7.14	x	
pyruvic acid	$C_3H_4O_3$	[M-H]-	87.00877	1.16	x	x
rac-glycerol 1-phosphate sodium salt hydrate	$C_3H_9O_6P$	[M+H]+	173.02095	1	x	

Analytical standard	Chemical Formula	Ion Adduct	m/z-value	Retention Time (min)	Optimization	Validation
saccharic acid	$C_6H_{10}O_8$	[M-H]-	209.03029	1.04	x	x
S-adenosylhomocystein	$C_{14}H_20N_6O_5S$	[M+H]+	385.12886	2.13	x	
sarcosine	$C_3H_7NO_2$	[M+H]+	90.05496	0.85		
sebacic acid	$C_{10}H_{18}O_4$	[M-H]-	201.11323	9.03	x	x
serotonin	$C_{10}H_{12}N_2O$	[M+H]+	177.10224	3.7		
sinapic acid	$C_{11}H_{12}O_5$	[M-H]-	223.0612	10.2		
sodium glycochenodeoxycholate (GCDCA)	$C_{26}H_{42}NNaO_5$	[M-H]-	391.28534	12.03		
sodium glycocholate (GCA)	$C_{26}H_{42}NNaO_6$	[M-H]-	464.30176	11.47		
sodium glycodeoxycholate (GDCA)	$C_{26}H_{42}NO_5Na$	[M-H]-	448.30685	12.18	x	
sodium taurocholate (TCA)	$C_{26}H_{44}NNaO_7S$	[M-H]-	514.28440	11.26	x	x
sodium taurodeoxycholate hydrate (TDCA)	$C_{26}H_{44}NO_6SNa$	[M-H]-	498.28948	11.78		
spermidine	$C_7H_{19}N_3$	[M+H]+	146.16517	0.75	x	x
spermine	$C_{10}H_{26}N_4$	[M+H]+	203.22302	0.7		
stearoyl-L-carnitine	$C_{25}H_{49}NO_4$	[M+H]+	428.37240	13.09		
succinic acid	$C_4H_6O_4$	[M-H]-	117.01933	1.96	x	x
sucrose	$C_{12}H_{22}O_{11}$	[M-H]-	341.10893	1.02		
syringic acid	$C_9H_{10}O_5$	[M+H]+	199.0601	7		
taurine	$C_2H_7NO_3S$	[M+H]+	126.02190	0.93		
taurochenodeoxycholic acid sodium salt (TCDCA)	$C_{26}H_{44}NO_6SNa$	[M-H]-	498.28948	11.65		
testosterone	$C_{19}H_{28}O_2$	[M+H]+	289.21621	12.47		
tetradecanoylcarnitine (14:0)	$C_{21}H_{41}NO_4$	[M+H]+	372.31084	12.24		
thiabendazole	$C_{10}H_7N_3S$	[M+H]+	202.04334	8.4		
thiazolidine-4-carboxylic acid	$C_4H_7NO_2S$	[M+H]+	134.02703	1		
threonic acid	$C_4H_8O_5$	[M+H]+	137.04445	0.95		x
thymine	$C_5H_6N_2O_2$	[M+H]+	127.0502	2.6		
tolbutamide	$C_{12}H_{18}N_2O_3S$	[M+H]+	271.11110	11.75		

Analytical standard	Chemical Formula	Ion Adduct	m/z-value	Retention Time (min)	Optimization	Validation
trans, trans-2,4decadienal	$C_{10}H_{16}O$	[M+H]+	153.12739	13.27		
trans-2-octenoylcarnitine	$C_{15}H_{27}NO_4$	[M+H]+	286.20128	10.81		
trans-3-octen-2-one	$C_8H_{14}O$	[M+H]+	127.11174	12.16		
trans-4-hydroxy-l-proline	$C_5H_9NO_3$	[M+H]+	132.06552	0.9		
trans-hexadec-2-enoyl carnitine	$C_{23}H_{43}NO_4$	[M+H]+	398.32549	12.44		
trehalose	$C_{12}H_{22}O_{11}$	[M+H]+	360.15004	1.04	x	
Triethanolamine hydrochloride	$C_6H_{16}ClNO_3$	[M+H]+	150.11247	0.87		
tryptamine	$C_{10}H_{12}N_2$	[M+H]+	161.10732	7.97		
tryptophan	$C_{11}H_{12}N_2O_2$	[M+H]+	205.09715	7.5	x	x
tyramine	$C_8H_{11}NO$	[M+H]+	138.09134	2.35		
UDCA	$C_{24}H_40O_4$	[M-H]-	391.28538	12.09		
uracil	$C_4H_4N_2O_2$	[M+H]+	113.03455	1.42	x	
Uridine	$C_9H_{12}N_2O_6$	[M+H]+	245.07681	1.47		
urocanic acid	$C_6H_6N_2O_2$	[M+H]+	139.0502	1.41	x	
valerylcarnitine	$C_{12}H_{23}NO_4$	[M+H]+	246.16940	8.2	x	x
vanillic acid	$C_8H_8O_4$	[M+H]+	169.04954	8.7		
L-valine/aminovaleric acid	$C_5H_{11}NO_2$	[M+H]+	118.08626	1.1		x

Table S7: Instrumental parameters for UHPLC-HRMS analysis

**Instrumental parameters**	**Settings**
mass spectrometer	Q-Exactive™ stand-alone bench top quadrupole-Orbitrap high-resolution mass spectrometer ((Thermo FisherScientific, San José, CA, USA)
liquid chromatographer	Dionex UltiMate 3000 XRS UHPLC system (Thermo FisherScientific, San José, CA, USA)
ionization source/mode	HESI-II/ positive and negative
column type	Acquity HSS T3 C18 column (1.8µm, 150 x 2.1mm)
column temperature	45°C
flow rate	0.4 ml min^{-1}
binary solvent	UPW (A) and acetonitrile (B) with 0.1 % formic acid
gradient profile (v/v of solvent A)	0−1.5 min at 98 %, 1.5−7.0 min from 98 % to 75 %, 7.0−8.0 min from 75 % to 40 %, 8.0−12.0 min from 40 % to 5 %, 12.0−14.0 min at 5 %, 14.0−14.1 min from 5 % to 98 %; 4 min re-equilibration
injection volume	10 µl
sheath gas flow rate	50 a.u.
auxiliary gas flow rate	25 a.u.
sweep gas flow rate	5 a.u.
heater temperature	350°C
capillary temperature	250°C
S-lens RF level	50%
spray voltage	3 kV (pos) and 2 kV (neg)
m/z scan range	53-800 Da
automatic gain control target	1 x 106 ions
maximum injection time	70 ms
acquisition mode	full scan
mass resolution settings	140 000 FWHM (1 Hz)

a.u.: arbitrary units, UHPLC: ultrahigh performance liquid chromatography, UPW: ultrapure water, FWHM: full width half maximum

Table S8: List of the targeted metabolites (n = 101) in the adult dog cohort for comparison of IE with non-brain related diseases.

**Targeted metabolite**	**Chemical Formula**	**Ion Adduct**	**m/z-value**	**Retention Time (min)**
1,3-propanediol	$C_3H_8O_2$	[M+H]+	77.05971	1.33
2-hydroxyhexanoic acid	$C_6H_{12}O_3$	[M-H]-	131.0695	9.4
2-hydroxyisocaproic acid	$C_6H_{12}O_3$	[M+H]+	131.06995	9.16
2-hydroxy 3-methylvalerate	$C_6H_{12}O_3$	[M-H]-	131.06950	9.03
2-methylbutyryl-L-carnitine	$C_{12}H_{23}NO_4$	[M+H]+	246.16998	8.55
2-phenylethylamine	$C_8H_{11}N$	[M+H]+	122.09643	6.59
2-piperidinone	C_5H_9NO	[M+H]+	100.07569	5
3-indoleacetic acid	$C_10H_9NO_2$	[M+H]+	176.07061	8.84
3-methoxytyramine hydroxychloride	$C_9H_{13}NO_2$	[M+H]+	168.10191	4.4
3-methylbutyryl/isovalaryl-L-carnitine	$C_{12}H_{23}NO_4$	[M+H]+	246.16998	8.21
4-guanidinobutyric acid	$C_5H_{11}N_3O_2$	[M+H]+	146.09220	1.44
4-hydroxyphenylpyruvic acid	$C_9H_8O_4$	[M-H]-	179.03498	4.55
4-methyl-2-oxovaleric acid	$C_6H_10O_3$	[M-H]-	129.05432	8.41
5-hydroxyindole-3-acetic acid	$C_10H_9NO_3$	[M+H]+	192.06552	8.04
6-acetyl-L-lysine	$C_8H_{16}N_2O_3$	[M+H]+	189.12337	1.47
7-methylguanine	$C_6H_7N_5O$	[M+H]+	166.07234	1.62
acetophenone	C_8H_8O	[M+H]+	121.06479	11.28
acetylcholine	$C_7H_{16}ClNO_2$	[M+H]+	146.11756	1.1
adenine	$C_5H_5N_5$	[M+H]+	136.06177	1.33
adenosine-5-monophosphate	$C_10H_{14}N_5O_7P$	[M+H]+	348.07036	1.47
adipoylcarnitine C16	$C_{13}H_{23}NO_6$	[M+H]+	290.15981	6.16
alfa-hydroxyisobutyric acid	$C_4H_8O_3$	[M+H]+	105.05462	2.5
allantoin	$C_4H_6N_4O_3$	[M+H]+	159.05127	1
anandamide (18:1)	$C_20H_{39}NO_2$	[M+H]+	326.30536	15.04
azelaic acid	$C_9H_{16}O_4$	[M-H]-	187.09758	8.83

B-hydroxyisovaleric acid	$C_5H_{1}0O_3$	[M-H]-	117.05572	3.9
butyrylcarnitine	$C_{11}H_{21}NO_4$	[M+H]+	232.15433	6.58
chenodeoxycholic acid (CDCA)	$C_{24}H_{4}0O_4$	[M-H]-	391.28534	12.9
cholic acid	$C_{24}H_{4}0O_5$	[M-H]-	407.2803	11.99
choline	$C_5H_{14}NO$	[M+H]+	104.10699	0.83
corticosterone	$C_{21}H_{3}0O_4$	[M+H]+	347.22170	11.54
cortisol	$C_{21}H_{3}0O_5$	[M+H]+	363.21660	11.17
creatine	$C_4H_9N_3O_2$	[M+H]+	132.0768	0.94
creatinine	$C_4H_7N_3O$	[M+H]+	114.0661	0.89
cytidine	$C_9H_{13}N_3O_5$	[M+H]+	244.0928	1.32
cytosine	$C_4H_5N_3O$	[M+H]+	112.05054	1.5
Deoxycholic acid (DCA)	$C_{24}H_{4}0O_4$	[M-H]-	391.28534	13.05
dipropyl disulfide	$C_6H_{14}S_2$	[M+H]+	151.06097	1.5
dodecanedioic acid	$C_{12}H_{22}O_4$	[M-H]-	229.14453	9.44
D-pantothenic acid	$C_9H_{17}NO_5$	[M+H]+	220.11795	6.5
GABA	$C_4H_9NO_2$	[M+H]+	104.07061	0.92
gamma-glutamylphenylalanine	$C_{14}H_{18}N_2O_5$	[M+H]+	295.12884	8.03
glucose	$C_6H_{12}O_6$	[M+Na]+	203.05261	0.91
glyceric acid	$C_3H_6O_4$	[M-H]-	105.01779	1.09
hexadecamide	$C_{16}H_{33}NO$	[M+H]+	256.26349	15.18
hippuric acid	$C_9H_9NO_3$	[M+H]+	180.06529	8.45
homocysteine	$C_4H_9NO_2S$	[M+H]+	136.04268	1.13
hypoxanthine	$C_5H_4N_4O$	[M+H]+	137.04579	1.48
imidazolepropionic acid	$C_6H_8N_2O_2$	[M+H]+	141.06585	1.38
isobutyrylcarnitine	$C_{11}H_{21}NO_4$	[M+H]+	232.15386	6.48
kynurenic acid	$C_{1}0H_7NO_3$	[M+H]+	190.04987	7.83
kynurenine	$C_{1}0H_{12}N_2O_3$	[M+H]+	209.09207	4.55
lactic acid	$C_3H_6O_3$	[M-H]-	89.0244173	1.43
L-arginine	$C_6H_{14}N_4O_2$	[M+H]+	175.11895	0.78
L-carnitine	$C_7H_{15}NO_3$	[M+H]+	162.1125	0.87

L-citrulline	$C_6H_{13}N_3O_3$	[M+H]+	176.10297	0.97
L-dehydroascorbic acid	$C_6H_6O_6$	[M-H]-	173.00916	1.16
L-glutamine	$C_5H_10N_2O_3$	[M+H]+	147.07642	0.86
linoleamide	$C_{18}H_{33}NO$	[M+H]+	280.26349	14.71
L-isoleucine	$C_6H_{13}NO_2$	[M+H]+	132.10191	2.2
L-leucine	$C_6H_{13}NO_2$	[M+H]+	132.10191	2.4
L-lysine	$C_6H_{14}N_2O_2$	[M+H]+	147.1128	0.75
L-methionine	$C_5H_{11}NO_2S$	[M+H]+	150.05833	1.42
L-phenylalanine	$C_9H_{11}NO_2$	[M+H]+	166.08626	5.09
L-pyroglutamic acid	$C_5H_7NO_3$	[M+H]+	130.04987	1.6
L-threonine	$C_4H_9NO_3$	[M+H]+	120.06552	0.87
L-tyrosine	$C_9H_{11}NO_3$	[M+H]+	182.08117	1.83
L-valine 5-aminovaleric acid	$C_5H_{11}NO_2$	[M+H]+	118.08626	1.13
lyso-phosphatidylcholine (18:0)	$C_{26}H_{52}NO_7P$	[M+H]+	522.35542	13.67
malic acid	$C_4H_6O_5$	[M-H]-	133.01424	1.19
malonylcarnitine	$C_10H_{17}NO_6$	[M+H]+	248.11286	1.13
mannose	$C_6H_{12}O_6$	[M-H]-	179.05611	1
N,N-dimethylarginine	$C_8H_{18}N_4O_2$	[M+H]+	203.15025	0.93
N6-methyladenosine	$C_{11}H_{15}N_5O_4$	[M+H]+	282.11968	4.2
N-acetylarginine	$C_8H_{16}N_4O_3$	[M+H]+	217.12952	1.16
N-acetylasparagine	$C_6H_10N_2O_4$	[M+H]+	175.07133	1.16
N-acetyl-beta-alanine	$C_5H_9NO_3$	[M+H]+	132.06552	1.68
N-acetylglutamic acid	$C_7H_{11}NO_5$	[M+H]+	190.071	1.76
N-acetyl-L-methionine	$C_7H_{13}NO_3S$	[M+H]+	192.06889	7.6
N-acetyl-L-phenylalanine	$C_{11}H_{13}NO_3$	[M+H]+	208.09682	10.17
N-acetyltryptophan	$C_{13}H_{14}N_2O_3$	[M+H]+	247.1077	10.42
N-acetyltyrosine	$C_{11}H_{13}NO_4$	[M+H]+	224.09173	7.67
N-acetylvaline/N-isovalerylglycine	$C_7H_{13}NO_3$	[M+H]+	160.09682	7.6
nicotinamide	$C_6H_6N_2O$	[M+H]+	123.05530	1.59
O-Acetyl-L carnitine	$C_9H_{17}NO_4$	[M+H]+	204.12303	1.5

oleamide	$C_{18}H_{35}NO$	[M+H]+	282.27914	15.34
O-succinylcarnitine	$C_{11}H_{19}NO_6$	[M+H]+	262.12851	1.75
oxoglutaric acid	$C_5H_6O_5$	[M+H]+	147.02880	1.4
pantothenol	$C_9H_{19}NO_4$	[M+H]+	206.13868	6.63
phenylacetaldehyde	C_8H_8O	[M+H]+	121.06479	11
phenylAc-Gln-OH	$C_{13}H_{16}N_2O_4$	[M+H]+	265.11828	8.54
propionyl carnitine C3	$C_{1}0H_{19}NO_4$	[M+H]+	218.13868	3.01
pyruvic acid	$C_3H_4O_3$	[M-H]-	87.00877	1.16
saccharic acid	$C_6H_{1}0O_8$	[M-H]-	209.03029	1.04
sebacic acid	$C_{1}0H_{18}O_4$	[M-H]-	201.11323	9.03
sodium taurocholate (TCA)	$C_{26}H_{44}NNaO_7S$	[M-H]-	514.28440	11.26
spermidine	$C_7H_{19}N_3$	[M+H]+	146.16517	0.75
succinic acid	$C_4H_6O_4$	[M-H]-	117.01933	1.96
threonic acid	$C_4H_8O_5$	[M+H]+	137.04445	0.95
tryptophan	$C_{11}H_{12}N_2O_2$	[M+H]+	205.09715	7.5
valerylcarnitine	$C_{12}H_{23}NO_4$	[M+H]+	246.16940	8.2

Figure S1: Boxplot illustrating the iQC normalized peak areas for acetophenone in the control and IE group.

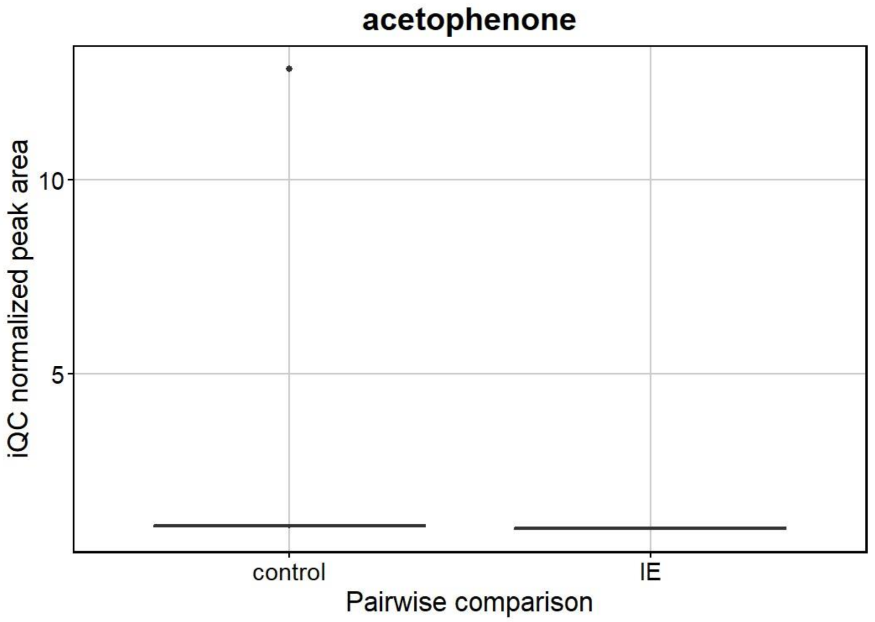

Figure S2: Fingerprint of combined positive and negative component ions upon UHPLC-HRMS analysis of canine cerebrospinal fluid (n = 15). The putatively identified metabolite (norepinephrine) is indicated in red.

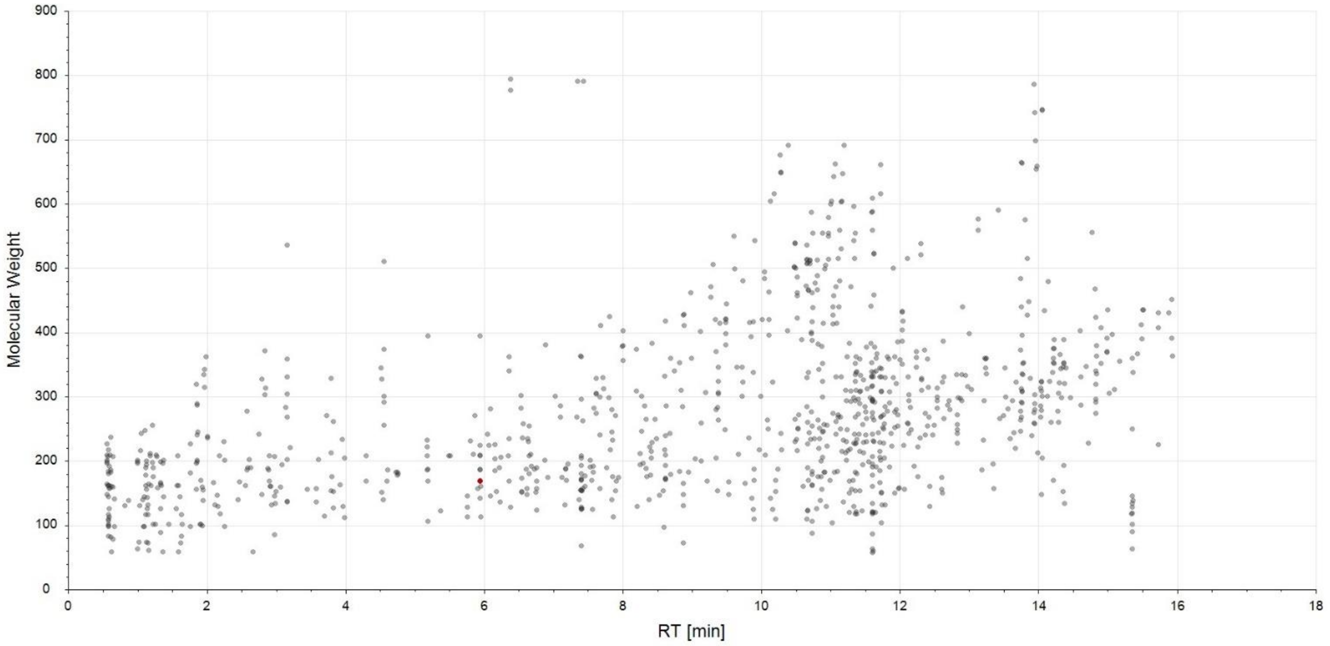

Figure S3: Scatterplot illustrating the iQC normalized peak areas for cortisol on the y-axis and number of days between sampling and the last seizure event on the x-axis.

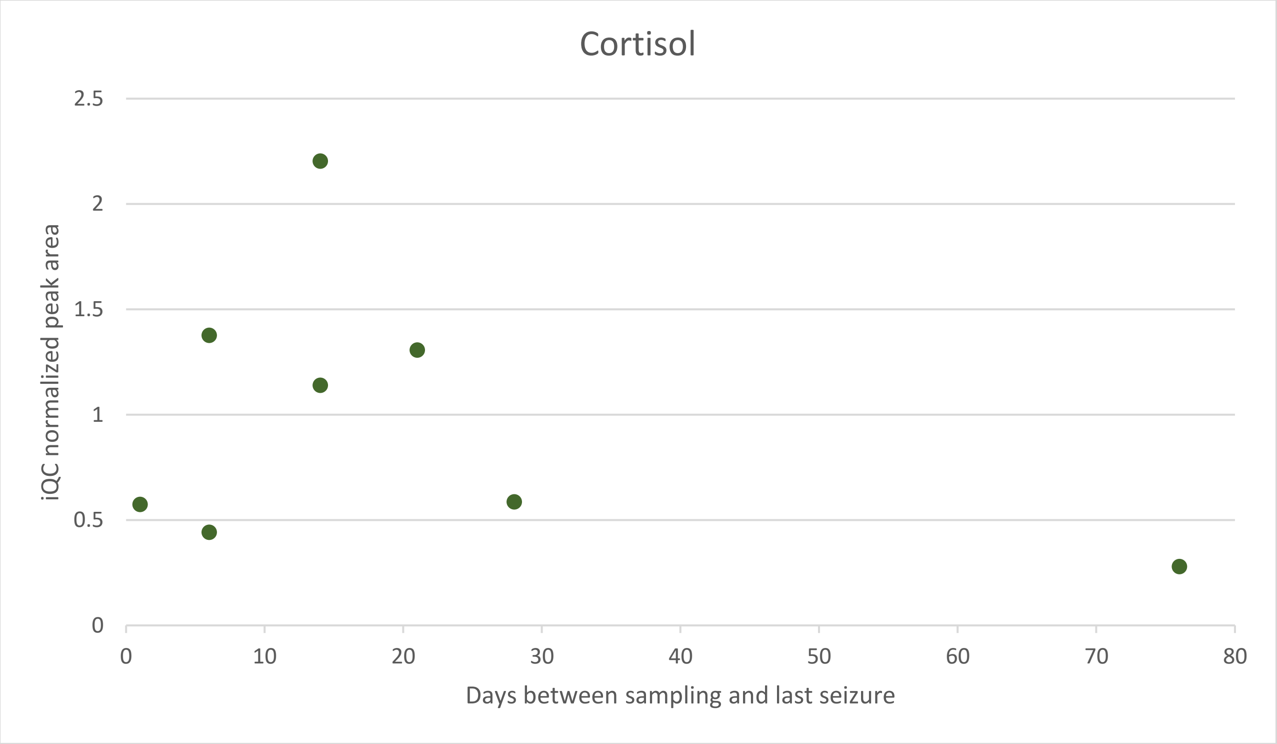

Supplement: Supplementary file 1 — Supplementary Information. [file 41598_2026_48734_MOESM1_ESM.pdf]
